# 16th Annual Meeting of the European Musculo-Skeletal Oncology Society & 4th Symposium of the EMSOS Nurse Group, 7–9 May 2003, Budapest: Abstracts

**DOI:** 10.1080/13577140310001607338

**Published:** 2003-06


*Sarcoma*, June 2003, *VOL. 7, NO. 2, 93–129*

Taylor & Francis healthsciences

### 16th Annual Meeting of the European Musculo-Skeletal Oncology Society & 4th Symposium of the EMSOS Nurse Group, 7–9 May 2003, Budapest: Abstracts

**Quality of Life in Patients with Limb Sarcoma after Palliative Amputation**

Z. Shmuely, Y. Kollender, J. Bickels, I. Meller, O. Merimsky
*(Unit of Bone and Soft Tissue Oncology, Division of Oncology, and The National Unit of Orthopedic Oncology, The Tel-Aviv Sourasky Medical Center Tel-Aviv, Israel 64239)*

*Background:* Limb sparing surgery has replaced the amputation surgery in the treatment of limb sarcomas. Recurrent or persistent disease constitutes a major problem. Local symptoms such as agonizing pain, fractures, tumor fungation, inability to walk and inability to maintain daily activities, further impair the patient's quality of life. In this clinical set-up palliative amputation should be considered.
*Methods:* Eighteen patients with soft-tissue or bone sarcomas and three patients with metastatic carcinoma underwent palliative major amputation. Hemipelvectomy was performed in three patients, hip disarticulation in 10 patients, knee disarticulation or below-knee amputation in three patients, shoulder disarticulation in one patient, forequarter amputation in four patients.
*Results:* Local control of the disease and pain, and improvement of the performance status were observed in 19 evaluable patients. The mobility was restored in 15 patients with lower limb surgery. The median survival following the procedure was 9 months. There was only one case of immediate post-operative death. Severe phantom pain was not reported by any of the patients. Quality of life was reported to be improved by two-thirds of the patients.
*Conclusion:* Our data point to the fact that palliative amputation surgery is feasible, not associated with increased mortality, and is worth-performing in low-performance status cancer patients with locally advanced disease. Local symptoms and signs were controlled, and quality of life was restored. In our opinion, pain alone does constitute an indication for palliative major amputation, especially if it is accompanied by pain-related limb dysfunction or low performance status.

**Characterization and Clinical Value of the Methylenetetrahydrofolate Reductase (MTHFR) Gene Polymorphisms in Osteosarcoma Pediatric Patients**

A. Patiño-Garcia, N. Jansen, A. Winkelhagen, E. Paz-Curbera, M. San Julian, L. Sierrasesumaga
*(Deptartments of Pediatrics and Orthopaedics, University Clinic and University of Navarra, Pamplona, Spain)*

*Aim:* The main prognostic factor for pediatric osteosarcoma is the response to chemotherapy, based on the administration of different drugs including high-dose methotrexate (MTX). MTX is an antifolate agent that interferes in the synthesis of nucleic acids either by directly inhibiting dihydrofolate reductase (DHFR) or indirectly acting on the methylenetetrahydrofolate reductase (MTHFR), which regulate the availability and distribution of folates within the cell.
*Materials and methods:* We have analyzed the C667T and A1298C genetic polymorphisms of the MTHFR gene in a group of 83 osteosarcoma pediatric patients and 83 sex and age paired controls. The clinical variables with prognostic value and those related to the treatment-induced toxicity have been gathered.
*Results:* We have not detected differences in the allele and genotype frequencies for the C667T and A1298C polymorphisms between patients and controls. These two polymorphisms are in strict linkage disequilibrium in our series ($P < 0.0001$). The presence of one or both polymorphisms was neither related to overall survival or to the response to treatment evaluated as the degree of necrosis induced by the antitumoral treatment. Nevertheless, those patients who were homozygous for the polymorphic form of the C667T marker (TT) tended to have more renal toxicity (both acute and chronic) than normal (CC) or heterozygous (CT) osteosarcoma patients ($P = 0.058$). The clinical variables associated with poor prognosis in our osteosarcoma series were the chondroblastic subtype ($P = 0.05$), the central localization of the primary tumor ($P < 0.001$), the presence of metastases ($P = 0.002$) and less than 90% necrosis after induction chemotherapy ($P = 0.03$).
*Conclusions:* The presence of the C677T and A1298C polymorphisms of the MTHFR gene is not associated with survival in osteosarcoma, although the C677T polymorphic genotype seems to influence MTX toxicity as it has been described for breast cancer and following bone marrow transplantation.

**Genetic and Epigenetic Alterations in Pediatric Osteosarcoma**

A. Patiño-Garcia, M. San Julian, M. Zalacain, L. Gárate, L. Sierrasesúmaga
*(Departments of Pediatrics and Orthopaedics, University Clinic, University of Navarra, Pamplona 31080, Spain)*

*Background:* Osteosarcoma is the most common malignant bone tumor of adolescence and childhood. At the molecular level, pediatric osteosarcoma is a puzzle of genetic alterations and it probably arises from the dysregulation of the cell cycle due to alterations in the TP53 and RB1 pathways and, therefore, in the arrest of the cell-cycle in response to DNA damage.
*Methods:* Peripheral blood samples and clinical data were available from 76 osteosarcoma patients. Paired tissue was available from 41 of them. The mutation and methylation status of p16INK4, p21WAF1, TP53, RB1 was screened as well as LOH (Loss of Heterozygosity) at 3q and 18q.
*Results:* The results of the LOH analysis at 3q, 13q (RB1), 17p (TP53) and 18q in our series were:

| | | 17p (TP53) | 13q (RB1) | 18q | 3q |
|---|---|---|---|---|---|
| Informative | Normal | 57.7% (15/26) | 62.8% (22/35) | 69.2% (27/39) | 87.8% (36/41) |
| | Altered | 42.3% (11/26) | 37.2% (13/35) | 30.8% (12/39) | 12.2% (5/41) |
| Non-informative | | 36.6% (15/41) | 14.6% (6/41) | 4.8% (2/41) | 0% (0/41) |

ISSN 1357-714X print/1369–1643 © 2003 Taylor & Francis Ltd
DOI: 10.1080/13577140310001607338

Alteration (LOH) at the RB1 locus tended to be associated with poor prognosis in this osteosarcoma series ($P = 0.053$). The median event-free survival was 40 months for patients without LOH and 16 months for patients with RB1 LOH ($P = 0.023$). The hemizygous deletion at 3q, 18q or 17p was not associated with survival or event-free survival in our patients. Mutations affecting exons 5 to 8 of the TP53 gene were present in 16/41 (39%) of the osteosarcoma tissue samples. Genetic and epigenetic alterations of p21WAF1 and p16INK4 were not frequent in our series.
*Conclusions:* RB1, TP53 and possibly other tumor suppressor genes located at 18q and other localizations are involved in pediatric osteosarcoma carcinogenesis together with other genetic alterations not fully understood to date. Inhibition of gene expression by RB1, p16INK4 and p21WAF1 promoter hypermethylation does not seem to be preferentially involved in the carcinogenesis of pediatric osteosarcoma. The presence of an altered RB1 gene should be also regarded, according to our results, as a poor prognostic factor for pediatric osteosarcoma.

### DNA Flow Cytometry is a Prognostic Tool in Skeletal Metastases

R. Wedin, H.C.F. Bauer[1], B. Tribukait[2], J. Wejde[3]
*([1]Department of Orthopaedic, Karolinska Hospital, Stockholm SE-171 76, Sweden, [2]Cancer Centre, Karolinska Hospital, Stockholm SE-171 76, Sweden, [3]Department of Pathology, Karolinska Hospital, Stockholm SE-171 76, Sweden)*

Long-term survival after surgical treatment for pathologic fracture is associated with frequent failure of the reconstruction. DNA flow cytometry has previously been applied as a prognostic tool in kidney cancer and patients with diploid (normal DNA content) metastases had longer survival after pathologic fracture than those with aneuploid (abnormal DNA content). In a consecutive series of 89 kidney cancer patients operated for pathologic fracture 1987–1994 at our Department DNA flow cytometry was obtained in 31. The 1-year survival was equal for patients with diploid and aneuploid metastases. However, long-term survival was almost exclusively seen among patients with diploid lesions. Five of 13 patients with diploid lesions survived more than 3 years, as opposed to one of 17 with aneuploid metastases ($P = 0.06$). DNA flow cytometry was also obtained in 36 breast cancer patients operated for pathological fracture. Ten of the 36 patients had diploid and 26 had aneuploid tumors. A similar association was found in breast cancer as in kidney cancer, i.e., long-term survival was mostly seen in the group with diploid metastases. These results indicate that DNA flow cytometry may be applied to identify long-term survivors among patients with skeletal metastases of breast or kidney cancer. The results warrant routine collection of tumor samples for DNA cytometry at the time of surgery for pathologic fracture.

### Superiority of Postoperative Epidural over Intravenous Patient-Controlled Analgesia after Lower-Body Bone Malignancy Resection

Avi A. Weinbroun, Benjamin Bender, Jacob Bickels, Alexander Nirkin, Nissim Marouani, Isaac Meller, Yehuda Kollender
*(Tel Aviv Sourasky Medical Center, 6 Weizman street, Tel Aviv 64239, Israel)*

Pain after extensive bone malignancy resection is intense and requires large amounts of antinociceptives, especially if there had been pain preoperatively. Patients undergoing extensive bone malignancy resection under standardized combined general and epidural anesthesia randomly and prospectively received patient-controlled epidural anesthesia (PCEA, ropivacaine 3.2 mg plus fentanyl 8 μg/dose) or intravenous patient-controlled analgesia (IV-PCA, morphine 2 mg/dose) postoperatively ($n = 12$/group), starting at a subjective pain intensity of $^3$4/10 (visual analog scale). Postoperative analgesic delivery continued for up to 96 h. Intramuscular rescue diclofenac 75 mg was also available. The level of pain intensity among the PCEA patients was lower by ~40% ($P < 0.01$) compared to the IV-PCA patients. The overall maximal pain scores of the PCEA patients were 30% lower than those of the IV-PCA patients. The demand for diclofenac was significantly (40%, $P < 0.01$) lower for the PCEA patients compared to their IV counterparts, and the same difference applied to the overall reported side effects. Self-rated wakefulness and feelings of well being were also better in the PCEA patients. The time to first ambulation and home discharge was similar between the two groups. PCEA reduces pain better and affords better recovery than IV-PCA after extensive bone malignancy resection carried out under combined general and epidural anesthesia.

### Concomitant Dextromethorphan (DM) and Epidural Patient-Controlled Analgesia (PCA) Provides Better Pain- and Analgesics-Sparing Effects than DM and Intravenous PCA after Bone-Malignancy Resection. A Randomized, Placebo-Controlled, Double-Blind Study

Avi A. Weinbroum, Benjamin Bender, Jacob Bickels, Alexander Nirkin, Nissim Marouani, Isaac Meller, Yehuda Kollender
*(Tel Aviv Sourasky Medical Center, 6 Weizman street, Tel Aviv 64239, Israel)*

Pain after bone-malignancy surgery is intense and requires large amounts of analgesics. The adjutant antinociceptive effects of dextromethorphan (DM), an *N*-methyl-D-aspartate receptor antagonist, were demonstrated following general and epidural anesthesia. We now compared the use of postoperatively epidural (PCEA) versus intravenous patient-controlled (IV-PCA) analgesia in patients undergoing surgery for bone-malignancy under standardized combined-general and epidural anesthesia in the presence or absence of DM. Patients were randomly enrolled to receive ($n = 30$) PCEA (ropivacaine 3.2 mg plus fentanyl 8 μg/dose) or IV-PCA (morphine 2 mg/dose) postoperatively, starting at subjective pain intensity $^3$4/10 (visual analog scale) up to 96 h. Rescue drug (diclofenac 75 mg IM) was also available. Placebo or DM 90 mg orally (30 patients/group/set) was given double-blindly before surgery and for two subsequent days. DM patients used the PCA devices ~35% less than their placebo counter-parts in either set and pain was ~25% lower during the first two postoperative days ($P < 0.05$). In addition, pain intensity among the PCEA patients was lower ~50% than in the IV set ($P < 0.05$). The overall maximal pain rated in the PCEA set was half the intensity estimated by the IV set patients ($P < 0.05$). The demand for diclofenac was significantly ($P < 0.01$) lower for the PCEA-DM patients compared to their placebo and set counterparts. The overall reported incidence of side effects was two cases in the PCEA-DM individuals compared to eight in the IV-PCA-DM ($P < 0.05$); urinary catheter was similarly necessary in the IV-PCA and in the PCEA patients. Time to first ambulation was similar for either technique, but all DM patients ambu-lated earlier than the placebos.

### Prosthetic Reconstruction of Skeletal Defects due to Resection of Primary Bone Tumors with Turkish Muscuoskeletal Tumor Society (TMTS) Prosthesis

K. Erler, Mustafa Basbozkurt, Bahtiyar Demiralp, M.Taner Ozdemir, Ethem Gur
*(Department of Orthopedics and Traumatology, Gülhane Military Medical Academy, Etlik, Ankara 06018, Turkey)*

*Aim:* Saving the limbs functionally after resection of primary bone tumors has been a challenge for orthopedic surgeons.

Although selection of the most appropriate option in this wide spectrum of modalities requires careful management of the patient and the lesion. Endoprosthetic replacement after resection of primary bone tumors is a widely accepted procedure. TMTS prosthesis was introduced to Turkish Orthopedic Society as a cost effective solution in 1980s. The aim of our study is to report the results, complications and solutions of Turkish Musculoskeletal Tumor Society (TMTS) prosthesis at author's institute.
*Material and method:* Between 1991 and 2002, 42 patients who underwent wide resection of large bone segments because of primary malign and aggressive benign bone tumors on lower limb are studied. Distributions of the patients according to anatomic locations are as follows, 26 distal femur (62.2%), eight proximal tibia (17.7%), five proximal femur (13.3%) and three total femur (6.6%). Thirty male and 12 female patients with the average age of the patients was 28 (15–38) evaluated. Pathological diagnosis were 39 primary malign (32 osteosarcoma, three chondrosarcoma, two Ewing's tumor, one malign fibrous histiocytoma, one lenfoma of the bone,) three benign aggressive primary bone tumors. Three patients who had two distal and one proximal femoral resections reconstructed with massive allograft-prosthesis composite.
*Results:* In this study 15 patients had had custom-made prosthesis (35%) and the remaining 27 patients had had modular endoprosthetic prosthesis (65%). Complications were aseptic loosening in nine patients (21.4%), deep infection three (7.1%) recurrence three (7.1%), implant failure one (2.3%). Due to complications we performed 10 revisions, two amputations and two permanent removing of the prosthesis and thereafter knee arthrodesis with Ilizarov's distraction osteogenesis.
*Discussion:* Endoprosthetic reconstructions in appropriately selected patients can enhance the quality of the patient's life. Long-term oncological success contributed to high rate failure of the implant and subsequent revision. The complications that occur can be satisfactorily managed medically and surgically. Reoperations for prosthetic reasons are still promising, but reoperations for tumor recurrence or infection resulted in amputation in most cases. Although Ilizarov's method has some problems, this method may have salvaged the limbs when prosthesis permanently removed in conditions of progressive bone loss or infections. Our results are intermediate in follow-up especially in modular prosthesis, so the patients and their reconstructions will continue to be observed.

### Reconstruction of Defects following Bone Tumor Resections by Distraction Osteogenesis using Bone Transport

K. Erler, Mustafa Basbozkurt, Bahtiyar Demiralp, M.Taner Ozdemir, Ethem Gur
*(Department of Orthopedics and Traumatology, Gülhane Military Medical Academy, Etlik, Ankara 06018, Turkey)*

*Aim:* Reconstruction of bone defects following en bloc resection of malign or aggressive benign bone tumors is one of the major problems in Orthopedic Surgery. Objectives of an ideal reconstruction are biological recovery, adequate biomechanic stability and treatment with minimum complications. Distraction osteogenesis is a biological approach for repairing segmental bone defects. The aim of this study is to report our experience in the reconstruction of bony defects following en bloc resection of bone tumors by Ilizarovs' distraction osteogenesis using bone transport method.
*Material and method:* We performed en bloc resection in nine patients with bone tumors between October 1991 and January 2000. Two patients were female and seven were male. The average age of the patients was 19.3 (14–42). Histological diagnosis was osteosarcoma in four cases, Ewing's sarcoma in two, giant cell tumor (aggressive, stage 3) in one, osteofibrous dysplasia (active, stage 2) in one and osteoblastoma (aggressive, stage 3) in one. The epiphysis was preserved in all patients. Uniplanar external fixator was applied to one case and circular external fixator to other eight cases.
*Results:* The average follow-up period was 52.1 months (20–122). External fixator was removed in an average of 18.1 months (4–19) when sufficient bone consolidation was observed radiologically. Average bone defect after resection was 14 cm (8–24) and, average external fixation index, distraction index, and maturation index were 31.5 (18.7–40.0), 11.2 (10.9–11.2) and 17.8 (7.5–32.7), respectively. We observed healing in only two cases (cases 1 and 6) without major complication. We did not observe any early consolidation or osseous binding in the defect area in any patient.
*Discussion:* Several methods were reported for the restoration of bone defects following tumor resections. In the field of bone tumors, despite its limitations, the use of external fixators is varied. One of these uses is intercalar bone transport method described by Ilizarov. There is limited number of studies about the reconstruction of bony defects following en bloc resection of bone tumors with Ilizarovs' distraction osteogenesis using bone transport. It can be said that various results can be seen we observed some significant differences of using ECF in limb salvage after tumor resection compared to other uses. We believe that, despite high complication rate, Ilizarov bone transport technique is an alternative method for the restoration of bone defects formed following en bloc resection in the treatment of bone tumors.

### Interstitial Brachytherapy in Soft-Tissue Sarcomas: Experience with 31 Patients

N. Meushar, Rosenblat Edward, Kouten Abraham, Soudry Michael
*(Orthopaedic Oncology Service, Dept. Orthop. Surg. A and Brachytherapy Unit, Rambam Medical Center, Haifa 31096, Israel)*

*Introduction:* The present study reviews the experience of the Rambam Medical Center with interstitial brachytherapy in the treatment of 31 patients with soft-tissue sarcomas.
*Patients and Methods:* Thirty-one patients with variously located soft-tissue sarcomas were managed with a combination of wide local excision, brachytherapy of the tumor bed and external beam radiotherapy. In nine patients brachytherapy was the sole modality of post-operative treatment. In 26/31 patients, brachytherapy catheters were placed intra-operatively following radical surgery while in 5/31 patients The implant was performed as a separate post-operative procedure. Twenty-seven patients received LDR brachytherapy with Ir-192 seeds. Four patients received fractionated HDR brachytherapy using the micro-Selectron machine.
*Results and discussion:* With a median follow up of 36 months, the overall local control rate was 87%. Four of 31 patients (13%) failed locally at the implant site and 5/31 patients (17%) developed lung metastasis. Two of five patients with lung metastasis had a local recurrence as well. At the time of analysis, seven patients died of sarcoma (disease-specific mortality rate was 22.5%) while three died of other causes. The 5-year actuarial disease-free survival rate was 56% while the 5-year actuarial overall survival was 70%. Five patients (17%) developed severe wound complications following surgery/brachytherapy and six (19%) developed late local toxicity (fibrosis and telangiectasia).
*Conclusions:* Wide local excision followed by interstitial brachytherapy has resulted in an 87% local control rate with a 17% local complication rate.

### Spinal Cord Compression (SpCC) in Patients with Soft Tissue Sarcoma (STS)

O. Merimsky, Yehuda Kollender, Felix Bokstein, Josephine Issakov, Gideon Flusser, Isaac Meller, Moshe Inbar, Jacob Bickels
*(Unit of Bone and Soft Tissue Oncology, Department of Oncology, the National Unit of Orthopedic Oncology, Departments of Pathology,*

*Radiology, The Tel-Aviv Sourasky Medical Center, affiliated with the Sackler School of Medicine, Tel-Aviv University, Tel-Aviv, Israel, Tel-Aviv University, Tel-Aviv, Israel)*

*Introduction:* Spinal cord compression (SpCC) from metastatic cancer is a devastating event in the course of many cancers. It is associated with considerable morbidity and possible permanent disability if not alleviated by urgent treatment. In the present series, we report our experience in treating SpCC associated either with spinal metastases of soft tissue sarcomas.
*Patients and Methods:* The medical files of 19 patients (f = 9, m = 10) with high-grade (HG) soft tissue sarcoma (STS) who have developed SpCC were retrospectively reviewed. Age ranged between 28 and 76 years (median 50). A limb was the primary site of STS in 12 patients, the retroperitoneum in two, paraspinal tissues in two, groin or axilla in two, and unknown primary site in one. Six patients had liposarcoma, five had MPNST, four MFH, three synovial sarcoma, three leiomyosarcoma, one hemangiopericytoma, one extraskeletal chondrosarcoma, and one myogenic sarcoma. The disease stage (AJCC 1997) was T2bG3 in 11 patients, T1bG3 in two and stage IV in six. Systemic neoadjuvant or palliative chemotherapy or preoperative regional therapy has been administered in 13 patients before the development of SpCC.
*Results:* The levels of SpCC were the C spine in three patients, T spine in nine, L spine in six, and S spine in five. The prevailing symptom was pain. Impairment of sensation was found in 78% of SpCC events, while motor dysfunction in 48%, and sphincteric problems in 13%. The KPS ranged between 40 and 70% at presentation. Treatments included surgical decompression in three patients, and radiation therapy in all the patients. Small but important improvement of signs and KPS was noted in 14/23 events, expressed mainly by pain alleviation and restoration of independence. The median time interval between the diagnosis of STS to the development of the SpCC was 28 months. The median time interval between the occurrence of the first metastatic event to the presentation of SpCC was 3 months. The median survival following the diagnosis of SpCC was 5 months.
*Conclusion:* SpCC is not uncommon event in patients with sarcoma. It represents a relatively late event in their disease course. Team approach is mandatory for diagnosis and treatment.

### Resection-Curettage and Cryosurgery for Low-Grade Chondrosarcomas of the Extremities. Surgical Indications and Result with an Average Follow-Up of 6 Years

J. Bickels, Felasfa Wodajo, Yehuda Kollender, James Wittig, Kari Mansour, Martin Malawer, Isaac Meller
*(The National Unit of Orthopedic Oncology, Tel-Aviv Sourasky Medical Center, 6 Weizmann Street, Tel-Aviv 64239, Israel)*

*Introduction:* We have utilized cryosurgery as an adjunct to cryosurgery to eradicate residual microscopic disease in an effort to improve local tumor control over curettage alone in the management of low-grade chondrosarcomas of the extremities.
*Materials and methods:* Patients were considered preoperatively as having a low-grade chondrosarcoma if they presented radiological evidence of endosteal scalloping and/or cortical destruction and breakthrough, associated with deep and constant pain. There were 46 tumors, of which 23 were located at the proximal humerus, 16 in the distal femur, four in the proximal tibia, and three around the hand and wrist. All surgeries were performed by similarly trained surgeons at two institutions and consisted of resection-curettage through a wide cortical window, followed by high-speed mechanical burring and supplemented by one to two freeze cycles using liquid nitrogen. Defects were reconstructed using a combination of polymethylmethacrylate and internal fixation. Follow-up ranged from 25 to 162 months (mean, 73 months) and functional evaluation according to the American Musculoskeletal Tumor Society System.
*Results:* At the most recent follow-up, none of the patients had local tumor recurrence or distant metastases. There were no deep wound infections, neurovascular, or thromboembolic complications. Functional results in the proximal humerus were as follows: excellent, six; good, 14; and fair, three. Functional results in the distal femur were: excellent, two; good, 13; and fair, one. One nondisplaced unicortical fracture in the distal femur was treated successfully without immobilization. A second 70-year-old patient developed postoperative degenerative changes of the shoulder joint which necessitated shoulder arthroplasty.
*Conclusions:* Resection-curettage for low-grade chondrosarcomas of the extremities allows for excellent preservation of function with minimal rates of complications and local tumor recurrence. Therefore, we do not recommend en-bloc resection as a surgical guideline in the management of low-grade extremity chondrosarcoma.

### Distal Femur Resection with Endoprosthetic Reconstruction. A Long Term Follow-Up Study

J. Bickels, Yehuda Kollender, James Wittig, Robert Henshaw, Robert Neff, Martin Malawer, Issac Meller
*(The National Unit of Orthopedic Oncology, Tel-Aviv Sourasky Medical Center, 6 Weizmann Street, Tel-Aviv 64239, Israel)*

*Introduction:* The distal femur is a common site for primary and metastatic bone tumors. It is, therefore, a frequent site in which to perform a limb-sparing surgery. The authors describe their experience with distal femur resection and endoprosthetic reconstruction.
*Methods:* There were 61 males and 49 females who ranged in age from 10 to 80 years. Nineteen patients were younger than 12 years of age. Diagnoses included 99 malignant tumors of bone, nine benign-aggressive lesions, and two non-neoplastic conditions that had caused massive bone loss or articular surface destruction. Endoprosthetic reconstruction included 73 modular, 27 custom-made, and 10 expandable prostheses. Only eight patients had a constrained knee mechanism; the remaining patients underwent reconstruction with a rotating-hinge knee mechanism. All prostheses were fixed with bone cement. Soft-tissue reconstruction included 21 medial, three lateral, and one bilateral gastrocnemius flaps. All patients were followed for more than 2 years (range, 2–16.5 years; average, 5.2 years); Follow-up included physical and radiological evaluation and functional evaluation according to the American Musculoskeletal Tumor Society System.
*Results:* Function was estimated to be good or excellent in 94 patients (85.4%), moderate in nine patients (8.2%), and poor in seven patients (6.4%). Patients who underwent reconstruction with a rotating-hinge knee mechanism were more likely to have a good-to-excellent functional outcome (91%) than those who underwent reconstruction with a constrained knee mechanism (50%). Complications included six deep wound infections (5.4%), which resulted in three amputations, two prosthetic revisions, and one wound debridement. There were six local recurrences, five of which were treated with wide local excision, and one necessitated amputation. Overall, there were 14 revision surgeries; these included replacement of failed polyethylene component in six patients and prosthetic revision in eight patients (aseptic loosening, five; deep infection, two; and radiation bone necrosis. one). There were six local recurrences, five of which were treated with wide local excision, and one necessitated amputation. Prosthetic survival was 94% at 5 years and 91% at 10 years and overall limb salvage rate was, therefore, 96%.
*Conclusions:* Distal femur resection with endoprosthetic reconstruction is a safe and reliable procedure, which provides good local tumor control. The use of cemented endoprostheses, combined with a rotating-hinge knee mechanism provide immediate mechanical stability, allows early mobilization and good-to-excellent function in most patients. The use of endoprostheses is recommended by the authors for reconstruction of large segmental defects of the distal femur.

**Vacuum-Assisted Wound Closure following Resection of Large Musculoskeletal Tumors**

R. Gabay, Jacob Bickels, Yehuda Kollender, Alexander Nirkin, Carol Emold, Shula Lev, Benjamin Bender, Isaac Meller
*(The National Unit of Orthopedic Oncology, Tel-Aviv Sourasky Medical Center, 6 Weizmann Street, Tel-Aviv 64239, Israel)*

*Introduction:* Resection of large or recurrent musculoskeletal tumors and especially those that are located in a previously irradiated field may result in an extensive soft-tissue defect that can not be closed primarily. We present our experience with vacuum-assisted wound closure (VAC) in eight consecutive patients.
*Materials and Methods:* Between May 2002 and January 2003, eight patients were treated postoperatively with VAC after being left with an extensive and deep soft-tissue defect that could not be closed primarily. There were five males and three females who ranged in age from 15 to 70 years (median, 59.5 years) and had been diagnosed with five bone and three soft-tissue tumors. Anatomic locations were: pelvic girdle, four; lower extremities, two; groin, one; back, one. Six patients had more than one surgical intervention and/or radiation therapy to the treated site (two had one surgery, four had two surgeries, and two had four surgeries). Four of the eight patients were treated with radiation therapy. VAC consisted of application of a sponge over the surgical wound, covering the sponge with adhesive plastic dressing, and their connection via suction tubes to an electric pump that keeps a constant negative pressure of ·100 mmHg. The sponge is usually changed as a bedside procedure at 48-h interval.
*Results:* The treatment period lasted from 7 to 19 days (mean, 14.5 days, median 11.5 days). At the termination of VAC, wounds were left for secondary closure in four patients and primary closure with skin graft was carried out in the remaining four. None of the patients required a free muscle flap.
*Conclusions:* The use of VAC facilitates simple closure of extensive surgical wounds that otherwise would require a long period of healing, complex muscle flaps, or an amputation. In some patients, VAC may even avoid the need for surgical wound closure. The procedure is simple, safe, and cost-effective. We recommend VAC for the management of large soft-tissue defects remaining after resection of musculoskeletal tumors.

**Surgical and Reconstructive Procedures in Children with Osteosarcoma. The Scandinavian Sarcoma Group Experience**

Otte Brosjö, Henrik Bauer
*(Department Of Orthopedics, Karolinska Hospital, Stockholm 171 76, Sweden)*

In this study, the choice of surgical procedure and preferred reconstruction in all children with high-grade osteosarcoma (OS), was retrospectively analyzed.
*Patients and methods:* Since 1986, the surgical treatment of 152 children under the age of 16 at diagnose of OS, have been reported to the Central Registry of the Scandinavian Sarcoma Group (SSG). OS of the axial skeleton was excluded. All children received neoadjuvant chemotherapy according to the specific SSG-protocol running at the time of diagnose.
*Results:* An amputation was performed on 56 children. However, the majority (52) was performed during 1986–96. Only four children have been amputated since 1997. Local excision was performed in 96 cases. The most common reconstructive procedure in 49 distal femur OS was megaprostheses (26) followed by rotationplasty in 14 children. A few osteochondral allograft reconstructions (six) have been performed in distal femur, only one since 1995. Reconstruction of proximal tibia is mainly with megaprosthese or a massive allograft, whereas the most common reconstruction of proximal humerus is megaprostheses or vascularized fibulagraft.
*Conclusions:* This retrospective, multicenter study shows that a child with OS of the extremity now have a very low risk of having an amputation. Megaprosthetic replacement is the most common reconstructive procedure after local excision of an OS in distal femur and proximal tibia/humerus. Rotationplasty seems to be rather popular in children with OS in distal femur.

**Chondrosarcoma of the Thoracic Spine: a Case Report**

Benjamin Bender, Moshe Salai, Nissim Ohana
*(The Spine Surgery Unit, Rabin Medical Center, Beilinson Campus, Petach-Tikva 49100, Israel)*

Chondrosarcoma of the spine is a rare entity, Chondrosarcomas of the spinal column are most frequently found in the thoracic spine, the treatment of chondrosarcoma is surgical excision. Total excision may be difficult due to the anatomy of the area. We describe a case of 40-year-old female with complaints of back pain and radiological evidence of a lytic lesion in a collapsed T8 vertebra with soft tissue component and extension of the lesion to the adjacent vertebra. On CT guided core needle biopsy many giant cells were found, it was suggested that the findings may represent a Giant cell tumor. CT guided phenol ablation was then performed; no change in tumor size was noted. The information acquired by means of radiography and cytology influenced the decision making of the type and extension of the appropriate surgery. The location and extension of the tumor in the thoracic spine mandated an extensive removal of the tumor as far as possible to minimize the chances of local recurrence which correlates highly with poor clinical come. Restoration of spinal column stability and minimization of neural deficits compelled towards anterior and posterior approach. Surgery consisted of three stages. The First stage was in posterior approach, Bilateral costotransvesectomy T7-8-9-10 was done, then wide laminectomy T7-8-9, and posterior spinal fusion from T5-6 to T10-11 were performed. The Second stage was in anterior approach, right thoracotomy was performed and en bloc resection of T 7-8-9 with preservation of the vessels and cord and anterior spinal fusion of T6-10 was conducted. The Third stage included kyphoplasty and compression of the constraction and bone grafting. The duration of operation was long, approximately 15 h. The patient was ambulatory few days after surgery, and no neurological deficiency was noted. The histology revealed I grade chondrosarcoma with secondary aneurysmal bone cyst. The patient is currently one year after surgery walking freely and there is no evidence of recurrence. This case illustrates the combination of improved resection, stabilization, and fusion techniques that allow for more aggressive removal of malignant spinal tumors with acceptable mortality and morbidity.

**The use of Synthetic Bone Substitutes in the Treatment of Bone Defects Following Resection of Benign Bone Lesions and Conditions**

Benjamin Bender, Jacob Bickels, Rosi Brand, Isaac Meller, Alexander Nirkin, Yehuda Kollender
*(The National Unit of Orthopedic Oncology, Tel-Aviv Sourasky Medical Center, 6 Weizman St., Tel-Aviv 64239, Israel)*

*Introduction:* We describe our experience with synthetic bone substitutes (hydroxyapatite (HA), tricalcium phosphate (TCP) and calcium sulphate) in feeling bone defects following resection of benign bone lesions and bone conditions. Bone grafting is frequently used to augment bone healing. Autologous cancellous bone graft is considered the most effective grafting material. Complications associated with autologous bone grafting and limitations in the quantity of bone available led to the wide use of alternative bone substitutes. Alternatively allografts have been reported to have a significant incidence of postoperative infection and fracture as well as the potential risk of disease transmission. A variety of synthetic bone graft substitutes have been developed with the aim to minimize these complications. The benefits of synthetic grafts include availability, sterility and reduced morbidity. Hydroxyapatite, tricalcium phosphate and calcium sulphate act as a filter or expander and they have osteointegrative and conductive properties.

*Materials and methods:* Between January 1998 and October 2000, 82 patients with benign bone lesions underwent intralesional resection. The bone graft substitute was not used to provide structural continuity of bone. Tricalcalcium phosphate(Biosorb) was used to fill the lesional cavity in 44 patients, in 34 patients hydroxyapetite (Proosteon, Cementk) was used, and 10 patients were treated with calcium sulfate (Osteoset). Two types of synthetic bone substitute were used simultaneously in six patients, three patient were treated with TCP and calcium sulfate, two patients with calcium sulfate and HA, and one patient with TCP and HA. Bone defects were primarily located in the long bones (86.5%), with the femur and tibia alone accounting for about 75.6% of the cases, the fibula, humerus and ulna accounted for additional 10.9% of the cases. There were 53 male and 29 female patients who ranged in age from 7 to 68 years (mean 22.5 years). Thirty-eight patients had benign bone tumors, 31 patients had benign-aggressive bone tumors, 12 patients had osteomyelitis and one patient had melorheostosis. Thirty-eight patients (46.3%) in addition to intralesional resection underwent cryosurgery treatment. Bone substitutes were used in combination with autologous cancellous bone graft in 16 patients (19.5%), and in combination with vascularized free fibular graft in seven patients (8.5%). Thirty-five patients (42.68%) with bone defects that compromised the overall stability of the affected bone underwent additional reconstruction by internal fixation, PMMA was further used in 25 patients of this group. After surgery partial weight bearing was allowed after 4 weeks, following cryosurgery partial weight bearing was allowed after 6 weeks. During that time, rehabilitation emphasized adjacent joint active and passive motion. For the first 2 years after surgery, patients were evaluated every 3 months. On each visit physical examination was conducted and radiographic picture was obtained. For the additional time evaluation was conducted every 6 months thereafter. The degree of bone substitute incorporation and bone healing were determined radiographically. Functional outcome was prepared according to the American Musculoskeletal Tumor Society System.

*Results:* All patients were followed up clinically and radiographically and evaluated until bone defects were healed or for a minimum of 2 years (mean 31.5 months). All patients were ambulatory. 77 out of 82 (93.9%) bone defects healed, full incorporation of the bone substitutes into the bone was observed in these patients. Functional outcome was estimated to be good to excellent in 68 patients (82.92%), fair in nine patients (10.97%) and poor in five patients (6.09%). Poor functional outcome in three patients was attributed to the close location of the bone lesion to the articular joint surface and in one patient to deep wound infection. One patient with a lesion located in the femoral head developed avascular necrosis (AVN) after the primary operation, he underwent free fibular vascularized bone grafting and the functional outcome was estimated as good following the second procedure. Roentgen imaging correlated greatly with the functional outcome. Three patients (3.65%) had deep wound infection, all the patients with the deep wound infection were reoperated and debridement of the surgical wound was carried out, intravenous antibiotic treatment followed the surgical treatment, the patient's functional outcome was good in one patient and poor in two patients. Four out of six (66.6%) patients who were treated simultaneously with two types of synthetic bone substitute developed superficial wound infection. Overall seven patients (8.53%) had superficial wound infection, all were treated successfully with oral antibiotics. Six patients (7.31%) had fractures at the site of the operation, three fractures were secondary to a local trauma, four patients with crack fractures were treated conservatively by means of non weight bearing for several weeks, one patient was treated with a cast, and one patient underwent internal fixation, all had good functional outcome. Two patients (2.43%) had superficial skin necrosis healed with conservative local care; the skin necrosis was ascribed to cryosurgery. Four patients (4.87%) had persistent low grade fever, no evident source for the fever was found, and it was ascribed to the bone substitutes.

*Conclusion:* Synthetic bone substitutes TCP, HA and calcium sulphate are safe, readily available and easy to use during surgery. Bone substitutes are reliable and effective; it provides constructive scaffolding for the formation of new bone and is gradually incorporated into the bone. Bone substitutes can reduce the morbidity associated with the additional surgery required to obtain autologous bone graft, and it can be used in combination with autologous bone graft or as an alternative to it.

**Expression of Novel Surface Markers in Chondrosarcoma**

Pancras Hogendoorn, P. Mainil-Varlet, J.V.M.G. Bovée, M. Zheng, J. Diaz Romero, E. Stauffer, F. Auf der Maur, J.O. Gebbers

*(Departments of Pathology, Leiden, Bern, Luzern University; Leiden 2311 XH, P.O. Box 9600, The Netherlands)*

*Background:* To explore the phenotypic differentiation pathways in normal and neoplastic cartilage, a study was designed focussing on the expression of cell surface molecules. We have previously demonstrated a dramatic change in the expression of surface markers in chondrocytes by expansion in monolayer culture. Interestingly, the morphological transition from a non-proliferative rounded phenotype (primary, freshly isolated) to a proliferative fibroblastic phenotype is also associated with the expression of several surface markers characteristic of mesenchymal stem cells.

*Materials and methods:* Isolated normal chondrocytes were analysed by fluorescence activated cell sorting (FACS) using a panel of 63 CDs antibodies. In a further experiment the chondrocytes were seeded on culture flasks, allowed to dedifferentiate into the typical fibroblastic phenotype and passaged for up to 2 weeks. To characterise chondrosarcoma via FACS using the panel of CDs employed in our chondrocyte phenotype studies, a grade II chondrosarcoma was digested enzymatically as described before. CD31 was used to exclude that the results of FACS resulted from endothelial cells. Differences in expression level were verified by immunohistochemistry and RT-PCR.

*Results:* Isolated normal chondrocytes revealed expression of: Integrins: CD49a, CD49b, CD49c, CD 49e, CD49f, CD51/CD61; Adhesion molecules: CD44, CD54, CD106, CD166; Tetraspanins: CD9, CD63, CD81, CD82, CD151; Cytokine receptors: CD105, CD119, CD130, CD140, CD221. In contrast, chondrosarcoma cells displayed a higher expression of CD49f, CD49d, CD 58, CD81, CD221. No change was detected in the expression of CD49e, CD44, CD 106 or CD9. Curiously, we did not detect the expression of CD140a (PDGF-$\alpha$ Receptor) on chondrosarcoma cells, which is normally observed in expanded normal chondrocytes. Results were confirmed by RT-PCR. The PDGF-$\alpha$ receptor has been described to be overexpressed in grade III chondrosarcomas. Interestingly, further immunohistochemical analysis of CD44 demonstrated expression in enchondromas, and grade I and II chondrosarcomas, while grade III

chondrosarcomas and metastases demonstrated absence of this adhesion molecule.
*Conclusion:* Our preliminary results confirm the presence of unexpected CD markers on the chondrocytes and the modulation of their expression in tumorous conditions.

### Osteosarcoma Relapsing With Skeletal Metastases

Stefan S. Bielack, Beate Kempf-Bielack, Silke Flege, Heribert Jürgens
*(Cooperative Osteosarcoma Study Group (COSS), Department of Pediatric Hematology and Oncology, University Children's Hospital Münster, Münster 48149, Germany)*

After the lungs, the skeleton is the organ system most frequently involved by recurrent osteosarcoma. We report 90 osteosarcoma patients, treated on neoadjuvant COSS studies and registered prior to June 1998, who presented with distant skeletal involvement at first relapse. These represent 15.6% of all relapses occurring in the cohort. The median interval from initial diagnosis to diagnosis of bony relapse was 1.6 years (range: 0.14–14.3). Forty-five relapses were limited to the skeleton and 31 of these involved a solitary bone lesion only. The other 45 relapses additionally involved the lung (40), local recurrence (four), and/or other sites (seven). Treatment for relapse included surgery in 47 patients (second complete remission: 30), radiotherapy in 20, and chemotherapy in 65. After median follow-up periods of 1.0 year (range: 0.02–17.8) for all patients and 2.6 years (range: 0.3–17.8) for 19 survivors, only nine patients remained in continuous second remission. Sixteen of 31 patients with a solitary bone lesion (eight in second CR), but only 3/59 other patients (only one in second CR) were alive. The 5-year survival probability after a first relapse involving distant bone was 12% (SE 4%) for all 90 patients, 39% (SE 11%) for 31 patients with a solitary bone lesion only, and 59% (SE 14%) for those 18 among the 31 who achieved a second surgical remission. In multivariate analysis, solitary bone lesions and the use of surgery and chemotherapy were associated with a better outcome. When corrected for surgical remission status, radiotherapy was also found beneficial. In summary, metachronous skeletal involvement by osteosarcoma carries a poor prognosis. Solitary lesions may be cured by a treatment strategy including complete resection. It may be speculated whether these solitary lesions represent true metastases or second primary osteosarcomas.

### Innovation In Limb Sparing Surgery

Chris Henry
*(The Bone Tumour Unit, Royal National Orthopaedic Hospital, Brockley Hill, Stanmore, Middlesex HA7 4LP, England)*

The first non-invasive extendable endoprosthesis has been successfully used in limb sparing surgery for a 12-year-old girl with osteosarcoma in her (L) distal femur. Previously, the extendable prostheses used in the UK required lengthening under a general anaesthetic, using an Allen key to engage the worm screw mechanism. This procedure carries risks, not only from an anaesthetic point of view, but also from a surgical perspective, in relation to infection and potential loss of function. In appropriate patients, the new non-invasive extendable prosthesis protects young patients from these risks, reduces hospitalisation and prevents the physical and psychological trauma of further surgery. The limb may be lengthened at a slower rate, equating with normal growth, in the outpatient department. The procedure is painless.

Presentation will include: (1) a short history of extendable endoprostheses in the UK; (2) the principles of the non-invasive extendable prosthesis; (3) a nursing and patient perspective.

### The Evolution of the Nurse Consultant in Orthopaedic Oncology

Lin Russell
*(Royal Orthopaedic Hospital NHS Trust, Bristol Road South, Nothfield, Birmingham B31 2AP, England)*

*Aim:* The development of new nursing roles engenders the concept of ensuring the most experienced and educated practitioners are retained within clinical roles to facilitate clinical excellence. In response to the concerns and recommendations the Government announced the introduction of Nurse Consultant posts in the NHS in 2000. In light of the Government change in NHS strategy away from waiting list initiatives towards improvement in cancer care and coronary heart disease, Saving Lives (1999) places the emphasis on quality of life and not purely quantity of life years.
*Aims and objectives of the post:* To provide better outcomes for patients by improving services and quality. The nurse consultant in orthopaedic oncology fulfils an expert practice function: Professional leader; Play a crucial role in Clinical Governance by promoting best practice through leadership and change management skills; Erodes both professional and organisational boundaries and so develop the total patient experience. Create a nurse consultancy service, which has the patient at its heart. Make professional and autonomous clinical decisions for which they have sole responsibility and accountability. A survey of patient satisfaction was carried out to compare established nurse-led clinics with clinics run by medical staff. Target population follow-up patients who had undergone limb salvage with endoprosthetic replacements.
*Methodology:* Quantitative study, experimental design control group the medical staff experimental group nurses with the dependent variable being patient satisfaction.
*Results:* demographic data was broadly comparable in both groups with overall satisfaction high.
*Conclusion:* In each category nurses and doctors were comparable, however, nurses scored higher in the psychological and empathetic areas of care.

### Who is Missing Soft Tissue Sarcomas?

Andrea Hughes, L. Russell, R. Grimer
*(Royal Orthopaedic Hospital Oncology Service, Birmingham B31 2AP, England)*

*Aim:* To determine, through patient mapping, how long it takes for a patient to be referred to the oncology service once they have noticed a lump.
*Method:* Patients who presented to the oncology service with suspected soft tissue sarcomas were asked to complete a questionnaire with the guidance of the cancer services co-ordinator. The questionnaire focused only on the patient journey from start of symptoms to specialist referral. It included each visit to the general practitioner and general orthopaedic surgeon and the investigations done at each visit.
*Results:* There is a huge variance in patient experience. The most common initial symptom was a lump that was increasing in size but there were also reports of pain and swelling. Many patients have at least two visits to their GP prior to any other referral being made and then at least one visit to a general orthopaedic surgeon prior to referral to the oncology service.

*Conclusion:* There is a need for further education for general practitioners in order to improve the patient experience of soft tissue sarcoma.

**Immunohistochemistry of Desmoid Tumors**

Andreas Leithner, Gapp Markus, Windhager Reinhard, Beham Alfred
*(Department of Orthop. Surgery, Karl Franzens University, Graz A-8036, Austria)*

*Background and Objectives:* The optimal treatment of aggressive fibromatoses (desmoid tumors) is unclear, often being based on sporadic immunohistochemical reports with a low number of cases. Therefore, a large immunohistochemical study was planned, in order to provide a theoretical basis for adjuvant therapy regimes.
*Methods:* Eighty-one tissue samples from 60 patients (37 female, 23 male) with desmoid tumors (24 extra-abdominal, eight intra-abdominal, 21 abdominal, seven infantile) and a mean age of 35 years (range, 1–83) were tested for estrogen, progesterone, and androgen receptors, and somatostatin, as well as HER2, Cathepsin D, and Ki-67.
*Results:* In all cases HER2 and estrogen receptors were negative, staining for progesterone receptor was positive in one abdominal case, positive staining for androgen receptor was found in 15 of 21 abdominal and seven of 24 extra-abdominal cases, staining for somatostatin was positive in five of 24 extra-abdominal and one of seven infantile fibromatoses, staining for Cathepsin D was positive in all cases, a positive Ki-67 staining was found in two of eight intra-abdominal, six of 21 abdominal cases, nine of 24 extra-abdominal and five of seven infantile aggressive fibromatoses.
*Conclusions:* The data of this immunohistochemical study prove that the published effect of anti-estrogens in the treatment of aggressive fibromatoses is not to be attributed to an estrogen receptor expression. Furthermore, a correlation between the clinical behavior of desmoid tumors and the immunoreactivity to androgen receptor and Ki-67 could lead to a better understanding and probably more specific treatment of this tumor.

**Local Wide Excision and Brachytherapy in the Treatment of Recurrent Soft Tissue Tumors**

Murat Hiz, Rustu Eklioglu, Fazilet Dincbas, Sedat Koca, Sergulen Dervisoglu
*(Department of Orthopaedics, Cerrahpasa Medical Faculty, Istanbul University, Istanbul 34303, Turkey)*

Local excision of recurrent soft tissue sarcomas or aggressive soft tissue tumors is possible by surgical means but subsequent local recurrence rate is high due to marginal borders in some areas such as popliteal space, axilla, foot and groin or difficulties of defining the extent of recurrent tumor at the former operation site. Local control of recurrences by marginal excision only or with subsequent external irradiation or systemic chemotherapy is not sufficient regarding the decrease of recurrence rate if the tumor had recurred more than once or recurrence occurred despite postoperative irradiation. Brachytherapy might be and effective adjuvant after excision of tumors that recurred more than two times. Eight patients, six males, two females, with mean age 34 (11–71), with the diagnosis of repeatedly recurrent tumors, six desmoid fibroma, one liposarcoma, one synovial sarcoma were treated by above-mentioned technique between November 2000 and June 2002. Six of these patients had refused amputation offer. Mean follow up was 16.5 months (9–27). Six tumors had recurred two times, two tumors had recurred three times. Four of these patients had received post operative external irradiation after removal of first recurrence but the tumor had recurred again and one patient had received preoperative irradiation before removal of second recurrence (three desmoid fibroma, one liposarcoma, one sinovial sarcoma). Eight patients had 1200–4600 cGy (mean 3750 cGy) single plane interstitial brachytherapy in four to 23 fractions (mean 16) subsequent to four marginal four wide excisions. Brachytherapy was used for applying a booster dose in the patient who received preoperative irradiation. Regarding complications; two patients with desmoid fibroma recurred. Two patients with former external irradiation developed deep skin necrosis requiring debridement and subsequently developed infection. Infection was treated by biological wound dressing and systemic antibiotics. Secondary wound healing was obtained at 3 months time. Two patients showed superficial wound healing problems not effecting final outcome. Two patients with desmoid fibroma developed new tumor formation at a more proximal site of brachytherapy area (1–2 cm s) and treated by local wide excision. Four patients have no recurrence yet. None of the patients required amputation. Regarding functional status all patients with tumors in the lower extremity were able to walk independently; two patients with the tumors in the axilla and elbow had moderate restriction of range of motion. Psychological acceptence of the salvaged extremity with the sarcoma beyond local control with conventional treatment was extremely good. Brachytherapy was proved to be an effective adjuvant therapy after consecutively recurrent soft tissue tumors with reasonable complication rate, in our hands.

**Surgical Interventions for Thoracic and Lumbar Spinal Metastases: Survival, Complications and Functional Outcome in 277 Consecutive Patients**

Henrik C.F. Bauer, Karl-Åke Jansson
*(Department of Orthopedics, Karolinska Hospital, SE-171 76 Stockholm, Sweden)*

*Introduction:* The aim in treating spinal metastases is palliation, i.e., to relieve pain and restore neurological function. In this series of patients with spinal metastases, the main indication for surgery was epidural compression with neurological deficit. Only a few patients were operated for pain without motor deficit.
*Patients and methods:* A total of 277 patients, 69% men and 31% women, mean age 66 (23–93) years, were operated for spinal metastases in 1992–2001. The most common site of primary tumor was prostate in 40%, breast in 15%, kidney in 8%, and lung in 7%. Pathological fracture was seen in 55% and the site of epidural compression was thoracic in 78%. Preoperatively assessment of the neurological function according to Frankel showed that 62% were non-walkers (A 3%, B 9%, C 52%), and 38% could walk with our without support (D 30%, E 8%). Thirteen percent had a solitary skeletal metastases, 63% had numerous skeletal metastases and 24% non-skeletal metastases as well.
*Results:* Posterior approach dominated, 254 procedures. Among these laminectomy alone was performed in 17%. Osteosyntheses was based on hooks in 49%, pedicle screws in 21%, and mixed in 13%. Neither cementation nor bone grafts were applied. An anterior approach was only used in 23 cases, and reconstruction of the vertebral bodies was based on plates and polyacrylatic cement. The median operating time was 3 (0.5–8) hours and blood loss 1500 (100–16500) ml. Postoperative complications were 12% wound healing problems including infection, 5% systemic complications and 1% hardware problems. Ten percent of the patients died within 1 month of surgery. Only 47% survived 6 months, 30% 1 year, and 15% 2 years. Among the 252 patients with neurological deficit, 70% improved at least one Frankel grade postoperatively and 4% were worsened. At 1 year, 89% could walk (Frankel D and E), and at 2 years 85%.
*Conclusions:* Surgical treatment improved the neurological function in the majority of patients. However, the complication rate was high and it was difficult to identify patients with a very poor prognosis. The patients who survive had lasting restoration of function.

**Improved Local Control in Soft Tissue Sarcoma – Radiation and/or Surgical Margins**

Henrik C.F. Bauer, Otte Brosjö, Rikard Wedin, Jan-Olof Fernberg
*(Departments of Orthopedics and Oncology, Karolinska Hospital, SE-171 76 Stockholm, Sweden)*

We have reviewed how margins and the use of radiotherapy have changed over time and report on the resultant local recurrence rate. A total of 577 adult patients with soft tissue sarcoma of extremities or trunk wall were operated since 1986. Only patients without metastases at diagnosis and who were operated for primary tumor at the sarcoma center were included. For comparison we have chosen three time periods: early (1986–90), middle (1991–1995), and late (1996–2002). The overall local recurrence rate (Kaplan-Meier) was 0.20, and for the three time periods 0.32, 0.19, and 0.13. In the following, subcutaneous lesions ($n = 208$) were assessed separately from the deep ($n = 369$). The rate of wide margins for subcutaneous lesions was 77%, improving from 61% in early, to 81 and 79% in the middle and late periods; 15% had adjuvant radiotherapy and the rate has not changed. The 5-year local recurrence rates were 0.23, 0.18 and 0.13 for the three time periods. The amputation rate for deep lesions was 10% (7, 15 and 9%). The overall rate of wide margins was 55% and did not change during the time period (53, 62 and 53%). However, the use of radiotherapy almost doubled in the late period, from 30 to 57%. Furthermore, radiotherapy was given as preoperative treatment in 52% of the patients treated late, as compared to 6% previously. Only 8% had adjuvant chemotherapy overall, 13% in the late period. The percentage of patients who had a poor surgical margin and no radiotherapy steadily decreased (20, 16 and 11%). The 5-year local recurrence rate decreased from 0.35 early, to 0.20 middle, and to 0.13 late. These results show the most striking improvement in local control occurred between the early and middle period without increasing the use of radiotherapy but improving the surgical margins. With doubling of the use of radiotherapy for deep lesions, local control improved further but only marginally.

**Limb-Saving Surgery for Malignant Musculoskeletal Tumours in Children**

Alexander Shchurovsky
*(Romanyshyn Bohdan, Dnistrovska Str. 27, Lviv 79035, Ukraine)*

*Introduction:* Different methods of limb-saving surgery are widely used in the reconstruction after tumour resection, including osteo-articular allograft, prosthetic reconstruction, allograft-prosthetic composite reconstruction and arthrodesis. The longevity, complications and functional results of these methods depends from anatomic location, age and type of surgery.
*Materials and methods:* Between January 1993 and January 2003, 68 patients with malignant musculoskeletal tumours were treated. Approximately 60% of patients were admitted in late stage of disease and only 35 of them (24 males and 111 females) underwent limb-saving surgeries. The age ranged from 1 to 18 years (mean, 14.8 years). The diagnosis were: osteosarcoma, 15 patients; Ewing's sarcoma, seven patients; malignant giant cell tumours, five patients; soft-tissue sarcomas, five patients; chondrosarcoma, two patientsl; and malignant fibrous histiocytoma, one patient. The tumour locations were: proximal femur, one patient; distal femur, 12 patients; proximal tibia, two patients; proximal fibula, four patients; distal fibula, two patients; humerus, five patients; ribs, three patients; forearm, two patients; foot, two patients; pelvis and hand in one case. For these patients, different types of limb-saving surgery were performed: prosthetic replacement, 13 patients; resection, 14 patients; resection with allo-, auto-, or ceramoplasty, seven patients; and rotationplasty in one case.
*Results:* The earlist follow-up for prosthetic replacement was 70 months; for resection–reconstruction, 47 months; and after resection, 89 months. The function was excellent and good in 12 patients after prosthetic replacement (92.3%); in five patients after resection–reconstruction (71.4%); and in 12 patients after resection (85.7%).
*Conclusions:* We achieved better functional results in patients after prosthetic replacement. This kind of treatment has faster and better effect in comparison with the other techniques.

**Ceramic Hydroxyapatite: Bone Defects Filling Material**

Alexander Shchurovsky
*(Romanyshyn Bohdan, Dnistrovska Str. 27, Lviv 79035, Ukraine)*

*Introduction:* The research of the materials for bone defects filling has a long history. In modern orthopaedics hydroxyapatite and tricalciumphosphate based materials are used most often for bone tissue substitution. This report informs about the utilisation of Ukrainian domestic material: ceramic hydroxyapatite (CERHAP).
*Materials and methods:* CERHAP is completely similar with the natural bone tissue. It features absolute biocompatibility and osteogenesis stimulation. CERHAP is used in a form of powder, granules, dense and porous ceramics. During March 1999 to January 2003 there were 53 patients aged from 4 to 18 years (mean 13.4 years). They underwent surgery with CERHAP. Most of the patients had benign bone tumours and tumour-like diseases: one a malignant giant cell tumour; one chronic osteomyelitis.
*Results:* Follow-up results of 1–46 months showed good effect and bone shape recover in all patients. No complications were noted.
*Conclusions:* Our positive results of CERHAP utilisation confirm its usage efficacy as a plastic material for bone defects filling.

**Spinal Cancer Metastases: Indications, Treatments and Results. Our Experience in 284 Localizations**

Alessandro Gasbarrini, R. Bevoni, S. Bandiera, L. Mirabile, G. Barbanti, F. De Iure, S. Terzi, E. Bertoldi, M. Fravisini, G.B. Scimeca and S. Boriani
*(Ospedale Maggiore, Largo Nigrisoli 2, Bologna 40100, Italy)*

From 1996 to 2002 we treated 284 localizations of spinal metastases in 168 patients (108 males and 60 female). We studied preoperatively all the patients with standard X-rays and scintigraphy followed by CT scan or MRI. The average age at diagnosis was 56.6 years (range: 14–89). Surgical indications are: instability of the spine, pain, paresis, life expectancy and local control of the disease. We found 22 different isotypes of metastases; kidneys, lung and breast were the most common primary sites (61.2%). Treatment was: in 80 cases three-dimensional decompression of nervous structures, in 57 cases an intralesional aggressive curettage (debulking of the lesion), and in 19 cases it was possible to perform an en-bloc resection of the tumoral lesion. All cases were then treated with instrumental stabilizzation. Thirty-seven cases (24%) were treated in emergency. The poor general condition of the patient, the aggressiveness of the tumoral growth, the sensitivity of the metastases to adjuvant therapy or the absent risk of pathological fractures prompted us to proceed with a non-surgical approach of the metastases in 12 cases. Survival depended on anatomic site of primary carcinoma, preoperative neurological deficit, extent of disease, number of vertebral bodies involved, tumoral location and age. Of all the patients treated, 104 died at an average of 12 months (range: 1–50) after surgery, and 41 patients are still alive with an average follow-up of 21 months (range: 6–66).
*Conclusions:* Spinal metastases are only apparently similar lesions but they are really different considering the large varieties of isotypes and the spread of the primary tumor. These metastases develop early and are not terminal events, they have to be considered as severe complications because when possible surgical treatment can improve the history of the patient in terms of life expectancy and quality of life. The approach to these lesions should be multidisciplinary in collaboration with oncologists and radiotherapists, in fact the average of survival of these patients has increased in recent years thanks also to the evolution of anesthaesiological techniques that permit surgical treatments that were once considered prohibitive and the application of new adjuvant therapy that increases the effectiveness for surgical treatment.

**Prognostic Factors in Soft Tissue Sarcoma: an Analysis of 650 Patients**

Simon Meadowcroft, Owen Callender
*(253 Warwards Lane, Birmingham B29 7QR, England)*

*Keywords:* sarcoma, soft tissue neoplasms, neoplasm staging, prognosis

*Purpose:* To identify independent adverse clinical and pathological factors with respect to survival in patients diagnosed with soft tissue sarcoma (STS).
*Patients and methods:* Clinical and pathological data pertaining to patients receiving new diagnoses of STS at a single institution has been collected over the past 20 years. The study population of 650 patients excludes those presenting with retroperitoneal or metastatic disease and those who were lost to follow up within 2 years of diagnosis. Patient and tumour related variables which were postulated to affect prognosis were subject to univariate analysis. Multivariate techniques were then used to identify which variables were significant independent prognostic factors. The end point used was all cause mortality.
*Results:* The 5-year survival rate in the study population was 53 years. Surviving patients were followed up for a median of 45 months following diagnosis. Significant independent adverse prognostic factors were found to be (i) tumour grade 'high' ($P = 0.005$), (ii) large tumour size ($P = 0.005$) and (iii) tumour site deep to investing fascia ($P = 0.020$). Advanced age and tumours located in the proximal leg were found to be significant adverse prognostic factors in univariate analysis but lost their significance in multivariate analysis. No histological tumour type was shown to have a significantly different prognosis to any other. Excision of the presenting tumour with an inadequate surgical margin was not found to confer an adverse prognosis.
*Conclusion:* The independent adverse prognostic factors for survival in STS were found to be high grade, large size and location deep to the investing fascia.

**Surgical Nursing Protocol Takes Care of Children and Teenagers Affected by Osteosarcoma**

Dolores Colunga, Remiro Mirian, Marin Estrella
*(C/ Alfonso el Batallador, n[a]14 3A, Pamplona 31007, Navarra)*

In the University Clinic of Navarra, osteosarcoma has been treated since 1972. The multidisciplinary boarding of this kind of disease has been through chemotherapy, surgery and RIO.
After many years of experience and study, we decided make a new surgery nursing protocol in order to take care of children and teenagers who are affected by osteosarcoma with the aim of increasing the quality of care and to provide for the patients' integral cares.

**Surgical Treatment of Metastastic Tumors of the Chest Wall**

Antoio Briccoli, Rocca Michele
*(Modulo di Chirurgia Generale, Istituto Ortopedico Rizzoli, via Pupilli 1, Bologna 40136, Italia)*

Isolated metastatic tumors of the chest wall, in our series amount to 25% (21 cases) of the malignant tumors excised in this site. Histologically, carcinomas are the most frequent (seven colon, five breast, one kidney, one thymoma) compared to sarcomas (three soft tissue sarcomas, three bone sarcomas); one patient presented a rib metastasis from melanoma. Seventeen were located in the rib only, two in the rib and sternum, one in the rib and clavicle, and one in the sternum only. Fifteen cases complained of severe or medium intercostal pain, in five cases it did not pass with painkillers, and in six a progressively growing tender mass was seen. All cases had radical surgery (14 resections of one rib segment, three resections of more than one rib, two resections of rib and sternum, one resection of rib and clavicle, one subtotal sternectomy). In 10 cases postoperative chemotherapy was associated. One patient resected of the I, II, III rib and partial sternectomy died 30 days from surgery for thrombotic complications. Of the remaining 20 patients, half died after an average survival of 23 months (range: 2–55) from surgery, the other half is alive at an average of 29 months (range: 1–74); one patient is alive with local recurrence. Surgical treatment of metastatic tumors of the chest wall, when no disease is present elsewhere, is indicated because it is more efficient than any other treatment for the high quality of life of the operated patient. The majority of patients (71% in our series) who suffered severe pain due to infiltration of the intercostal nerves, after excision benefited from reduced or complete disappearance of pain.

**Palliative Radiotherapy for Spinal Metastases: a Reappraisal of Current Surgical Scoring Systems**

Yvette van der Linden, Sander Dijkstra, Antonie Taminiau, Jan Willem Leer
*(Department of Clinical Oncology, Leiden University Medical Center, Leiden 2300 RC, The Netherlands)*

*Background:* Bone metastases of the spinal column can be treated either with different surgical procedures or with external beam radiotherapy. Based on only surgical data, several scoring systems are used as a guideline to decide which treatment to apply. In 1990, Tokuhashi *et al.* formulated a scoring system using six prognostic factors including Karnofsky Performance Score, tumour type and presence of visceral metastases. Based on 64 patients, they proposed three prognostic groups for treatment; group A for palliative treatment (3 months overall survival (OS)), versus group B (6 months OS) and group C (22 months OS) for surgery. In 1997, Enkaoua *et al.* studied 71 patients and reduced the former into two prognostic groups: group A for palliative treatment (5 months OS), and group B for surgery (24 months OS). In 2001, Tomita *et al.* presented a scoring system using only three prognostic factors based on 67 patients. They proposed four prognostic groups for treatment; group A for supportive care (6 months OS), group B for palliative surgery (15 months OS), and group C (24 months OS) and group D (50 months OS) for more extensive surgery. Clearly, all scoring systems advocated an important role for surgery in the treatment of spinal metastases. In the prospectively randomised Dutch Bone Metastasis Study, 1157 patients with painful bone metastases were treated with radiotherapy using either 8 Gy single fraction or 24 Gy in six fractions. A subgroup of 342 patients had painful spinal metastases without signs of spinal cord compression at randomisation. A total of 70% responded to irradiation with relief of pain. Purpose of the present study was to analyse the validity of the surgery based scoring systems in selecting patients for treatment.

*Methods and materials:* All 342 irradiated patients with spinal metastases were reviewed using the scoring systems of Tokuhashi, Enkaoua and Tomita. For analyses, Kaplan–Meier curves and the log rank test were used.
*Results:* Applying Tokuhashi score: 2, 42, and 56% of irradiated patients were in group A, B, and C with mean 3, 8, and 13 months OS, respectively ($P < 0.001$). Applying Enkaoua score: 19 and 81% were in group A and B, with mean 5 and 13 months OS, respectively ($P < 0.001$). Applying Tomita score: 10, 23, 13 and 54% were in groups A, B, C and D with mean 4, 7, 5 and 15 months OS, respectively ($P < 0.001$).
*Conclusions:* With the use of the scoring systems as a guideline for the treatment of spinal metastases, a large proportion of irradiated patients would have been assigned to undergo surgical treatment. Survival in irradiated patients was less favourable than in surgically treated patients, most likely due to selection bias within the latter. The scoring system of Tomita was least effective in predicting survival. For the vast majority of patients with painful spinal metastases we consider radiotherapy superior to surgery. Surgery is indicated in limited cases, e.g., when radiotherapy is unsuccessful, long-term survival is expected, or when bone fragments endanger the spinal cord. A refined scoring system for the treatment of spinal metastases will be presented at EMSOS.

**Back to the Bike!**

Clare Wiiliams, Russell, Linn
*(Royal Orthopaedic Hospital, NHS Trust, Bristol Road South, Northfield, Birmingham B31 2AP, England)*

A case study of a 28-year-old male who underwent a right hemi-pelvic endoprosthetic replacement for grade 2 chondrosarcoma. He presented in 1996 following a 2-year history of hip pain and had surgery 2 months later. He had a one-stage hemi-pelvic replacement with an uncemented stanmore long stem femoral component. The greater trochanter was preserved and reattached. Following an initial uneventful recovery he dislocated and after a successful closed reduction mobilized in a Derby brace. Four months later he was walking short distances without an aid and pain free. Detailed presentation of nursing intervention and physiotherapy including functional assessment scores. Rehabilitation over the last 8 years including a pictorial account and short video demonstrating his return to a full function which includes riding his motorbike and water skiing.

**The Lived Experience of having Limb Salvage Surgery for the Treatment of Sarcoma**

Julie Woodford
*(The Royal National Orthopaedic Hospital, NHS Trust, Brockley Hill, Stanmore, Middlesex HA7 4LP, England)*

Cancer treatment predominantly focuses on cancer survival. Individuals live through cancer; however, as a complex, multi-faceted illness rather than a disease event. Cancer nurses have a responsibility to seek and interpret lived experiences of patients to ensure that therapeutic nursing care is delivered. Primary bone and soft tissue sarcomas are rare, affecting both men and women of all ages. The management of them is highly specialised and complex. In the United Kingdom it is undertaken within specialist treatment centres. Advances in radiographic imaging and multi-agent chemotherapy regimes have resulted in reconstructive surgery using endoprosthetic replacements rather than amputation having become standard treatment. Consequently, patients experience long-term outcomes affecting mobility, independence, self-concept and lifestyle.

This paper will report on the findings of a phenomenological enquiry undertaken to investigate the lived experience of having limb salvage surgery for the treatment of bone and soft tissue sarcomas to determine their caring needs. Rather than produce generalisable findings, this study aims to increase our knowledge and understanding by describing the experience 'as it is'. Five women were asked to describe fully, their lived experience of having limb salvage surgery during unstructured, in-depth formal interviews. The interviews were recorded and transcribed verbatim. By analysing the contents and contexts of the interviews and reflecting on the essential themes that characterise the experience five paradigm themes were conceptualised. These were validated by the study participants and include: Being Unprepared, Changing my Life, Losing Independence, Hoping to Improve and Being Careful. The implications for practice as a result of this study involve providing sensitive and comprehensive care that reflects the diversity, complexity and multiplicity of problems that patients face. By improving information regarding surgery and the expected outcomes and developing detailed individualised rehabilitation programmes within a framework of recovery and adaptation, patient satisfaction, quality of life and successful functional outcome will be maximised. Nursing research can contribute significantly to the development of patient-centred practice. By explicitly and visibly attaching importance to patient perspectives and making these the focus of research endeavours, challenges the traditional view of the patient as a passive recipient of care. The adoption of research methods such as phenomenology values the patient as an active contributor towards the design of future services.

**Is it Ethical to Avoid High Dose Methotrexate (HDMTX) Patients with Localized High Grade Osteogenic Osteosarcoma? A Macro-Analysis of the Literature**

Gérard Delepine, N. Delepine, F. Delepine, H. Cornille, S. Alkallaf
*(Service d'Oncologie Pédiatrique, Hôpital Avicenne, 125 Route de Stalingrad, Bobigny 93000, France)*

*Backgrounds:* As HDMTX is costly and potentially dangerous, some groups (for example: E.I.O., European Intergroup for Osteosarcoma) advocate protocols of chemotherapy without HDMTX in high grade osteosarcoma. We present here the results of a comprehensive literature analysis, to assess the comparative results of both approachs.
*Methods:* A computerized literature search encompassing January 1976 to March 2002 was conducted to identify all available published reports on clinical trials for localized primary osteosarcoma. All reports were reviewed to see if they matched the inclusion criteria: stage II limb osteosarcoma, at least 20 patients included, chemotherapy treatment with dosages and schedule clearly specified, clear quantification of 5 year DFS. HDMTX was defined by at least $12\,g/m^2$ per course for children ($< 12$), $8\,g/m^2$ per course for adults and a minimal total dose of MTX of $150\,g/m^2$.
*Statistical analysis:* Compared DFS in the group with HDMTX, and without HDMTX using non-parametric test ($U$-test).
*Results:* A total of 56 protocols fulfilled the inclusion criteria; 25 with HDMTX and 31 without HDMTX. The total number of patients included in eligible studies was 3956. Patients included in protocols without HDMTX had a 5-year average life expectancy of 48% (min. 24–max. 61) versus 72% for patients included in protocols with HDMTX (min. 53–max. 84). The difference is significant ($P < 0.001$).
*Conclusion:* Protocols without HDMTX offer a lower cure rate for patients with high grade osteosarcoma. The conclusions of EOI studies 1 and 2 were inadequate and their application decreased the disease free life expectancy of patients with osteosarcoma.

### Long-Term Results in Skeletal Reconstruction of Children with Bone Sarcomas

Marco Manfrini, G. Bianchi, C. Stagni, L.Campanacci and M. Mercuri
*(Department of Musculoskeletal Oncology, Istituto Ortopedico Rizzoli, Bologna 40136, Italy)*

Long-term results of limb-salvage surgery in 133 children, long survivors from bone cancer, are presented. From 1975 to 1992, 396 children younger than 15 years (mean age 10.8) were surgically treated at Istituto Ortopedico Rizzoli for bone sarcoma (318 osteosarcoma, 70 Ewing's sarcoma, seven MFH, one fibrosarcoma). A total of 130 patients were amputated, 26 had a rotationplasty, 29 had a skeletal resection without bone reconstruction (mainly for sites as fibula, scapula or clavicle), while 211 children received a segmental resection of part of their skeleton, immediately followed by the bone reconstruction with different techniques. At a minimum follow up of 10 years (mean 14 years, range 10–26), 133 reconstructed patients were clinically evaluated for radiographic and functional results. At the time of treatment the tumor was located in the femur in 66 patients, in the tibia in 42, in the humerus in 19, in the pelvic bones in four and in the radius in two cases. The resected skeletal segment (mean length 15 cm, range 8–29) included a joint surface in 96 patients that were reconstructed by 45 arthrodeses (six hip, 35 knee and four ankle) 45 prostheses (six hip, 21 knee and 18 shoulder), five osteoarticular allografts (two distal femurs, two proximal tibia, one proximal humerus) and one vascularized autograft (distal radius). In 37 cases the resection involved an intercalary segment of the femur (17 cases), tibia (18 cases), ileus (one case) or radius (one case). Synthetic implants were used in 69 cases while biological reconstructions by different types of bone grafts were performed in 64 patients. Because of septic or vascular complication during follow-up, 13 patients (10%) had a secondary amputation of the reconstructed limb. For various complications, 30 patients (23%) needed complete revision, while 20 patients (14%) had the partial revision of the primary implant. Other 30 patients received minor surgery. On the whole 70% of the cases underwent further surgical procedures related to the primary reconstruction. The infection of the implant was the major complication and occurred in 31 patients (23%). This outcome affected more synthetic implants (30%) than biological reconstructions (16%). According to MSTS evaluation scale, the final functional result was rated as Excellent in 17 cases (16 intercalary reconstructions and one osteoarticular vascularized autograft), Good in 36 cases (seven arthrodeses, 20 prostheses, one osteoarticular allograft, eight intercalary reconstructions), Fair in 43 cases (19 arthrodeses, 17 prostheses, three osteoarticular allografts, five intercalary reconstructions) and poor in 36 cases (19 arthrodeses, eight prostheses, one osteoarticular allograft, eight intercalary reconstructions). Limb-salvage surgery in children with bone sarcomas is confirmed to be a great challenge for orthopaedic oncologists. Despite the high percentage of complications and the risk for secondary loss of the reconstructed limb, long-term results of intercalary reconstructions, modular prostheses and osteoarticular grafts encourage us to go on in defining better surgical techniques for the reconstruction of child skeleton.

### Bone Metastases in Pediatric Osteosarcoma

Marco Manfrini, S. Ferrari, N. Fabbri, C. Stagni, P. Picci, F. Bertoni, G. Bacci
*(Department of Musculoskeletal Oncology, Istituto Ortopedico Rizzoli, Bologna 40136, Italy)*

In high grade osteosarcoma (OS), bone metastases may present at different stages. Skip metastases occurr closely to the primary lesion, in the bone marrow of the same skeletal segment or even on the opposite side of a joint. Their frequence is not well defined and their percentage is usually not reported. One or few distant bone lesions may accompany a classic OS presentation while multiple and diffuse bony lesions represent the 'multicentric' variety of OS. Then a bone metastasis may become evident during the post-treatment follow-up. To analyze incidence, pattern of presentation and role in prognosis of bone metastases in OS, the authors reviewed a series of 487 children (age range 2–14, mean age 11 years) all with the diagnosis of high-grade classic or teleangiectatic OS consecutively treated over a 25-year period (1976–2000) in the same institution. At presentation, skip metastases were demonstrated in 13 cases (3%), always in the tibia or in the femur. All these patients were surgically treated, but only two were alive at a mean follow-up of 96 months. Various distant bone metastases were shown in 15 cases (3%). Only five of these patients were surgically treated with the removal of all the evident bony lesions and only one is alive 180 months after surgery. A total of 394 stage II patients were treated by surgery and chemotherapy and then followed. Bone metastases appeared in 38 cases (10%) at a mean follow-up of 28 months (range 3–134 months). In 16 of these subjects, bone metastases were aggressively treated by surgery or radiotherapy and at a minimum follow-up of 36 months after the last treatment, eight patients were alive with no evidence of disease.

*Conclusions:* Diffuse multicentic OS occurs in 2% of children population with OS. These patients are usually not treated surgically and die in the first 2 years. Skip OS might be evident in 3% of OS patients; they must be searched for at presentation and must be considered as real metastases; usually they present in patients that do not show pulmonary metastases but their outcome is worst than that one of OS patients with nodules on chest CT and seem more similar to that one of OS patients with distant bone metastases. Metachronous bone metastases occur in the follow-up of 10% of children previously treated for OS and need to be aggressively managed; new treatment strategies need to be designed for a more effective approach to OS with bone metastases at presentation.

### Skeletal Anchorage of Amputation Prostheses – a New Concept

Karin Björnberg, Carin Lindén, Nina Ekwall, Lena Fryckberg
*(Ward 16, Department of Ortopedic Oncology, Gothenburg 413 45, Sweden)*

In 1960, Per Ingvar Brånemark developed skeletal anchorage of titanium fixtures to replace lost teeth. The same principle has been adopted during the last decade for skeletal anchorage of extremity prostheses, e.g., in patients ablated due to sarcoma. The first operation was performed in 1990. At present only patients who cannot use conventional socket prostheses are treated, e.g., due to skin problems or an extremely short stump. The surgery is performed as a two-step procedure. At the first surgical procedure the bone marrow is prepared and the titanium screw is anchored into the skeleton. The second procedure is performed 6 months later when the abutment is attached to the osseointegrated fixture. In order to make the skin heal to the bone all the subcutaneous fat over the bony stump is removed. Hereby movements between the skin and the abutment are minimized, which decreases the risk of infections. Six months later the patient can put full weight on the prosthesis. Studies have shown that these patients experience improvement in comfort, gait, pain, stump-problems and quality of life. All patients have better function after surgery. Skin problems are eliminated since no socket is used. The fact that the prosthesis is anchored to the skeleton leads to better balance and coordination and also a lower energy consumption. The prosthesis has a simple locking-device making it easy to attach. Above knee amputees with skeleton-anchored prosthesis have better ability to have the hip joint than patients with socket prosthesis. Notably these patients improve their prosthetic hand or foot sensation and can experience the feeling of different surfaces. This phenomenon is described as osseoperception. Possible complications are infections, loosening of the fixture and material fracture. Of the first 30 patients six had deep infections, one of which resulted in reamputation. Six of the fixtures had mechanical loosenings. Mechanical fractures occured in two fixtures and two abutments. Patient cooperation and motivation is mandatory. The skin and abutment must be thoroughly cleaned every day to avoid infection. Our department collaborates with colleagues in England and Australia. This promising method is experimental and under constant development. Hopefully, it will help amputees worldwide.

**Establishment of Osteosarcoma Xenograft Models from Patient Tumor Biopsies**

Adhiambo Witlox, H. Graat[1,2], B. Molenaar[2], J. Bras[3], V.W. van Beusechem[2], G. Schaap[4], W.R. Gerritsen[2], P.I.J.M. Wuisman[1]

*([1]Department of Orthopedic Surgery, VUMC, Amsterdam, The Netherlands, [2]Division of Gene Therapy, Department of Medical Oncology, VUMC, Amsterdam, The Netherlands, [3]Department of Pathology, AMC, Amsterdam, The Netherlands, [4]Department of Orthopedic Surgery, AMC, Amsterdam, The Netherland, van der boechorststraat 7, Amsterdam 1081 BT, The Netherlands)*

Research on the biology and treatment of osteosarocma (OS) requires OS tumor models that resembles OS in humans. In this respect OS tumor models deriving from cell lines are of limited value since they have lost characteristic features of OS. To provide more relevant tools for the study of OS we set on to establish xenograft tumor models in nude mice from fresh patient biopsies. Tumor biopsy material was received from seven patients. All but two tumors were high grade OS. In two cases both, biopsies pre and post chemotherapy and after surgical treatment were obtained. Fresh biopsy material was implanted directly subcutaneous onto nude mice. Mice were followed for maximal 250 days. If by then no tumor growth was observed mice were sacrificed. When tumor growth was observed mice were sacrificed when tumors exceeded 1000 $mm^3$. Other organs were macroscopically analyzed for metastases. Lungs were microscopically analyzed for metastases. Tumor pieces of $3 \times 3 \times 3$ mm were re-implanted into secondary recipients and these animals were followed again for maximal 250 days. From the nine biopsies implanted we could establish four representative tumor models, all derived from high grade OS biopsies, one from a patient post chemotherapeutic treatment. In none of these cases metastases were found. Immunohistochemical analysis showed that all four models maintained characteristic OS features, including ostoid production, spindle-cell pattern and pronounced nuclear atypia. These four well established and characterized models will be valuable to improve the treatment and the understanding of OS.

**Conditionally Replicative Adenoviruses Expressing p53 Exhibit Enhanced Oncolytic Potency on Primary Human Osteosarcoma Tumor Samples**

Adhiambo Witlox, H. Graat[1,2], J. Bras[3], V.W. van Beusechem[2], G. Schaap[4], W.R. Gerritsen[2], P.I.J.M. Wuisman[1], V.W. van Beusechem[2]

*([1]Department of Orthopedic Surgery, VUMC, Amsterdam, The Netherlands, [2]Division of Gene Therapy, Department of Medical Oncology, VUMC, Amsterdam, The Netherlands, [3]Department of Pathology, AMC, Amsterdam, The Netherlands, [4]Department of Orthopedic Surgery, AMC, Amsterdam, The Netherlands, van der boechorststraat 7, Amsterdam 1081 BT, The Netherlands)*

Conditionaly replicative adenoviruses (CRAds) are attractive agents for Osteosarcoma (OS) gene therapy, since CRAds are designed to selectively replicate in and to destroy tumor cells. The oncolytic potency of CRAds depends on their capacity to infect tumor cells and to induce cancer cell lysis. In this regard, we have previously identified two important limitations. First, primary OS cells express very low levels of the adenovirus attachment receptor CAR (*Journal of Gene Medicine* 2002; 4: 510–516) this problem could be solved by expanding CRAd tropism towards integrins. Second, CRAd replication in cancer cells is hampered by dysfunctional cell death pathway. To address this second limitation, we constructed a new CRAd derived from AdΔ24 (Fueyo *et al. Oncognene* 2000; 19: 2–12). This new CRAd, AdΔ24-p53, restores cell death in cancer cells by expressing functional p53. Compared to the parent AdΔ24, AdΔ24-p53 killed almost all cancer cell lines more effectively (*Cancer Res.*, 2002; 62: 6165–6171). Here we evaluated the efficacy of this CRAd on primary OS tumor samples. First a panel of short-term cultures was established from tumor biopsies of patients with OS. The functional p53 status of these primary cell cultures was investigated with a p53 specific transactivation assay. This showed that from the 12 samples tested, six samples had non functional p53, three samples had impaired p53 function, which may reflect tumor heterogeneity, and three samples had functional p53. Next, from these 12 samples a representative selection of six samples was made, cells were infected with AdΔ24 or AdΔ24-p53 at MOI 10 and cultured for 11 days, cell viability was quantified by WST-1 conversion assy. Under these conditions AdΔ24 was ineffective against all OS samples. In contrast, AdΔ24-p53 killed 6/6 OS specimens independently of the functional p53 status. These findings on clinically relevant OS tumor specimens suggests that a CRAd that restores p53-dependent cell death is a promising agent for OS treatment. Currently we are constructing a next generation CRAd, that is targeted towards integrins and expresses p53, for most effective OS treatment.

**Prognostic Factors in Soft Tissue Leiomyosarcoma of the Extremities: a Retrospective Analysis of 42 Cases**

Giovanni Beltrami, Alessandro Franchi*, Patrizio Caldora, Domenico Andrea Campanacci, Marinella Micol Mela, Daniela Massi*, Rodolfo Capanna

*(Centro di Chirurgia Oncologica e Ricostruttiva, CTO, Largo Palagi 1, Firenze 50139, Italy)*

*Intoduction:* Soft tissue leiomyosarcomas (LMS) are rare tumors which are associated with a poor prognosis. The goals of the present study were to describe the clinico-pathological features of 42 patients affected by soft tissue LMS of the extremities and to explore the prognostic impact of several clinical and pathologic parameters.

*Methods:* Forty-two patients with soft tissue LMS of the extremities were retrospectively studied. The following clinical and pathological parameters were analyzed: age, sex, site, size, depth, previous surgical procedures, stage, histotype, nuclear atypia, grade, mitotic activity, necrosis, surgical margins, therapy. Disease-free survival rates were calculated according to the Kaplan–Meier method. A multivariate analysis was used to determine which variable had an independent effect on clinical outcome.

*Results:* The overall 2- and 5-year disease-free survival rates were 42.3 and 32.6%, respectively. Overall, twenty-two (52.4%) patients developed disease progression, occurring between 1 and 34 months after diagnosis. By univariate analysis, tumor size, average mitotic rate, type of excision, and adjuvant radiotherapy, were significantly correlated with disease progression. By multivariate analysis, the only factor that was found to be an independent predictor of disease relapse was type of excision.

*Conclusions:* Soft tissue LMS of the extremities are rare, predominantly deep-seated neoplasms of adulthood, bearing an aggressive biological behavior. Large tumor size and high mitotic rate resulted adverse prognostic factors. Adjuvant radiation therapy, in combination with wide surgical excision, allowed the best chance of cure.

**Percutaneous Image-Guided Radio-frequency Ablation of Painful Metastases Involving Bone: a Multicenter Study**

Rodolfo Capanna[1], Patrizio Caldora[1], Domenico Campanacci[1], Giovanni Beltrami[1], Matthew P. Goetz[2], Matthew R. Callstrom[3], J. William Charboneau[3], Michael A. Farrell[3], Timothy P. Maus[3], Gilbert Y. Wong[4], Jeff A. Sloan[5], Paul J. Novotny[5], Ivy A. Petersen[6], Robert A. Beres[7], Daniele Regge[8], Mark B. Saker[9], Deitrich H.W. Grönemeyer[10], Kamran Ahrar[11], Michael A. Choti[11], Thierry J. de Baere[12], Joseph Rubin[13]
*([1]Department of Orthopedic Oncology, Florence, Italy, [2]Departments of Oncology, [3]Diagnostic Radiology, [4]Anesthesiology, [5]Biostatistics, and [6]Radiation Oncology, Mayo Clinic, Rochester, MN, USA, [7]St. Luke's Hospital, Milwaukee, WI, USA, [8]Cancer Institute, Torino, Italy, [9]Northwestern University, Chicago, IL, USA, [10]Inst. For Microtherapy, Bochum, Germany, [11]MD Anderson, Houston, TX, USA, [12]Johns Hopkins, Baltimore, MD, USA, [13]Gustave-Roussy, Paris, France)*

Over a period of 18 months, 43 adult patients suffering of osteolytic metastatic lesions involving bone underwent percutaneous CT or ultrasound-guided radiofrequency ablation (RFA) with a multi-tip needle under general or epidural anesthesia. Treated patients had less than or equal to two painful metastases and either failed or were poor candidates for conventional radiation treatment or chemotherapy. The patient's pain was measured using the standardized (1–10) Brief Pain Inventory (BPI) prior to the procedure and then sequentially for a total follow-up period of 6 months. The patient was considered eligible when suffering of greater than or equal to 4/10 worst pain over a 24-h period. Lesion sizes ranged from 1 to 18 cm. Median follow-up was 10 weeks. Prior to RFA treatment, the mean score for worst pain in a 24-h period was 7.9/10 with a range of 4–10/10. Four and 12 weeks after treatment, mean worst pain decreased to 4.4/10 ($P<0.0001$) and 2.9/10 ($P<0.0001$), respectively. Average pain prior RFA was 5.8/10 with the decrease to 2.8/10 ($P<0.0001$) 4 weeks and 1.8/10 12 weeks post treatment. Mean pain interference with general activity dropped from 6.6/10 to 3.7/10 ($P<0.0001$) at both 4 and 12 weeks post RFA. Two complications related to RFA were observed: one sacral lesion ablation resulting in transient bowel and bladder incontinence and a second resulting in a second-degree skin burn. Radiofrequency ablation of painful metastases is safe and effective in pain relief. RFA provides durable palliation and improvement in quality of life in patients with painful skeletal metastatic disease.

**Percutaneous Radiofrequency Ablation of the Osteoid Osteoma: is it the End of Surgery?**

Patrizio Caldora, D.A. Campanacci, G. Beltrami, A. Astone, P. De Biase, R. Capanna
*(Centro di Chirurgia Oncologica e Ricostruttiva, CTO, Largo Palagi 1, Firenze 50139, Italy)*

*Introduction:* Percutaneous radiofrequency coagulation has become an highly successful alternative to surgical treatment of the osteoid osteoma using long wavelength electromagnetic radiation to produce thermal ablation. The main orthopaedic oncological indications are the osteoid osteoma, little osteblastoma and painful metastases of carcinoma.
*Materials and methods:* From March 1998 to March 2002, 46 patients underwent percutaneous radiofrequency ablation for an osteoid osteoma (o.o.) in our Institution: 28 male and 18 female, with an average age of 23 years (min 3, max 57); 41 o.o. were located in the limbs, of which 21 (51%) in the femur and five in the spine. The average pain duration before treatment was 10 months (min 2; max 120). In 32 patients has been performed a peripheral anaesthesia while in nine general anaesthesia has been necessary.
*Technique:* Under CT guidance a Kirschner wire (bigger then the probe) was positioned inside and through the nidus with a hand drill. A catheter plastic guide was subsequently advanced over the K wire onto the bone and then utilised as a guide for the thermo-coagulating probe after removal of the K wire. By using a 1-cm tip length probe, attention has been taken to the final tip position (confirmed by CT scan) since no more than 5 mm of tissue around the probe will be burned. In tumours bigger than 1 cm, a 2-cm tip probe may be used or, in alternative, multiple coagulations or a multineedle probe (star-burst) will be necessary. In our experience a Radionics-Cool-tip RF Generator System has been used in all the patients. The electrode is gradually brought to 90°C working for 6 min on average.
*Results:* Patients have been evaluated considering the pain relief and CT control or MRI examinations were performed immediately following the procedure and after 1, 6, 12 and 24 months. At an average follow-up of 12 months (min 3, max 52) the procedure has been considered successful in 44 patients (95%). In two cases there was no pain relief: one because of an image simulating an osteoid osteoma and one not responding to ablation and undergone surgical resection. Two minor complications were observed: both skin-burns which spontaneously healed.
*Conclusion:* Percutaneous thermo-coagulation of the osteoid osteoma results to be an effective, simple and minimally invasive technique in alternative to the traditional surgical treatment. This procedure is particularly indicated in osteoid osteoma deeply located, requiring an aggressive surgical approach and for vertebral locations. In our experience this procedure was successful in the 95% of cases with no major complications.

**Limb Salvage by Vascularized Fibular Graft after Tibial Resection. Our Results after 10 Years Follow-Up**

Rodolfo Capanna[1], Giovanni Beltrami[1], Domenico Andrea Campanacci[1], Patrizio Caldora[1], Marco Innocenti[2], Marco Manfrini[3], Giuliana Roselli[4], Andrea Masi[4]
*([1]Department of Orthopedic Oncology, CTO, Florence, [2]Department of Reconstructive Microsurgery, CTO, Firenze, [3]First Orthopedic Clinic, IOR, Bologna, [4]Department of Radiology, CTO, Florence, Centro di Chirurgia Oncologica e Ricostruttiva, CTO, Largo Palagi 1, Firenze 50139, Italy)*

*Introduction:* In order to understand the clinical behavior and evolution of tibial reconstructions by vascularized fibular graft (VFG) with more than 10 years follow-up, we have performed a retrospective analysis of the cases treated in the Department of Orthopedic Oncology of Florence from 1988 to 1993.
*Material and methods:* They were 41 patients affected by bone sarcoma of the tibia, high grade in 28 cases (68%) and low grade in 13 (32%); average age was 18 (4–48) with a male/female ratio of 1.4. Intercalary resection and reconstruction were achieved in 32 cases, while nine patients had an arthrodesis (six knee and three ankle); average bone resection length was 14 cm. Tibial bone loss was reconstructed by free VFG in 40 cases, while in one case VFG was translated on its vascular pedicle. A massive allograft was associated in 31 cases (76%), while the VFG alone was used in 10 cases (24%).
*Results:* Major complications accounted for three cases of deep infection (7%) treated by graft removal and Ilizarov device in one case and by amputation in two cases. Minor complications (stress fracture, wound slough, transient neuroapraxia, ostheosynthesis breakage) occurred in 20 cases (49%), all healed by minor surgery or after immobilization. At the last follow-up, three patients had died of disease and three were lost. The authors report the clinical and radiographical results of 35 patients, with an average follow up of 136 months (122–185). All data have been discussed and analyzed, particularly regarding VFG hypertrophy and integration with massive allograft.
*Conclusion:* VFG reconstruction after bone tumor resection of the tibia showed to be a long-term (>10 years) reliable technique (86% of success rate) and represent the elective biological reconstruction especially in growing children.

### High-Activity Samarium-153-EDTMP Therapy followed by Autologous Peripheral Blood Stem Cell Reinfusion in Unresectable Osteosarcoma

C. Franzius[1], S. Bielack[2], S. Flege[2], H. Jürgens[2], O. Schober[1]
*([1]Department of Nuclear Medicine, University of Muenster, Germany, [2]Department of Pediatric Oncology, University of Muenster, Albert-Schweitzer-Str. 33, 48129 Muenster, Germany)*

*Aim:* Sm-153-EDTMP (samarium-153 ethylenediaminetetra-methylene phosphonic acid) has been introduced for palliative pain therapy of osteoblastic bone metastases. The distribution of Sm-153-EDTMP is comparable to that of Tc-99m-MDP in bone scintigraphy. Osteoblastic osteosarcomas producing abundant primitive bone matrix usually present as 'hot spots' on a diagnostic bone scan. Despite highly efficacious chemotherapy, patients with osteosarcomas still have a poor prognosis if adequate surgical control cannot be obtained. These patients may benefit from therapy with radiolabeled phosphonates.
*Patients and methods:* eight patients (four male, four female; 7–41 years) with unresectable primary osteosarcoma ($n = 5$) or unresectable recurrent sites of osteosarcomas ($n = 3$) were treated with high-activity of Sm-153-EDTMP (150 MBq/kg BW). Whole-body scans were acquired immediately after Sm-153-EDTMP application, 4 and 24 h later. In all patients, autologous peripheral blood stem cells had been asservated before Sm-153-EDTMP therapy. One patient had additional external radiotherapy of the primary tumor site. Four patients had additional external radiotherapy and polychemotherapy after Sm-153-EDTMP therapy.
*Results:* No adverse reactions were observed in the eight patients neither during the injections nor during the hours following the application. In one patient bone pain increased during the first 48 h after therapy. Another patient had complete pain relief within 48 h. Autologous peripheral blood stem cell reinfusion was performed on day +14 in all patients to overcome potentially irreversible damage of the hematopoietic stem cells. One patient with external radiotherapy and polychemotherapy is still free of progression 35 months later. Four patients died 6, 12, 15 and 20 months after therapy, the other three patients are still alive (follow-up 2–6 months).
*Conclusion:* These preliminary results show that high-dose Sm-153-EDTMP therapy is feasible and warrant further evaluation of efficacy. The combination with external radiation, and polychemotherapy seems to be most promising. Although osteosarcoma is believed to be relatively radioresistant the total focal dose achieved may delay local progression or even achieve permanent local tumor control.

### Skeletal Reconstruction by Extracorporeal Irradiation and Reimplantation of the Autograft after Tumor Resection: Long-Term Follow-Up

Gwen Sys, B. Poffyn, D. Uyttendaele, C. Dhooge, R. Forsyth, K. Verstraete, R. Verdonk
*(UZ Gent, Poli 5, De Pintelaan 185, Gent 9000, Belgium)*

We present our series of patients treated by extracorporeal irradiation and reimplantation of the autograft for skeletal reconstruction after tumor resection. Since 1978 we have treated 78 patients by this method. In 21 of these patients, 12 men and nine women, the follow-up ranges between 10 years 5 months and 24 years 5 months, with a mean of 16 years 6 months. The patients' age at diagnosis varied between 12 and 57, with a mean of 25 years. Twelve patients were in the second decade of life. Two tumors were benign, 19 were primary malignant bone tumors. In addition to surgical treatment, 14 patients also received chemotherapy, two also radiotherapy. There was one local recurrence requiring external hemipelvectomy. Two patients developed metastatic lung lesions, which were surgically removed. The majority of reconstructions involved the femur. There were only three intercalary reconstructions involving the diaphysis. For most of the reconstructions osteoarticular grafts were used with or without primary or secondary prosthetic joint replacement. At first, we reimplanted the complete irradiated graft, including the articular surface. In the medium term, this resulted in an avascular necrosis with complete destruction of the joint surface, requiring prosthetic replacement. Instability of the knee also was a major problem. Therefore we now only reimplant the diaphyseal part of the osteoarticular graft, and replace the articular surface by a prosthesis (knee, hip or shoulder). This procedure allows us to use a less massive prosthesis, and late prosthetic fatigue fractures can be avoided. Union of the osteotomy sites was rapid with abundant callus formation. Non-unions did not occur. One graft was retrieved after 24 years: histologically ingrowth of this graft over its full length and width was seen.
*Complications:* Two patients developed an early infection, one requiring amputation. Five patients developed an infection after a second operation. One graft diaphysis fractured after a fall 23 years postreimplantation.
*Conclusion:* This method of treatment has proven its long-term validity, and remains a valuable alternative to reconstruction by allograft replacement or massive prosthesis.

### Periosteal Osteosarcoma – Results of the EMSOS Study

Robert Grimer
*(Members of EMSOS, Royal Orthopaedic Hospitall, Birmingham B31 2AP, UK)*

*Introduction:* Periosteal osteosarcoma is a rare intermediate grade chondroblastic osteosarcoma arising on the surface of the bone. It has been described in medieval times from a cemetery in Budapest. The patient died. Modern treatment is surgical excision but there remains uncertainty as the necessity for chemotherapy. This study attempts to answer this question.
*Method:* Members of EMSOS were invited to contribute cases to this study and a proforma was devised to capture the requisite data. A total of 90 cases have been collected from a total of 10 different centres.
*Results:* The median age was 18 (range 8–65 years) and there was an equal number of males and females. The mean size of tumour was 10 cm. One patient had metastases at diagnosis. The most common sites were in the femur and tibia, followed by the humerus. A total of 63 patients had chemotherapy, of whom 47 had neoadjuvant treatment. Of these almost one-third had a good response (>90% necrosis). All patients underwent surgical resection, with 25 undergoing excision alone and 25 excision and bone grafting, 16 had endoprosthetic replacement. The margins were adequate (wide or better) in 76%. Local recurrence arose in eight patients, three with inadequate and four with adequate margins. Five of the eight patients subsequently died. In total 15 patients died, three of unrelated causes – one of a brain tumour and two of leukaemia. The overall survival of the patients was 88% at 5 years, 81% at 10 years and 75% at 15 years. The strongest predictor of a poor prognosis was local recurrence. Prognosis was not related to whether or not patients had chemotherapy nor to their response to chemotherapy.
*Conclusion:* Periosteal osteosarcoma remains a curable disease. The controversy as to whether or not chemotherapy is essential remains unanswered. Local recurrence is a poor prognostic factor.

**The Long and the Short of It!**

Robert Grimer
*(Royal Orthopaedic Hospitall, Birmingham B31 2AP, UK)*

*Introduction:* The median size of a sarcoma presenting to our unit is 10 cm whilst the median length of symptoms is 20 weeks. Controversy remains as to whether size (of tumour) and length (of symptoms) are of any importance in management or outcome for the patient. This study compares groups with long and short histories or sizes with those with 'average' dimensions to try and answer the question 'does size matter'?!
*Method:* A prospective database of all tumours referred to this unit has been kept since 1985. This database has been analysed to identify all patients with sarcomas and they have been split into groups based on size of tumour at presentation and duration of symptoms. For this analysis a small tumour was one < 4 cm whilst a large one was >20 cm. A short duration of symptoms was < 2 weeks and a long duration was >5 years. Details of 3548 sarcomas are available on the database with 41% being soft tissue sarcomas and the others bone sarcomas.
*Results:* Size was found to be important. The incidence of metastases at diagnosis was 12% for all tumours but was 4% for small tumours and 18% for large tumours. There was a wide range of sizes for STS but there were few very large or very small bone tumours. Surprisingly, whilst only 2% of tumours had a long history, 7% of the small tumours ( < 3 cm) gave a history of more than 5 years; 10% of synovial sarcomas had a long history. Length of symptoms proved an unreliable indicator of anything! It was not related to stage or histological grade at diagnosis. Treatment was related to size but not to symptoms with a higher incidence of amputation in patients with larger tumours. Prognosis was strongly related to size ($P < 0.0001$) of tumour, patients with small tumours having only one-fifth the risk of dying of large tumours. Duration of symptoms was unrelated to prognosis.
*Conclusion:* The mean size of breast lumps diagnosed as cancer is < 3 cm, that of sarcomas is 10 cm. Prognosis and treatment are both badly affected by size. The message is quite clear – sarcomas need to be diagnosed earlier. How we do it is another question. . . .

**Synovial Sarcoma Arising from Synovium – It Does Exist!**

Ashwin Kulkarni, H. Aherns, C. Mangham, R. Grimer, S. Carter, R. Tillman, A. Abudu
*(The Royal Orthopaedic Hospital, Bristol Road, Northfield, Birmingham B31 2AP, UK)*

*Purpose:* To review our experience of intra-articular synovial sarcoma, including presentation, pathological features, management and outcome.
*Method:* Retrospective analysis of prospectively collected data, review of imaging, pathology and patient outcome.
*Results:* Five patients were identified to have intrarticular synovial sarcomas from the prospectively collected oncological database at our institute with a follow-up to 8 years. Average age was 27 (10–68) and two were female patients. All patients had presented to a general orthopaedic surgeon with a long history of swelling, pain and effusion of up to 4 years. All of them had either arthrotomy or arthroscopy and synovial biopsy which confirmed the diagnosis.
*Conclusion:* Intra-articular synovial sarcoma remains a rare condition, which is likely to almost invariably be an incidental finding. Based on our experience we would recommend an initial cautious approach with arthroscopic excision and close follow up. Local recurrence should be treated aggressively with wide excision – almost certainly requiring an extra-articular resection. At relatively short follow up the prognosis seems remarkably encouraging!

**Excision and Reconstruction of Metastatic Tumours to the Extremity Bones: Outcome of 130 Consecutive Patients**

Ashwin Kulkarni, A. Abudu, R.M. Tillman, S.R. Carter, R.J. Grimer
*(The Royal Orthopaedic Hospital, Bristol Road, Northfield, Birmingham B31 2AP, UK)*

*Purpose:* The objectives of treating patients with tumour metastasis to the extremity bones are to improve pain and mobility and to control the disease locally but there are risks involved in doing major surgical procedures.
*Patients and methods:* Retrospective analysis of prospectively collected database of 130 patients over a 10-year period who were treated surgically at our institute for tumour metastasis to the extremity.
*Results:* The mean age at diagnosis was 61 years (22–84) and 68 patients were male. Lower extremity was involved in 104 (femur 80, tibia 11) and upper extremity in 26 (humerus 19). Metastatic disease was solitary in 55 patients and multiple in 75 at the time of surgery. The tumours originated from a variety of organs with renal cell carcinoma (50 patients), breast carcinoma (30 patients) and squamous carcinoma (10) being the most common. The indication for surgery was radical treatment of solitary metastases with curative intent in 33, pathological fracture in 46, impending fracture in 27, failure of prior fixation devices in 17, pain in 37. Surgical treatment included excision without reconstruction in 20 patients and resection with endoprosthetic reconstruction in 110 patients. Seven patients received adjuvant chemotherapy and the majority received adjuvant radiotherapy. At the time of review, 58 patients had died at a mean time of 23 months (0–90) from surgery (53 of progressive metastatic disease and five from other causes). A total of 72 patients were alive at mean follow-up of 22 months (1–103) from surgery. A total of 36 patients were alive at 2 years and eight at 5 years out of 130 patients. One patient died intra-operatively. Post-operative complications occurred in 32 patients (25%). General complications included chest infections (2%) and thrombo-embolism (7%) includingone fatal PE. Specific complications were four prosthetic dislocations, three deep prosthetic infection, two nerve palsies and two paraplegias due to spinal metastasis. A total of 75% patients achieved the objectives set before start of the treatment without any complications, 15% achieved objectives with some complications and 10% could not. There was no difference in the survival of patients who presented with solitary and multiple metastases.
*Conclusion:* We conclude that selected patients with bone metastases can benefit from resection and major bone reconstruction with acceptable morbidity.

**Outcome of Limb Salvage Surgery using Endoprostheses – Minimum Follow-Up of 10 Years**

Ashwin Kulkarni, L. Jays, R. Grimer, S. Carter, R. Tillman, A. Abudu
*(The Royal Orthopaedic Hospital, Bristol Road, Northfield, Birmingham B31 2AP, UK)*

*Aim:* To assess the outcome in terms of limb salvage and complications in patients who underwent endoprosthetic replacements more than 10 years ago.
*Method:* Retrospective review of prospectively collected database. All patients who underwent limb salvage surgery by an endoprosthesis prior to 31/12/1992. The database collects information about the patients, their tumours, their management and the complications as well as outcomes and functional scores.
*Results:* A total of 677 patients had endoprosthetic replacements prior to 31/12/1992 at our centre. The most common diagnosis was osteosarcoma (327 patients) followed by chondrosarcoma (81) and Ewings sarcoma (78). 576 patients had the EPR for primary bone malignancy, 55 for metastases and the remainder for benign conditions. The median age was 21 years (7–80 years). The most common site was the distal femur (257) followed by the proximal tibia (144). A total of 101 children had extendible endoprosthesis. Of the 677 patients, 359 are still alive. The survivorship at 10 years is 53%. The most common complications to arise following EPR were infection and local recurrence. The risk of infection was 8% at 1 year, 13% at 5 years and 17% at 10 years in the surviving patients. The risk of amputation was 11% at 5 years and 13% at 10 years with the majority being due to local recurrence followed by infection. Revision surgery was required in many surviving patients. The risk of revision was related to the onset of complications and to the site of the prosthesis. The revision rate was greatest in young patients with prostheses around the knee.
*Conclusion:* Limb salvage surgery prior to 1992 was on the whole quite successful even though many of these patients had their operation in the infancy of limb salvage surgery. There was a high incidence of complications. These results will act as a benchmark for comparison with more up to date series.

**Function Re-visited following Endoprosthetic Replacement (EPR) Involving the Knee**

Clare Williams, J. Davies
*(Royal Orthopaedic Hospital NHS Trust, Northfield, Birmingham B32 2AP, England)*

*Background:* At EMSOS 2002 we presented the results of our research investigating the influence of Rehabilitation on the functional outcome of patients under going endoprosthetic replacement involving the knee. The results were encouraging but only represented the first 6 months post surgery. In order to investigate the long-term function following limb salvage surgery the same 27 patients were re-evaluated 12 months later.
*Aims:* To ascertain the long-term level of function of patients undergoing distal femoral and proximal tibial EPR for the treatment of bone tumours.
*Method:* Patients completed two questionnaires: (1) The Toronto Extremity Salvage Score (TESS) and (2) the Euroquol Health Score. These were originally collected pre-operatively then at 6 weeks, 4.5 months and 10 months. For the purpose of this study they were then re-evaluated at 18 months.
*Results:* Of the original 27 patients, 17 follow-up questionnaires were completed.

| Type of Operation | TESS | Below 50% | TESS 50%–60% | TESS above 65% |
|---|---|---|---|---|
| Distal femoral EPR | 8 | 2 | 2 | 4 |
| Proximal Tibial EPR | 9 | 0 | 2 | 7 |

*Conclusion:* Results suggest that function is maintained and in many cases continues to improve in the long-term. Reasons for incomplete data and loss of function will also be presented.

**How Bad is a 'Whoops' Procedure? Answers from a Case-Matched Series**

Ashwin Kulkarni, R. Grimer, S. Carter, R. Tillman, A. Abudu
*(The Royal Orthopaedic Hospital, Bristol Road, Northfield, Birmingham B31 2AP, UK)*

*Introduction:* A 'whoops' procedure is when a lump, which subsequently turns out to be a soft tissue sarcoma (STS), is shelled out by a surgeon who is not aware of the diagnosis. In many cases residual tumour will be left behind necessitating further surgery. The significance of a whoops procedure in terms of survival and local control remains uncertain. This study has used case-matched controls to compare outcome between two groups.
*Method:* Patients referred to our unit following a 'whoops' procedure usually undergo re-excision of the area involved and are then managed like 'virgin' STS – i.e., ones which were untouched prior to referral. We have compared patients with 'whoops' procedures with 'virgins' matched by known prognostic factors, i.e., grade, depth, patient age, site, size and diagnosis of the tumour. We have investigated outcome in terms of local control, metastatic disease and survival by known prognostic factors and by their status at presentation.
*Results:* A total of 96 patients with a whoops procedure were compared with 96 referred directly to our unit. Despite attempts to match patients with as many variables as possible there was a tendency for the patients with whoops to have smaller tumours that were subcutaneous; they were, however, well matched for grade and stage at diagnosis. 23% of the whoops patients underwent further surgery whilst 15% of the virgins had the tumour excised. The eventual margins of excision were deemed adequate in 51% of the whoops and 59% of the virgin cases. The proportion having adjuvant radiotherapy and chemotherapy were 51 and 59%, respectively. The rate of local recurrence was 25% for the whoops cases compared with 19% for the virgin cases. The overall survival was however worse in the virgin cases. Prognostic factors for overall survival included size and grade. The type of status at presentation was NOT significantly significant either for survival or local control in this analysis.
*Conclusion:* Inadvertent surgical excision of a STS is not desirable but does not seem to lead to an adverse outcome in this series in which wide re-excision of the area involved has been carried out.

**An Audit of Fatigue Incidence in People having an Endoprosthetic Replacement due to Oesteosarcoma, Chondrosarcoma, GCT, or Ewing's Sarcoma**

Colin Jones, Jackie Lunn
*(The Royal Orthopaedic Hospital, NHS Trust, Northfield, Birmingham B32 2AP, United Kingdom)*

While working as an Occupational Therapist in Oncology at the Royal Orthopaedic Hospital, Birmingham, UK, fatigue seems to be an issue that is frequently raised, though to a varying degree by patients and the Oncology team. It seems that a patient who has had an Endoprosthetic replacement (EPR) suffered from fatigue at various stages; pre-operative, surgical/admission, and 6 weeks later, during a physiotherapy week. The effect that the fatigue seems to have on an individual's quality of life also varies but activities of daily living are investigated more in an acute hospital setting because of the need to enable safe discharges. In some cases the fatigue experienced was very disabling, so reducing functional ability and meant that patients were unsafe without more support services and equipment. Such issues needs to be organised and can have the effect of extending admission times, an unsatisfactory state for all concerned. Cancer-related fatigue (CRF) is subject to many research articles. Its definition is difficult due to the use of several words, which describe the same symptoms (Brown, 1999). CRF has been highlighted as a significant problem for the majority of patients experiencing cancer (Irvine *et al.*, 1991; Stefz *et al.*, 1995) (as stated by Schartz, 1998). CRF is the third most frequently cited issue in research after pain, nausea and vomiting. A total of 22% thought fatigue could be relieved (Stone *et al.*, 2000). During the treatment period, patient may receive chemotherapy and radiotherapy, which are also known to cause fatigue in patients (Irvine *et al.*, 1991). The research identifies fatigue as affecting a person's Qualify of Life (QOL) due to it affecting all aspects of their Activities of Daily Living (ADL). This is a core aspect of Occupational Therapy. The body of evidence also identifies factors relating to Nursing, Medical Interventions, Dietetics and Physiotherapy. This raises CRF to be a multi-disciplinary issue, which requires a similar approach for the assessment and treatment of CRF. As statistical data varies from study to study, as well as depending upon which type of cancer under investigation, it was decided to conduct a specific audit.
*Aim:* The audit aim was to identify the incidence and prevalence of fatigue within the specific population of patients listed for an EPR. The population for the audit was based at the Royal Orthopaedic Hospital NHS Trust and focuses on the main stages of treatment. Admission time (biopsy and clinic stage), post surgery on the ward before discharge home and 6 weeks post surgery upon re-admission for a physiotherapy week. The audit has been executed over a 2-month period, using a standardised multidimensional fatigue measure. The measure utilised was the Multidimensional Fatigue symptom Inventory-short form, as developed by the Moffitt Cancer Center and the University of south Florida.
*Results:* The presentation will detail the Audit's results, and identify the impact that they may have on the occupational therapy service in particular, at the Royal Orthopaedic Hospital. The measure will identify and classifies the styles of CRF being experienced by the patient, which may lead to the development of a CRF treatment programme.
*Conclusion:* In itself the audit has provided a preliminary evidence base where none could be found beforehand in published literature. It is hoped that further investigation can be made into a non-invasive treatment of cancer-related fatigue can be entered into in the near future, one that would comprise a multi-professional team approach.

**Curettage and Phenolisation is a Safe Treatment for Low-Grade Chondrosarcoma: 5-Year Follow-Up Results**

Suzan H.M. Verdegaal, P.C.W. Hogendoorn, A.H.M. Taminiau
*(Koningin Emmakade 76, The Hague 2518 RM, The Netherlands)*

*Introduction:* Since 1994, we started to treat patients with low-grade central chondrosarcoma of the extremities with intralesional surgery (curettage) in combination with adjuvant therapy (phenol). Before, patients were treated by local resections with wide margins. Although it is adequate therapy, morbidity is the concern.
*Aim:* Treatment with curettage and phenol safe for low-grade central chondrosarcoma?
*Materials and methods:* From 01/01/1994 till 31/12/1999, 23 patients were treated by curettage and phenol for borderline and grade I central chondrosarcoma of the extremities. Excluded are low-grade chondrosarcoma of juxtacortical origin, or chondrosarcoma secundary to osteochondroma. All cases were controlled by yearly X-rays and repeated dynamic MRI.
*Results:* A total of 23 patients (seven male, 16 female), had surgery for low grade chondrosarcoma of the extremities. The mean follow-up was 4.9 years (3–8.2). PA-diagnosis: borderline (9), grade I chondrosarcoma (14). Localisation: proximal humerus (5), metacarpals (3), phalanx (1), distal femur (10), tibia (3), fibula (1). Complications: one patient with a grade I chondrosarcoma of the right distal femur had a fracture 3 weeks after surgery. He was treated with osteosyntheses, and recovered without any problems. Recurrence: one patient with a borderline chondrosarcoma of MC II had symptomatic recurrence after 2 years. PA diagnosis: borderline chondrosarcoma. After surgery he is free of recurrence for almost 5 years now. All cases are controlled by yearly X-rays and dynamic MRI-scans. Besides the one recurrence, no other recurrences or metastasis was seen, and all patients are still alive.
*Conclusion:* A total of 23 patients, with low-grade or borderline chondrosarcoma, were treated by intralesional surgery, followed by adjuvant therapy (phenol). With an average follow up by dynamic MRI of almost 5 years, only one intraosseus recurrence, detected by MRI was seen, without progression of tumourgrade. This technique therefore seems to be a safe one, although longer follow-up by yearly repeated MRI is necessary.

**Neoangiogenesis in Adult Soft Tissue Sarcomas (STS): Prognostic Significance of Microvessels Density (MVD) Correlated to Grading and Stage. A Perspective Study**

Alessandro Comandone, A. Boglione, E. Berardengo, A. Bernardi, E. Brach del Prever, P. Bergnolo, O. Dal Canton, S. Chiadò Cutin, P. Pochettino, C. Oliva, G. Gino. Piemontese Group for study on Sarcoma and Rare Tumors and Italian Sarcoma Group
*(Ospedale Gradenigo, C.so Regina Margherita 8, Torino 10153, Italy)*

Angiogenesis is a multistep phenomenon, critical for tumor growth and prognosis in many solid neoplasms. Microvessels density (MVD) is a method of assessing angiogenesis and adversely affects DFS and OS in breast, lung and colorectal cancer. Few data are available in STS in which stage and grading are until now fundamental. Our perspective study determined the level of MVD in a series of STS and correlated these results with DFS and OS, comparing its prognostic value with grading and stage. MVD was determined with CD 31 immunostains in formalin fixed, paraffin embedded tissues. Intratumoral MVD was assessed by light microscopic analysis. Hot spots defined the positive areas. The study included 45 patients, 35 with localized and 10 with metastatic disease at diagnosis. All tumors were located in upper or lower extremities. Histology: 13 liposarcoma, 11 MFH, five leiomiosarcoma, five PNST, three rabdo, three synovialsarcoma, three undifferentiated and two fibrosarcoma. Following Coindre classification 23 patients had low grade and 22 high grade STS.
*Results:* Median follow-up is 23 months (2–84). At present, 20 patients (44.4%) are alive and DF, 11 (24.4%) alive with disease, 14 (31.1%) dead. Median survival is 75 months. Median MVD of all specimens is 62 microvessels/mm$^2$ (7–161). A total of 32 patients (71.1%) have low MVD (group A) and 13 patients (29.9%) high MVD (group B). Mean survival is 62.7 months in group A (median 75) and 36 months in group B (median not reached) (*P* 0.01); median DFS respectively 24 and 15 months (*P* 0.01). There is also a significant association with histological grade and survival: 75 months in low grade and 34 months in high grade tumors (*P* 0.05) and presence of metastasis at diagnosis (median survival: M+, 23 months, M·, 75 months). Unfortunately no relationship between angiogenesis and grading is found. In conclusion our study confirms the prognostic importance of grade and staging in patients with STS. Moreover the role of MVD in prognosis is well defined and should be used as a routine marker in STS histological diagnosis.

**Apoptosis and Cell Cycle Arrest by α-Tocopheryl Succinate in Osteosarcoma Cell Lines**

MariaSerena Benassi, R. Alleva, M. Tomasetti, L. Pazzaglia, F. Ponticelli, P. Picci
*(Laboratorio di Ricerca Oncologica, Istituti Ortopedici Rizzoli, Via di Barbiano 1/10, Bologna Ist. Patologia Sperimentale, Univ. Medicina, Ancona, Bologna, Ancona 40136, Italy)*

Several cellular mechanisms are involved to prevent proliferation of mutant cells, including apoptosis and arrest of cell cycle progression. Recent evidence suggests that α-tocopheryl succinate (α-TOS), a redox-silent analogue of vitamin E, is a strong inducer of apoptosis in several malignant cell lines. Although precise mechanisms of apoptosis induction by α-TOS remain to be elucidated, there is evidence that this process involves both the cell cycle arrest and membrane destabilising activities of this analogue. Here, we investigated the response to α-TOS treatment of two osteosarcoma cell lines (MG-63 and U2OS), with deregulated G1 cell cycle control. U2OS cell line was highly sensitive to concentrations of α-TOS as low as 30 μM, showing visible signs of apoptosis associated with accumulation of phospho-SER20 p53. No apoptogenetic activity was observed in MG-63 lacking p53 oncosuppressor gene. However, our preliminary results showed a lengthening of S phase in both MG-63 and U2OS after 48 h of α-TOS treatment, revealing an increased S-G2 cell cycle delay. Furthermore, α-TOS significantly reduced E2F1 phosphorylation by cyclinA-cdk2, increasing the binding of cyclinA to E2F1. These results suggest that α-TOS might be used to sensitised malignant cells to drugs destabilising DNA during its replication.

### Single Nucleotide Polymorphism in FGFR4 were Correlated with Prognosis in Bone and Soft Tissue Sarcoma

Yuki Morimoto, Toshifumi Ozaki, Toshiyuki Kunisada, Yasuko Nakagawa, Aki Yoshida, Hajime Inoue
*(Science of Functional Recovery and Reconstruction, Graduate School of Medicine and Dentistry, Okayama University, Shikata-cho 2-5-1, Okayama 700-8558, Japan)*

*Introduction:* Fibroblast growth factor receptor 4 (FGFR4) is one of tyrosine kinase receptors which play a critical role in cell proliferation, differentiation, apoptosis, and tumorgenesis. Recent study reveals that FGFR4 has single nucleotide polymorphism (SNP) at codon 388 (Gly to Arg), and breast and colorectal cancer patients who have homo- or heterozygous carriers of Arg388 allele were associated with poor prognosis. In this study, we investigated the relation between FGFR4 SNP at codon 388 and clinical results in bone and soft tissue sarcomas.
*Material and methods:* Samples of high-grade bone and soft tissue sarcomas were obtained from 104 patients operated at the Okayama University Hospital between 1986 and 2002. Median age at the diagnosis was 30 years, (range 7–79 years). There were 58 male and 46 female. Histological diagnosis was as follows: 41 osteosarcomas, 22 MFHs, 18 synovial sarcomas, seven Ewing's tumor, six MPNSTs, and 10 others. Median follow-up was 58 months (range, 12–203 months). Peripheral blood was also obtained from 102 Japanese healthy volunteers for control without apparent disease. For PCR-RFLP, genomic DNA was isolated by standard methods. PCR amplification of exon 9 of FGFR4 was done and the PCR products were digested with the restriction enzyme *Bst*NI. Association of FGFR4 genotype between patients and controls was analyzed by use of $\chi^2$-test. Kaplan–Meier analysis and log-rank test were used for survival analysis. Because of the small number of patients with FGFR4 Arg/Arg allele, we divided patients into two groups for survival analysis: Group G consisted of patients who had FGFR4 Gly/Gly alleles and group A consisted of patients who had at least one FGFR4 Arg allele. A $P$ value of $< 0.05$ was considered to be statistically significant.
*Results:* In bone and soft tissue sarcoma, 45 (43%) of 104 patients were homozygous for Gly allele. Patients who had heterozygous and homozygous Arg allele were 47 (45%) and 12 (12%), respectively. Healthy controls were as follows: Gly/Gly allele in 39(38%), Gly/Arg allele in 50 (49%), and Arg/Arg allele in 13 (13%). The frequency of FGFR4 genotype distribution between patients and controls was not significantly different ($P = 0.76$). In cumulative overall survival, patients in group G had a significantly better prognosis than patients in group A (70 vs. 57%, $P = 0.021$), and cumulative metastasis and disease free rate patients in group G were higher than those of patients in group A ($P = 0.28$ and $P = 0.32$, respectively). There was no correlation of relapse free rate between local recurrence and FGFR4 SNP ($P = 0.81$). Although there was no correlation of overall survival between bone and soft tissue ($P = 0.91$), a correlation between SNP in FGFR4 and prognosis was observed in soft tissue sarcomas (STS) ($P < 0.01$), but not in bone sarcomas ($P = 0.96$).
*Discussion:* Although samples were composed of various histological diagnoses, there was significant correlation between FGFR4 polymorphism and overall survival in patients with bone and soft tissue sarcomas. However, the examination of more samples is mandatory because we have only small samples classified by diagnosis when we assessed the individual influence of FGFR4 SNP for histological types. To our knowledge, there has been no previous report of SNP relating to clinical outcome in bone and soft tissue sarcomas. Because there was a strong influence of SNP in FGFR4 on prognosis in STS patients, SNP could improve prediction of clinical outcome and lead to design of new treatment in STS.

### Hazard Plots in Sarcoma Surgery

Paul Cool, Rob Grimer
*(Royal Orthopaedic Hospital, Bristol Road South, Northfield, Birmingham B31 2AP, UK)*

*Aim:* To demonstrate that hazard plots are useful in assessing the frequency of adverse events in sarcoma follow-up.
*Method:* A prospective database with 3548 sarcoma cases has been used to generate survival curves and to compare these with hazard plots both for the development of local recurrence and metastases.
*Results:* The first graph below shows the actuarial survival of patients without local recurrence or metastases over a 150-month time period. This gives a general impression of risk and an absolute value over the whole time period. The second graph shows the hazard plot with the risk of developing both local recurrence and metastases shown over the same time period, split into 12-month intervals. This shows the very heightened risk of recurrence in the second year, which is not apparent from the survival curve.
*Discussion:* It is an easy matter to generate graphs for all different types of tumours both for local relapse or onset of metastases. It is also possible to produce graphs for individual patients based on any other risk factors (e.g., margins of excision, effectiveness of chemotherapy, etc.). This can be used to tailor make decisions regarding follow up frequency and use of investigations (e.g., patients at high risk of local recurrence).

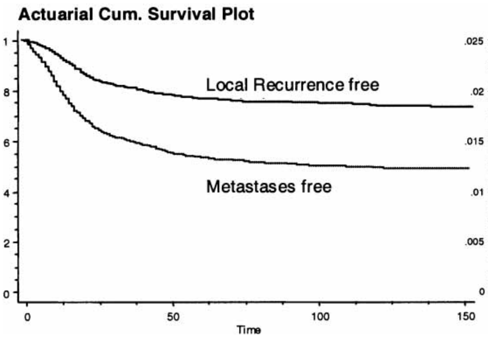

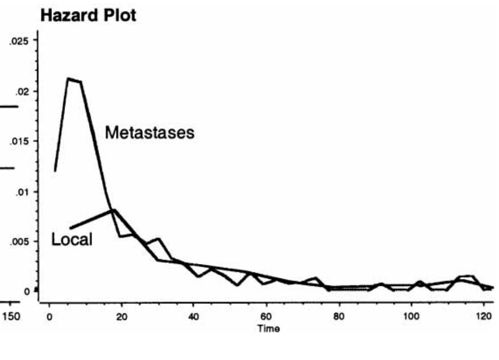

*Conclusion:* Hazard plots offer an improved method of assessing risks for following up patients.

**Preoperative High Dose Chemotherapy plus Autologous PBSCT and Limb Salvage in Non-Metastatic High Grade Osteosarcomas**

Kaan Erler, Mustafa Basbozkurt, Fikret Arpaci, Bahtiyar Demiralp, M.Taner Ozdemir, Ethem Gur
*(Department of Orthopedics and Traumatology, Gülhane Military Medical Academy, Etlik, Ankara 06018, Turkey)*

*Aim:* The optimal chemotherapy regimen is not established in patients with osteosarcoma. In those patients dose–tumor response curve is linear and the correlation between dose intensity and response rate or survival has also well known. In recent years it has been shown that, megatherapies with stem cell support have improved the survival, especially in metastatic disease. We want to present our preliminary report with this protocol in 22 cases.
*Material and method:* Between 1996 and 2002, 22 patients (19 male, three female) with median age of 22 (16–24) underwent high dose chemotherapy, autologous PBSCT (peripheral blood stem cell transplantation) and thereafter limb salvage surgery at the authors' institute. All patients had stage II B osteosarcoma and subtypes were 20 conventional, one high grade periosteal and one telengiectasic osteosarcoma. Localizations of the tumor were femur in 15 cases, proximal fibula in two cases, tibia in three cases, proximal humerus and iliac wing in one case. An induction chemotherapy including cisplatin, adriamycin and ifosfamide was given to the patients two times every 3 weeks. Fifteen days later stem cells were collected. High dose chemotherapy consisting of ifosfamide 15 g/m$^2$, etoposide 1.5 g/m$^2$ and carboplatin 1.5 g/m$^2$ (total dose) were given to the patients. Complete and permanent engraftment was observed in all of them. Limb salvage procedures were done in all patients except one patient (iliac wing).
*Results:* Mean follow-up is 23.4 months. Necrosis more than 90% was seen in 20 cases after high dose chemotherapy. The same regimen of induction chemotherapy was given in these patients. All patients except one who refused surgical procedure have been disease-free for a median of 20.4 (7–54) months. Disease-free survival is 42%.
*Discussion:* According to preliminary results of our ongoing study, preoperative high dose chemotherapy seems to be well tolerated with high response rate.

**Muscle Strength after Total Femoral Replacements due to Osteosarcoma**

Kaan Erler, Yavuz Yildiz, Bahtiyar Demiralp, M.Taner Ozdemir, Mustafa Basbozkurt, Ethem Gur
*(Department of Orthopedics and Traumatology, Gülhane Military Medical Academy, Etlik, Ankara 06018, Turkey)*

*Aim:* Total femoral replacement has been performed since 1952 and even became a well defined procedure for years; total resection of the femur due to bone sarcomas has remained infrequent. Great amount of bone and muscle are sacrificed with this procedure and assessment of the muscle strength of preserved limb is essential to improve functional results. We investigated two patient's limb functions whose were applied special rehabilitation program and isometric dynamometry.
*Material and method:* Two male patients, aged 20, diagnosed as femoral osteosarcoma (stage IIB) were studied. Case 1 referred to us because of pathological fracture of the midshaft; postoperatively this patient suffered temporary sciatic nerve palsy. En bloc resection and cemented, unipolar, modular endoprosthetic reconstruction, thereafter four-stage rehabilitation program were applied to both patients. Eight months after isometric dynamometry (Cybex Norm TM) evaluation on 60 and 180°/s was performed.
*Results:* Average follow-up was 15 months (14–16). Mean knee flexion was 100° and no limitation on extension. No local or prosthetic complication was observed. One patient who had pathological fracture underwent metastatectomy at lung in the 13th month. Cybex results on 180°/s showed significant muscle strength decrease after the operation. Deficits were calculated and contrlateral extremity considered as control. Concentric hip deficits mean scores: flexion 42% (35–49), extension 69% (75–63), abduction 50% (35–65), adduction 43% (53–33). Concentric knee deficits mean scores: flexion 13% (13–12), extension 41% (39–23). Enneking functional scores were excellent at last follow-up and patients are ambulating without external support.
*Discussion:* Achieving wide margins by total resection of the femur includes two major joints of the body; hip and knee. Therefore complications were relatively high. Functional muscle-prosthesis attachments are important for better scores and less complication rate. Our study demonstrated that hip scores decreased about half of normal scores. The most affected function is hip extension and knee scores are obviously better than hip scores. Even total femoral resection scarifies large amount of muscle, assessment of muscle strength may helpful for further planning of soft tissue reconstruction and determination of special requirements on rehabilitation program.

**Osteosarcoma of the Pelvis: Experience of the Cooperative Osteosarcoma Study Group**

Toshifumi Ozaki, T. Ozaki, S. Flege, M. Kevric, N. Lindner, R. Maas, G. Delling, R. Schwarz, A.R. von Hochstetter, M. Salzer-Kuntschik, W.E. Berdel, H. Jurgens, G.U. Exner, P. Reichardt, R. Mayer-Steinacker, V. Ewerbeck, R. Kotz, W. Winkelmann, S.S. Bielack
*(Department of Orthopaedic Surgery, Okayama University Postgraduate School of Medicine, and Dentistry, Okayama 700-8558, Japan)*

*Purpose:* To define patients and tumor characteristics as well as therapy results, patients with pelvic osteosarcoma who were registered in the Cooperative Osteosarcoma Study Group (COSS) were analyzed.
*Patients and methods:* Sixty-seven patients with a high-grade pelvic osteosarcoma were eligible for this analysis. Fifteen patients had primary metastases. All patients received chemotherapy according to COSS protocols. Thirty-eight patients underwent limb-sparing surgery, 12 patients underwent hemipelvectomy, and 17 patients did not undergo definitive surgery. Eleven patients received irradiation to the primary tumor site: four postoperatively and seven as the only form of local therapy.
*Results:* Local failure occurred in 47 of all 67 patients (70%) and in 31 of 50 patients (62%) who underwent definitive surgery. Five-year overall survival (OS) and progression-free survival rates were 27 and 19%, respectively. Large tumor size ($P = 0.0137$), primary metastases ($P = 0.0001$), and no or intralesional surgery ($P < 0.0001$) were poor prognostic factors. In 30 patients with no or intralesional surgery, 11 patients with radiotherapy had better OS than 19 patients without radiotherapy ($P = 0.0033$). Among the variables, primary metastasis, large tumor, no or intralesional surgery, no radiotherapy, existence of primary metastasis (relative risk [RR] = 3.456; $P = 0.0009$), surgical margin (intralesional or no surgical excision; RR = 5.619; $P < 0.0001$), and no radiotherapy (RR = 4.196; $P = 0.0059$) were independent poor prognostic factors.
*Conclusion:* An operative approach with wide or marginal margins improves local control and OS. If the surgical margin is intralesional or excision is impossible, additional radiotherapy has a positive influence on prognosis.

### The Guepar Prosthesis in Knee Reconstruction for Osteosarcoma in Children and Adolescents: 47 Cases After 15 Years Follow-Up

Eric Mascard, G. Missenard, P. Wicart, L. Brugières, C. Kalifa, J. Dubousset
*(Hospital Saint-Vincent de Paul, 82 Avenue Denfert-Rochereau, Paris 75014, France)*

*Introduction:* After resection of a bone tumor, reconstruction of the knee with a hinged prosthesis is the usual procedure. There is very few reports about long-term follow-up. The aim of our study was to analyse what elements were associated with increased failures rates and if rotating hinged, allograft-composite prostheses allowed improvement of late results.
*Patients and methods:* From 1981 to 1986, 47 patients, 24 girls and 23 boys, aged 9–18 (mean 13.7) had a resection of the knee, for osteosarcoma. Six patients had lung metastases at referral. All patients received pre- and post-operative chemotherapy, according to the T6 and T10 protocols. There were 34 distal femur and 13 proximal tibia resections. Reconstruction was always achieved by a custom made Guepar prosthesis, with polyethylene bushes, with cemented intramedullary stem. There were 19 prostheses with a rotating hinge mechanism. In proximal tibia resections the extensor mechanism was always reconstructed by a medial gastrocnemius flap. Results were assessed after a 11.5-year follow-up (4 months to 20 years). At last revision, 16 patients were deceased, and three were lost to follow-up before 15 year FU, leaving 28 patients. There was a $65.6 \pm 7\%$ overall survival of the patients at 15-year follow-up. Two patients had local recurrences. There were five deep infections. The main concern was mechanical complications, with eight stem fractures, five rotating stem fractures, 12 stem loosening requiring revision and 48 rebushing procedures. Including revisions, the 47 patients received a total of 80 prostheses. The survival probability of the prostheses was $42 \pm 7\%$ at 15-year follow-up. Five prostheses had complete revision to avoid repeated rebushing. The main factor influencing survival was the alignment of the limb after prosthetic implantation. When the limb had a varus mechanical alignment the survival was 28.5% at 10-year FU, and it was 82% in patients with a valgus or a neutral alignment. Rotating hinges and allograft composite prostheses did not show any improvement in prosthetic survival.
*Discussion and conclusion:* Knee hinge design and material are mandatory to avoid rebushing procedures. Some recent publications have shown improvement in survival of prosthesis with rotating hinge, but there is no paper comparing identical hinges with and without rotating mechanism. In our opinion, the improvements were mostly due to new hinge designs and the use of porous coated junction between host-bone and the prostheses. In our experience, composite allograft prostheses had more infections and showed no significant improvement of long-term follow-up. Avoiding implantation of the stem in varus is very important to allow long survival. Most of our mechanical complications occurred in prosthesis with a varus implantation. The main point is to choose properly the point of penetration of the stem into the tibia in distal femur resections, and the penetration into the femur when doing a proximal tibia resection.

### Transcutaneus Vertebroplasty in Combined Treatment of Patients with Tumor of the Spine

Mamed Aliev, B.I. Dolgushin, V.V. Teplyakov, A.K. Valiev, A.V. Kukushkin, I.A. Trofimov, O.M. Melusova
*(Department of Bone and Soft Tissue Tumors, N.N. Blokhin Cancer Center, 24 Kashirskoye Sh., Moscow 115478, Russia)*

Purpose is to propose and improve clinical importance of transcutaneus vertebroplasty as a part of combined treatment in patients with tumoral osteolytic lesions of the spine.
*Materials and methods:* A total of 21 patients were included in our trial. There were seven men, 14 women. Median age of the patients 46.5 (16–73). Histological type of primary tumors were: breast cancer 3, renal cancer 6, cancer of large intestine 1, cancer of the uterus 1, myeloma 3, gemangioma 5, giant cell tumor 1, tuberculosis 1. Localizations of the metastases in the spine were: four patients in the thoracic spine, 16 patients in the lumbar spine. Vertebroplasty was performed in 14 patients with back pain syndrome and seven patients with clinical manifestation of neurological deficits. Follow-up period was 18 months. The main indication for performing vertebroplasty was the risk of collapse of vertebral body and pain syndrome caused by instability in vertebral segment, destroyed by the tumor. The volume of infused bone cement ranged from 2 to 10 ml. Biopsy was taken in all cases, and the diagnosis was proved in 75%. All patients with malignant lesions received additional treatment, depending of histological type of tumor. Vertebroplasty was the first part in surgical treatment in three cases. These patients received conservative treatment (chemotherapy, immmunotherapy), and then laminectomy with transpedicular fixation were performed to them.
*Results:* A total of 71.4% (15 patients) of cases received good results; 19.0% (four patients), satisfactory results. In 9.6% of cases there were complications, consisting of increasing of pain syndrome. There were no cases of vertebral collapse during all the follow-up period even in patients with big cortical defects.
*Conclusion:* Percutaneus vertebroplasty is small invasive procedure which affords to decrease pain syndrome and the risk of the collapse of the vertebral body, especially in disseminated patients.

### Prediction of Local Response to Preoperative Treatment in Osteosarcoma

Gennady Machak, I. Solovyev, O. Zimina, A. Kuznetsova, O. Senko, M. Aliev
*(Department of Bone and Soft Tissue Tumors, N.N. Blokhin Cancer Center, 24 Kashirskoye Sh., Moscow 115478, Russia)*

*Background:* The response to induction treatment correlates with local and systemic control in osteosarcoma. Local response prediction could contribute to individualization of preoperative chemotherapy, surgery timing and type of surgery. The influence of several osteosarcoma characteristics on local response was retrospectively analyzed.
*Patients and methods:* Induction treatment consisted of DOX i.a.+36 Gy (protocol A, 1983–1986, 142 patients), three–four cycles of DOX or CDP i.a. (protocol NEO-1, 1986–1998, 278 patients), and four cycles of DOX i.v.+CDP i.a. (protocol NEO-2, 1999–2001, 26 patients). The cut point of response was 90% of tumor necrosis in protocol A and 100% in NEO protocols. The following variables were analyzed: sex, age, site, stage, duration of symptoms, relative (RTV) and absolute tumor volume (ATV), growth rate (GR), histological subtype, tumor vascularity, radiographic appearance, pathological fracture (PF), skip-metastases, the level of alkaline phosphatase (SAP), uptake of $^{99m}$Tc in arterial and bone phases, content of diploid, S, and G2+M tumor cells, and index of proliferation (IP). Logistic regression and pattern recognition technique were used for multivariate analysis.
*Results:* The response rate was 49% in protocol A, 6% in NEO-1, and 46% in NEO-2. The chondroblastic subtype was less responsive in protocol A (3/12) compared with other subtypes (43/79) ($P = 0.05$). A total of 86% of patients (6/7) with PF showed response compared with 47% (63/135) in the remaining patients ($P = 0.04$). In protocol NEO-1 the response was related to stage, RTV, ATV, GR and histological subtype. No responses were observed in the 39 stage IIIB patients. Response rate was 8% in tumors involving $< 1/3$ of bone and 2% in larger tumors, $P = 0.05$. The 12 patients with tumors $< 100$ ml showed a significantly higher rate of response than the 266 patients with larger lesions (33 vs. 5%, $P = 0.0001$). Response rate was 10% (16/158) in patients who had GR $< 140$ ml/month and 1% (1/120) in patients with GR $> 140$ ml/month, $P = 0.001$. No responses were seen in chondroblastic tumors, $P = 0.05$. Tumors containing >90% of diploid cells were resistant to NEO-1 chemotherapy (0/13 vs. 13/51, $P = 0.04$). Response rate was 35% (12/34) in tumors with < 12% of G2 + M cells and 4% (1/28) in patients with >12%, $P = 0.0023$. If the IP was < 25% the response rate was 38% (13/34). No responses (0/28) were observed if the IP was >25%, $P = 0.0002$. ATV and GR retained their significance in logistic regression ($P = 0.01$, $P = 0.02$, respectively). Despite the statistical significance ($\chi 2 = 18$, $P = 0.0001$), the prediction was difficult. All patients were classified as non-responders. Prediction was improved when DNA-analysis data were added ($\chi 2 = 27$, $P = 0.0000$). The poor response was predicted correctly in 90%; good in 67%. Overall exactness was 85%. Ploidy, G2 + M fraction and IP entered in pattern recognition algorithm. Poor response was predicted correctly in 76%; good in 85%. Overall exactness was 78%. Stage, RTV, ATV and histological subtype were no longer predictive when a more aggressive protocol NEO-2 was employed.
*Conclusions:* The prediction of local response at presentation remains difficult in osteosarcoma, particularly, when aggressive chemotherapy is administered. Prognostic factors were protocol-dependent. It was easily to predict the negative response than the positive one. DNA analysis of tumor cells may be useful in prognostic models. Additional factors are required to improve the prediction of local response at diagnosis.

### Endoprosthetic Reconstruction in Bone Tumors. Analysis of 245 Patients

Mamed Aliev, V. Sokolovsky, I. Solovyev, E. Kovalevsky, D. Nisichenko, A. Iliushin
*(Department of Bone and Soft Tissue Tumors, N.N. Blokhin Cancer Center, 24 Kashirskoye Sh., Moscow 115478, Russia)*

*Purpose:* To analyse the results of using of custom made endoprosthesis in patients with primary malignant or benign tumors of long bones.
*Materials and methods:* Between 1972 and 1992 we treated 245 patients who required resection of proximal and distal femur, proximal tibia, and proximal humerus. There were 129 males and 116 females, whose age ranged from 15 to 65 years. Histology was osteosarcoma in 113 patients (46%), chondrosarcoma in 28 (11%), malignant fibrous histiocytoma 12 (5%), giant cell tumor in 57 (23%), other malignant bone tumors 29 (12%) and other benign tumors (3%). Tumor site was distal femur in 189 patients, proximal tibia 31, proximal humerus 20, and proximal femur 5.
*Results:* Deep wound infection occurred in 29 patients (12%). There were 26 local recurrences (11%); 12 were managed by excision of the recurrent tumor, and 14 (6%) required amputation. Overall limb-salvage rate was 94%. There were 30 revisions (12%). A total of 23 patients with distal femur reconstruction, and seven with tumors of proximal tibia. Functional results were excellent in 61 patients (25%), good in 116 (57%), fair in 54 (22%), and poor in 14 (6%).
*Conclusion:* Resection of proximal and distal femur, proximal tibia, and proximal humerus followed by endoprosthetic reconstruction is the method of choice in the treatment of malignant and aggressive benign bone tumors.

### c-Kit Expression in Soft Tissue Sarcomas: Perspectives of Usage of Glivec

Beniamin Bokhyan, E. Stepanova, R. Keshta, R. Karapetyan, A. Ilushin, A. Baryshnikov, N. Petrovichev, M. Lichinitser, M. Aliev
*(Kashirskoe Sh. 24, Moscow 115478, Russia)*

c-Kit expression in different types of soft tissue sarcomas was studied using standard assessment criteria. The study included 83 patients treated in Cancer Research Center, Moscow. Histological verification of tumours was done using standard classification of soft tissue tumours. Immunohistochemical research was performed by the standard avidin–biotin technique. Monoclonal antibody to c-Kit used as primary antibodies: clone T595, dilution 1:20, Novocastra. We have found high c-Kit expression in 75% of gastrointestinal leiomyosarcomas. A total of 25% malignant peripheral nerve sheath tumour and 13% synovial sarcomas were also c-Kit positive. Thus, patients with synovial sarcomas and soft tissue sarcoma of neuroectodermic origin are perspective target for Glivec treatment. The additional studies for c-Kit assessment standardization in tumours are required.

### Her2/neu Overexpression in Chondrosarcoma

Teymuraz Kharatishvili, E. Stepanova, I. Solovyev, E. Kovalevsky, M.Aliev
*(Department of Bone and Soft Tissue Tumors, N.N. Blokhin Cancer Center, 24 Kashirskoye Sh., Moscow 115478, Russia)*

C-erbB-2 belongs to the epidermal growth factor receptor family, of which there are four known members, and has molecular homology to the epidermal growth factor receptor. Chondrosarcoma is resistant to traditional chemotherapy, and the search for new treatment combinations is necessary. The HER-2/neu overexpression was studied in chondrosarcoma. The study included 24 patients (II tumor grade was at 20 patients, III tumor grade – at four patients). All patients have a follow-up of more than 5 years. A total of 14 (58%) patients develop metastasis within 5 years. The research was performed on paraffin-embedded blocks using standard immunohistochemical method. As primary antibodies used a polyclonal antibody to HER-2/neu (A0458, Dako Corp.). Eight chondrosarcomas (33%) demonstrated cytoplasmic HER2/neu staining. None of the positive cases showed the membranous staining which is typical for breast cancer. Patients without development of metastases HER-2/neu overexpression was found in 10% cases, and the group with development of metastases, HER2/neu was found in 50% cases ($P = 0.07$). We found no clinical or pathological differences between the two groups of chondrosarcomas without or with HER2/neu overexpression. Patients with HER2/neu overexpression showed a lower disease-free survival. Median time to progression was 39.1 months in HER-2/neu− and 20.3 months in HER-2/neu+ patients ($P = 0.183$). These data suggest that HER2/erbB-2 is a perspective prognostic factor for chondrosarcoma patients. Clinical trials using Herceptest should be considered for the treatment of patients with chondrosarcoma.

### Methods of Microvascular Reconstructive Surgery in Treatment of Local Advanced Bone and Soft Tissue Tumours

Vladimir Sobolevskiy , T. Haratishvili, M. Kropotov, M. Aliev
*(Kashirskoe Sh. 24, Moscow 115478, Russia)*

The treatment of advanced cancer like T3–T4 or recurrent tumours results in wide defects. More often we use the conventional regional flaps for reconstruction. The indications for free tissue transfers in this cases is considered restrictively. The defect size, lack or local tissue compliance due to earlier operations, or president radiation can make microsurgical techniques necessary. Microvascular free flaps provide enough tissue to reconstruct those many complex defects with loss of osteo-cutaneo-muscular tissue. In the years 1998 through 2002 we performed 30 free flaps in our clinic. There were 15 females and

15 males, aged from 16 to 82 years. The flaps used were selected by the anatomical requirements of each defect. In 13 cases for reconstruction of bone defects we used a free vascularized fibular bone, for one patient, vascularized iliac crest. In one case we used combined scapular flap with scapular bone. In eight cases for fixing an osteal graft we used an Ilizarov device, in seven cases we used metal plates. The average expansion of substituted osteal defect was 16.5 cm (13–18 cm). In all cases the complete consolidation of a graft was scored. Periods of consolidation, from 2 months at substitution of defect of a ulnar bone, up to 6 months at a fracture of a graft on an tibia. The average time of consolidation was 4 months. For soft tissue reconstruction most common we use free toracodorsal flap (eight cases). In two cases we used free scapular flaps. When we need the thin graft for reconstruction, the radial forearm flap is more suitable. (five cases in our series). Thrombosis of microvascular anastomoses was observed in four cases. In all cases we have made revision of microvascular anastomosis. A total of 28 from 30 flaps completely survived. Reconstruction with microvascular free flaps allows more freedom in tumour excision, but it also seems to improve the patients postoperative function and postoperative morbidity. Prognosis of these patients with wide tumours is normally poor in the long run, but it seems that surgical efforts are indicated to attempt salvage and to improve the quality of life.

### Diagnostic Impact of MRI in Differential Diagnosis of Vertebral Metastases Versus Osteoporosis

Adam Mester, Sándor Forgács, Pál N. Kaposi, Kinga Karlinger, Katalin Köllő, Ernõ K. Makó
*(Pf.217., Budapest 1444, Hungary)*

Aim of presentation is a retrospective analysis and pictorial assay of MRI differential diagnosis of metastatic versus osteoporotic vertebral lesions.

*Methods and materials:* Series of 200 spine cases were evaluated in respect of metastatic versus porotic and other lesions with similar appearance. A low field whole body (0.3 T, Hitachi) scanner was used. Routine SE T1 and SE T2 sequences were completed with STIR and Gadoliniumdiethylenetriamine pentaacetic acid (Gd-DTPA) contrast administration in selected doubtful cases. Additional opposed phase GRE sequences were used as well.

*Results:* Increased T2 signal and decreased T1 signal, if diffusely distributed in the vertebral body, is characteristic (83%) to recent porotic compressions. In cases of non-compressed vertebral bodies with diffuse increased T2 signal increase this appearance had a predictive value (67%) of imminent compression fracture. T1-weighted opposed phase GRE sequences were specific and accurate in differentiation of focal and/or diffuse metastatic lesions versus porotic lesions (positive predictive value 88%) in both compressed and non-compressed vertebras.

*Conclusion:* Routine MRI (SE) can suggest lesions of metastatic and/or osteoporotic vertebral lesions. Additional STIR sequence, Gd-DTPA administration and opposed phase GRE imaging make the diagnosis more specific.

### Interferon-α as the Only Adjuvant Therapy in High-Grade Osteosarcoma: Long-Term Follow-Up from the Karolinska Hospital Studies

Christoph Müller, Sigbjørn Smeland, Gunnar Sæter, Henrik Bauer, Hans Strander
*(The Norwegian Radiumhospital, Oslo 0310, Norway)*

Based on proven activity against osteosarcoma cell lines, in 1971 a trial of adjuvant crude and semipurified human interferon alpha in osteosarcoma was initiated at the Karolinska Hospital. Patients were enrolled in subsequent phase II interferon protocols until 1990. In total, 92 consecutive patients were enrolled, of these 65 patients were < 40 years with high grade, non-metastatic osteosarcoma of the extremities. In this group, the median age was 15 years (range 5–32), and the m/f ratio was 1.6. In the period 1971–84 ($n = 52$, group 1) patients were treated with a dose of 3 MU daily for 1 month and then 3 times weekly for an additional 17 months (group 1). The dose was increased to 3 MU daily and the treatment duration extended to 3–5 years for the period 1985–1990 ($n = 13$, group 2). With a median follow-up of 12 years overall survival at 10 years was 40% for group 1 and 69% for group 2. A total of 85% of the survivors were in their first remission at last follow-up. Of the six patients that are long-time survivors after relapse none received chemotherapy. Thus the survival rates cannot be explained by second line chemotherapy salvage. Except for the expected constitutional side effects severe acute toxicity was not reported and long-time side effects were virtually absent. COSS-80 is the only randomized study addressing whether 'consolidation therapy' with interferon adds to the effect of combination chemotherapy in osteosarcoma. The study failed to show a benefit for the interferon group, which may be due to the type of interferon used (IFN-beta), underdosage and short treatment duration (5 MU twice weekly for 22 weeks). Interferon has antitumour effects by antiproliferative and proapoptotic pathways, as well as indirect effects by anti-angiogenesis and T-cell stimulation. The dose–response relationships for interferon shown in biological studies are supported by this update of the Karolinska series. Long-term treatment with interferon has significant constitutional side-effects and toxicity issues; the therapeutic ratio may however be improved by pegylation with polyethylene glycol and should be used in forthcoming studies of interferon in osteosarcoma.

### Acetabular Reconstruction for Metastatic Bone Disease

David Warnock, R.M. Tillman, R.J. Grimer
*(The Royal Orthopaedic Hospital Oncology Service, Bristol Road South, Northfield, Birmingham B32 2AP, United Kingdom)*

Metastatic bone disease resulting in acetabular destruction can provide the orthopaedic surgeon with the difficult challenge of achieving a stable reconstruction of the hip to provide pain relief and restoration of mobility. A review of 20 patients with metastatic disease requiring major acetabular reconstruction presenting to our orthopaedic oncology unit over a 5-year period was undertaken. This yielded 15 female and five male patients with a mean age of 59 years. The primary lesion was breast (eight cases), renal (three), prostate (two), myeloma (two) and others (five) with a solitary acetabular metastasis in 75% of cases. Eight patients had received radiotherapy to the region preoperatively. In all cases, diseased bone was macroscopically cleared from the pelvis and reconstruction performed by means of a Harrington procedure with threaded pins passed antegrade from the iliac crest (15 cases) or mesh and screws (five cases), all reinforced with cement around which a total hip arthroplasty was performed. Mean follow-up was 16 months. Complications were broken pin (one case), dislocation of femoral prosthesis (one) and deep venous thrombosis (one). Three patients died of their disease at a mean of 12 months from surgery. The remaining 17 patients continue to function at a satisfactory level with no patients having required revision surgery for loosening or deep infection. We believe that surgical reconstruction of the acetabulum is worthwhile and can provide these deserving patients with improvement in quality of life.

**Functional Results and Quality of Life After Treatment of Pelvic Tumors**

Christiane Hoffmann, Georg Gosheger, Carsten Gebert, Heribert Jürgens, Winfried Winkelmann
*(Department of Orthopedics, University of Muenster, Albert Schweitzer Straße 49, Muenster 48129, Germany)*

*Background:* In the past three decades, there have been substantial improvement in the disease outcome of patients with bone sarcomas. Quality of life and function, not just survival is central to outcomes analysis in musculoskeletal oncology. Primary bone sarcomas of the pelvis represent the most challenging problem in limb saving surgery. Aggressive surgery improve local control in pelvic tumors. However, little information exists about function and quality of life. The role of surgical resection remains controversial and the real outcome in terms of the impact of therapy is still unknown. The question of interest was: what is the impact of surgery on health related quality of life and function in long-term survivors with pelvic tumors?
*Methods:* Patients were excluded if they were less than 16 years old at the time of follow-up, if they were being treated for active metastatic disease and if there follow-up was less than 1 year from the end of treatment or if they could not read German. Quality of life was assessed using the European Organization for Research and Treatment Cancer Core Quality of life questionnaire (EORTC QLQ C 30) Function was assessed using the Musculoskeletal Tumor Society system developed by Enneking. Between January 1975 and December 2000, 141 patients with a primary bone tumor of the pelvis were treated in a single institution; 41 died of disease, five patients had progress of disease at time of follow-up, and one patient had a secondary malignancy (leukemia), 10 patients were lost to follow-up (six patients, lost to follow-up after 60 months, but who had no evidence of disease). A total of 19 were excluded. Six were treated for recently diagnosed metastases or progress of disease, one were less than 16 years at the time of follow-up. Six patients were excluded because of follow-up under 12 months since the end of treatment. Six patients did not read German. Functional evaluation and quality of life examination was possible in 71 patients. The median age was 30 years at the time of follow-up. The median time since the end of treatment was 67 months (range 12–267 months). Regarding surgical treatment, the surgical procedures included hip transposition in 18 patients, reconstruction with allo-/autograft in 11 patients, prosthesis in 10 patients, tumor resection without reconstruction in 25 patients and amputation in four (7) patients (+ three patients underwent external hemipelvectomie after primary surgery; two after local recurrence, one after deep infection of a pelvic prosthesis). In three patients with a Ewing's sarcoma the surgical intervention was only a second look biopsy after radiation therapy. In patients with pelvic tumors the mean functional status scored 18.2 (60.1%) of a maximum of 30 (range 5–30). The best functional results can be obtained in patients who can be treated with continuity resection. In our series, good functional results can be obtained In patients with tumor resection and allograft/or autograft reconstruction the mean functional result scored 61.2%. In patients with a tumor resection type II-III or type I-II, a endoprosthetic replacement as well as a hip transposition is possible for limb salvage surgery. When comparing these two groups significant better functional results were found in patients with hip transposition. The quality of life measurement in pelvic tumors scored: global health status (mean 66.1), physical functioning (mean 65), role functioning (mean 49.2), emotional functioning (mean 71.9), social functioning (mean 61.3), pain (mean 35.2), fatigue (mean 32.0). When asked for general evaluation of health patients with hip transposition and replacement with pelvic prosthesis reported being in good health ($P = 0.331$). Asking about limitations in doing hobby work or other daily activities, patients with hip transposition had significantly better results (mean hip transposition 54.6, mean prosthesis 23.3; $P = 0.016$). Also, in emotional functioning, patients with hip transposition fared better (mean hip transposition 75.5, mean pelvic prosthesis 65; $P = 0.324$). The surgical treatment of bone tumors located in the pelvis remains challenging and patients who may require such management should be assessed and treated only in a specialist center for orthopedic oncology. The hip transposition states a convincing surgical procedure in contrast to the used pelvic endoprosthetic replacement.

**When is a Lump a Sarcoma? – An Analysis of 1100 'Suspicious' Lumps**

Ashwin Kulkarni, R.J. Grimer, P.B. Pynsent, S.R. Carter, R.M. Tillman, A. Abudu
*(The Royal Orthopaedic Hospital, Bristol Road, Northfield, Birmingham B31 2AP, UK)*

*Purpose:* To see if existing guidelines for the early diagnosis of sarcomas can be improved.
*Method:* Data on 1100 patients referred to our unit with a lump suspicious of sarcoma was analyzed to try and identify clinical features more common in malignant than benign lumps. The following five items were analysed: size, history of increasing size, presence of pain, depth, age. For each of these items sensitivity, specificity, accuracy and weights of evidence were collected. ROC curves were used to identify the most sensitive cut off for continuous data.
*Results:* The best cut-off predicting malignancy for size was 8 cm and for age 53 years. The results of the tests are shown below.

| | Sensitivity | Specificity | WE +ve | WE −ve |
|---|---|---|---|---|
| Increase in size | 0.86 | 0.52 | 0.58 | −1.28 |
| Size >8 cm | 0.73 | 0.72 | 0.40 | −0.38 |
| Depth | 0.87 | 0.23 | 0.12 | −0.60 |
| Pain | 0.46 | 0.60 | 0.14 | −0.11 |
| Age >53 | 0.59 | 0.56 | 0.40 | −0.39 |

The weights of evidence (WE) are logs of the likelihood ratios and can be added and a probability then calculated. For example, a 36-year-old with a 10-cm, deep, painless lump that is increasing in size scores: $0.39 + 0.4 + 0.4 - 0.11 + 0.58 = 0.88$. This equates to a risk of the lump being malignant of 70%.
*Conclusion:* This analysis shows that increase in size is the strongest predictor of malignancy/benignancy followed by age >53 and size >8 cm. This data can help formulate strategies for earlier detection of soft tissue sarcomas.

**Infection Rate in Limb Salvage Surgery and Antibiotic Prophylaxis – Survey of European Oncology Centres**

Ashwin Kulkarni, J. Gray, R. Grimer, P. Pynsent, S. Carter, R. Tillman, A. Abudu
*(The Royal Orthopaedic Hospital, Bristol Road, Northfield, Birmingham B31 2AP, UK)*

*Aim:* Infection in limb salvage surgery (LSS) remains a major complication with disastrous result. The current practice of prevention of infection and its effectiveness in LSS in European Oncological centres was surveyed to determine the variations and common practice.
*Method:* An email questionnaire was sent out to orthopaedic oncology surgeons across the Europe to assess their infection prevention methods and its success in LSS. Results were analysed.
*Results:* All surgeons use perioperative antibiotic. The common choices are third-generation cephalosporins and aminoglycosides.

Nearly 50% surgeons use antibiotic in bone cement. All surgeons use drains and nearly all use plastic adhesive cover introperatively. Acceptable limits of blood counts in chemotherapy patients vary but are within a fairy small range. Deep infection rate varies widely (2–10%). The combination of patient status, antibiotic use and available resources are important factors affecting infection rate in LSS.
*Conclusion:* There is considerable variation in current practice between individual centres and surgeons. There is no proof suggesting an optimal regime. There remains a major uncontrollable factor of patient susceptibility or resistance to infection.

**Genomic Imbalances in Synovial Sarcoma Evaluated by Comparative Genomic Hybridization**

Yasuko Nakagawa, Toshifumi Ozaki
*(2-5-1shikata cho , Okayama City, Okayama Prefecture 700-8556, Japan)*

*Introduction:* More than 95% of synovial sarcomas have a translocation t(X;18)(p11.2;q11.2): one type of chimeric fusion gene, SYT-SSX2 is reported to be associated with patient prognosis and histological subtypes. However, chromosomal instabilities in synovial sarcomas have not clarified. In the current study, the frequent chromosomal aberrations in synovial sarcomas were evaluated and the correlation between chromosomal imbalance and several clinico-pathological factors are analyzed. The results of chromosomal instabilities in a Japanese collection of tumors were compared with those of a German collection.
*Materials and methods:* Comparative genomic hybridization (CGH) was used to detect chromosomal aberrations in fresh-frozen tumors. Fourteen primary and untreated tumor tissues from the Japanese collection were available for this study. Six tumors were diagnosed as biphasic and eight were monophasic type. Eight tumors expressed the SYT-SSX1 (biphasic, five; monophasic, three) fusion transcript, and six tumors had the SYT-SSX2 (biphasic, one; monophasic, five) fusion transcript. In the German group, six tumors were monophasic type and three tumors were biphasic type. Seven tumors showed the SYT-SSX1, and two showed SYT-SSX2 fusion transcript.
*Results:* In 14 Japanese tumors, genetic aberrations were detected in 10 of the 14 (71%) cases. Gains (average 4.4) were more frequent than losses (0.9). The frequent gains included 12q15 (five cases), 12q22 (five cases), 12q24.3 (five cases), 12q14 (four cases), 12p (four cases), 8q21.3 (four cases), and 21q22 (four cases). The most frequent losses included 3p14 (two cases) and 6q (two cases). High-level gain was observed at 12q15 (four cases), 12q22 (four cases), 12q24.3 (four cases), and 8q (three cases). Eight tumors with SYT-SSX1 fusion transcript and six with SYT-SSX2 had an average of 3.9 and 5.0 aberrations, respectively. There were few aberrations specific to histological subtype excluding 12q15 or 12q22; monophasic tumors had gains of 12q15 ($P = 0.021$) and 12q22 ($P = 0.021$) more frequently than biphasic tumors. Average number of aberrations (6.8) and total number of high-level gains (25) in the monophasic tumors were larger than those (1.2) (3) in the biphasic tumors, respectively. In nine tumors of German group, genetic aberrations were detected in six of nine (67%) tumors. Gains (average 6.1) were more frequent than losses (4.4). Frequent gains included 13q21–q22 (four cases) and 18q22-qter (four cases). Frequent losses included 1p33-pter (five cases), 16p (four cases), 17p (four cases), and 19 (four cases). High-level gain was observed at 13q21-q22 (one case). Seven tumors with SYT-SSX1 fusion transcript and two with SYT-SSX2 had an average of 12.7 and 3.0 aberrations, respectively. A comparative analysis revealed no significant difference of total number of aberrations, gains, and losses between Japanese and German collections of tumors. Although sample sizes are too small to support population statements, two distinct patterns emerged. Gains of 3q32, 4q26-qter, 5p, 5q14–q23, and 11p and losses of 1p33-pter, 3p21, 11q12, 16p, 17p, and 22q were more frequent in the German collection than in the Japanese collection. Gains of 12q15 and 12q22 were more frequent in the Japanese collection than in the German collection.
*Conclusions:* CGH results were not correlated with the chimeric fusion gene type; however, monophasic tumors had chromosomal aberrations more frequently than biphasic tumors. Tumors in the Japanese collection had gains of 12q15 and 12q22 more frequently than those in German collection. This region is associated with subtype of synovial sarcomas; genes in these regions may be important for identification of subtypes of synovial sarcoma.

**Epithelioid Sarcoma: Clinical Behaviour of a Rare Soft Tissue Sarcoma**

Dario Baratti, A. Gronchi, P. Casali, R. Bertulli, R. Casirani, L. Lozza, P. Olmi, M. Santinami
*(Via G. Venezian 1, Milan 20133, Italy)*

Epitheliod sarcoma (ES) is a rare subtype of soft tissue sarcoma (STS). Distinctive clinical features are the tendency to be multifocal at presentation and to spread locally at recurrence, with an higher rate of lymph nodes involvement and in-transit metastases.
*Patients and methods:* Forty-five consecutive patients with ES were observed at our institution from June 1985 to June 2002. Thirty-two were male and 13 female; median age at the time of first diagnosis was 29 years (range 4–77). Site of first presentation was the hand in 19 cases, the upper limb in eight, the foot in two, the lower limb in 11 and the trunk in five. Fifteen out of 45 patients referred after a median of three consecutive relapses (range 1–5), developing multifocal recurrences in four cases, loco-regional nodal involvement in two and distant metastasis in one. Two patients underwent amputative surgery before our observation. Primary treatment performed in our department was wide en-bloc excision, with the aim to obtain adequate margin of uninvolved tissue in all directions, and to spare limbs and limbs function as much as possible. Adjuvant RT was administered to high risk patients in a postoperative fashion.
*Results:* ES presented as small superficial nodules in 27 cases and this feature was prevalent in distal localization (19/27), while deep-seated larger masses were more common in the proximal extremities or in the trunk (17/18). Surgical treatments performed were: wide excision in 23 patients, re-excision in 12, digit or ray amputation in five, major amputation in three. Surgical margins were rated as wide in 27 patients and marginal in eight. No residual tumor was found in 10 out of 12 patients who underwent re-excision. Adjuvant therapies were the following: postoperative RT in 16 patients, preoperative limb perfusion in nine, preoperative conventional CT in seven and postoperative CT in seven. Median follow-up of the series was 58 months (range 4–316). Actuarial overall survival at 5 years was 72.7%; disease-free survival at the same interval was 48.2%. Following treatment at our institution, from one to four consecutive relapses (median 2) occurred in 21 patients. Lymph nodal involvement was observed in two patients at the time of first diagnosis, and it developed in three and eight patients during the clinical course, respectively, before and after our observation. Therefore, the crude rate of loco-regional involvement was 13/45 (28.9%). Distant metastases were detected at the time of first presentation in one case, developed as first recurrence in four and was subsequent to nodal involvement in 10. Most common site of distant spread was the lung. All but two patients with metastatic disease are presently died. Nine more amputations were needed to treat local recurrence, thus a total number of 21 such procedures were performed in 16 patients. Relapse occurred in 10 out of 30 (33.3%) patients treated by primitive neoplasm and in 11 out 15 (73.3%) treated for recurrent tumor; difference was statistically significant (odds ratio = 5.50; 95% CI = 1.393–21.7).

*Conclusions:* Epithelioid sarcoma is a rare neoplasm, accounting for less than 1% of all the STS observed during the period of the study. The present study showed a great propensity to recur repeatedly and an high rate of nodal involvement was confirmed. Lack of local control leaded to an increase in amputation rate. Adequacy of treatment was the most relevant prognostic factor, since patients referred for recurrent disease, after repeated attempts of local control, tend to show a worse outcome than patients undergoing primary treatment in our Department.

**Isolated Bone Metastases of Unknown Primary Site: Diagnostics and Treatment**

Igor Komarov, D.V. Komov, E.A. Bogush
*(Surgical Department of Diagnostics, Blokhin Cancer Research Center, Kashirskoye Shosse 24, Moscow 115478, Russia)*

Analysis of diagnostic evaluation and treatment of 99 patients with bone metastases of unknown primary site (totally 899 patients with tumors of unknown primary) allows optimization of diagnostic procedure and the optimal algorithm of diagnostic search was finally formulated. (I) A thorough medical history and physical examination to determine the performance status. No further diagnostic procedures are necessary for incurable patients. (II) To determine the extent of metastatic disease according to the results of chest X-ray, ultrasonography of abdomen, retroperitoneum and pelvis, bone and lymph nodes scan, brain CT. If occult primary site is occasionally identified the patient should receive specific treatment. The other patients are divided into two subgroups: with isolated skeletal metastases or synchronous involvement of several organs or systems. (III) Morphological evaluation, including immunohistochemistry of biopsy specimens. No further diagnostic procedures are necessary for patients with no evidence of malignant tumor. If any neoplasm of hematopoietic system is identified the patient should receive specific treatment. Patients with adenocarcinoma, poorly differentiated carcinoma and rare malignant lesions need further evaluation. (IV) In patients with isolated bone lesions it is necessary to continue the search for primary tumor which has predominantly skeletal metastases: prostate ultrasonography, serum level of prostate-specific antigen, bronchoscopy, mammography, thyroid gland scan. In patients with morphologically diagnosed 'malignant tumor' serum levels of HCG and α-fetoprotein, additional immunological evaluation of biopsy specimen should be performed to exclude germ-cell tumor or lymphoproliferative disease. If occult primary site is occasionally identified the patient should receive specific treatment, if not combined chemo- and radiation therapy, if possible, surgical excision of metastatic lesions. Sometimes the identification of primary tumor and then specific antitumor treatment are possible during the follow-up of these patients.

**How to Inform the New Sarcoma Patient?**

Forni Cristiana, L. Loro, T. Mazzei, C. Beghelli, M. Tremosini, A. Biolchini, A. Triggiani, C. Raspanti
*(Via Pupilli 1 Istituto Ortopedico Rizzoli, Bologna 40136, Italy)*

When a new patient comes to a Chemotherapy ward, there is a lot of information he/she needs to know in a very short time. Nurses play a key role in this field. During the last 10 years the nursing team of the Chemotherapy Service at Rizzoli Institute have written various educational booklets on this topic. We started with producing a patient's booklet on Chemotherapy and the main role and organization of our ward to be given at each new patient during the first admission in our ward. After several months we carried out a survey amongst patients at the end of their treatment. We wanted to check whether the information given at the beginning of their treatment had been of any use. The survey results gave us useful suggestions for our further information booklets and leaflets:

q What Chemotherapy is and the role of nurses, doctors and patients;
q Specific information on drugs used: Methotrexate, Vincristine, Ifosfamide, Cisplatin and Adriamicina;
q How the school service work in our world;
q What GCSF is, the practical procedures of using them and their side effect;
q The differences between Peripheral and Central Venous Catheter;
q The differences between intravenous and intrarterial therapy.

The up-dating of our booklets and leaflets is an ongoing process. In this paper we would like to discuss and compare methodologies and materials with colleagues who wok in the same field.

**Magnetic Resonance (MR) Angiography in Bone and Soft Tissue Tumors**

Toshifumi Ozaki, H. Doi, Y. Kawakami, A. Ono, A. Kawai, T. Kunisada, Y. Morimoto, H. Inoue
*(Department of Orthopaedic Surgery, Okayama University Postgraduate School of Medicine and Dentistry, Okayama 700-8558, Japan)*

*Introduction:* Evaluation of local blood flow is important for surgical planning of bone and soft tissue tumors. Digital subtraction angiography has been used for estimation of blood flow in/around the tumors; however, this method is associated with intervention and irradiation. The authors applied a new MR angiography technique that does not use contrast enhancement for bone and soft tissue tumors.
*Methods:* MR angiography was performed in 97 patients with bone and soft tissue tumors. There were 43 female and 53 male. Forty-six bone tumors included 11 osteosarcomas, five enchondromas, two metastatic tumors, two chondrosarcomas, and 26 other tumors. The 51 soft tissue tumors included five malignant fibrous histiocytomas (MFHs), six schwannomas, three synovial sarcomas, two malignant peripheral nerve sheath tumors (MPNSTs), and 37 other tumors. There were 60 benign tumors and 37 malignant tumors. Fifty-four tumors were located in the lower extremity, 23 in the upper extremity, and 20 in the trunk. MR images were obtained with a 0.5 T MR scanner (FLEXART Hyper, Toshiba, Japan). Half-Fourier FSE (FASE) method with ECG-gating was used. The visualization of tumor and local vessels in and around the tumor was evaluated.
*Results:* The following arteries in the pelvis and the lower extremity could be clearly detected: the common iliac a., int. and ext. iliac a., femoral a., prof. femoris a., lat. circumflex a., ant. tibial a., post. tibial a., and fibular a. In many cases, a venous tumor thrombus and a distortion or encasement of vessels by tumors were observed. In the upper extremity, large arteries such as the subclavian a. were detected; however, vessels located in the more peripheral part to the elbow could not be evaluated. Discrimination between benign and malignant tumors were difficult in more than 50% of cases.
*Discussion:* The authors applied a new MR angiography technique in clinical cases of bone and soft tissue tumors without administration of contrast medium, or intervention, or irradiation. In the current study, the condition of vessels in and around tumors in the lower extremity and the pelvic area could be evaluated in a short time without intervention. The technique was less effective for vessels in the upper extremity or peripheral areas of extremities. Further improvement of the technique is necessary to apply it to bone and soft tissue tumor of the upper extremity, trunk, and peripheral areas of the extremities.

**Silver-Coated Tumorendoprostheses – In Vitro Experiments for Evaluation of Osteointegrative Properties**

Georg Gosheger[1], H. Ahrens[1], H. Burger[2], W. Winkelmann[1], A. Battmann[3]
*([1]Department of Orthopaedics, University of Muenster, Albert-Schweitzer 33, Muenster 48149, Germany, [2]Department of Pathology, University of Muenster, Germany, [3]Department of Pathology, University of Giessen, Germany)*

*Introduction:* In a previous experimental study (Gosheger *et al.*, 2002, *Sarcoma*) silver-coated MUTARS tumour endoprostheses significantly reduced the infection rate in comparison to titanium tumour endoprostheses (7 vs. 47%; $P<0.05$, $\chi^2$-test). Side effects could be excluded. Only the parts replacing the resected bone have been coated with silver in this previous study. The stems were not coated with silver.
*Problem:* Coating of intraosseous prosthetic stems as well could contribute to a further reduction of the infection rates. Osteointegrative properties of a silver coated stem are unknown so far. Aim of this study was to analyse the osteointegrative effects of a silver coating on osteoblasts in in vitro cell culture.
*Material and methods:* Using established osteosarcoma cell lines HOS 58 and SAOS in in vitro cell cultures, high cell proliferation was guaranteed. Proliferation of cells (MTT-Assay), activity of alcalic phosphatase (PNPP-Assay) and synthesis of osteocalcin (ELISA) have been analysed in both cell lines in six series. Next to a control series alkaline phosphatase and osteocalcin have been tested in dependence of silver or titanium addition in a concentration of 5–25 mg.
*Results:* A significantly increased activity of the alkaline phosphatase could be noticed regarding to the middle activity of the alkaline phosphatase at a silver addition of 5 or 10 mg in comparison with the titanium group: (HOS 58: 210 U/100 mg and 210 U/mg versus 190 U/100 mg, 130 U/100 mg). At higher silver addition degraded results of the alkaline could be noticed. SAOS cell lines did not confirm this ratio. Measuring the synthesis of osteocalcin there was no significant difference between silver and titanium.
*Discussion/conclusion:* The results of the in vitro study show increased activity of alkaline phosphatase in low silver concentrations in comparison with titanium addition as a sign of a positive osteointegrative property of a silver coating. Further examinations should be carried out because of these positive results concerning osteointegrative properties and the known antiinfective property of the silver coating.

**Trapezius Transfer and Latissimus Dorsi Transfer in Endoprosthetic Replacement of the Humerus in Three Patients**

Georg Gosheger, H. Ahrens, J. Hardes, C. Gebert, W. Winkelmann
*(Department of Orthopaedics, University of Muenster, Albert-Schweitzer 33, Muenster 48149, Germany)*

For the improvement of the functional outcome after proximal/total humerus replacement, we combined the surgical procedures described by Bateman and Gerber. In three patients after wide tumor resection, the endoprosthetic replacement with a modular tumor endoprosthesis (MUTARSŇ-System) was performed. In addition to a capsular and muscular reconstruction, using the trevira tube, a trapezius transfer onto the trevira tube in combination with a latissimus dorsi transfer onto the trevira tube was performed. The patients were immobilized for 6 weeks after surgery with an abductor cast. After a follow-up of 1 year there was no significant improvement of the shoulder function in comparison with patients, who did not undergo the combined muscle transfer. The reason for not improving the shoulder function with the combined procedure could be the missing rotator cuff. The necessity of a properly functioning rotator cuff seems to be very important causing compression of the articular surfaces so that a transposed trapezius muscle could function as an abductor in a stable joint. The Trevira tube as a static stabilizer could not provide sufficient stability of the joint for the abductory function of the transposed trapezius. Therefore the functional results do not justify two sperated approaches and a prolonged operation time. Further investigations should concentrate on the design of endoprostheses in order to improve the functional outcome after proximal/total humerus replacement.

**The Stener Method of Resection-Arthrodesis of the Humero-Scapular Joint Revisited; Excellent Results At 15–20 Years Follow-Up**

Björn Gunterberg
*(Dept. of Orthop., Sahlgren University Hospital, Göteborg SE-413 45, Sweden)*

In 1982 Bertil Stener introduced a method of resection-arthrodesis of the humero-scapular joint after removal of the proximal humerus for tumor. The results in those three patients with the longest follow-up were presented at the EMSOS meeting in 1996. The aim of this communication is to reassess the results 6 years later.
*Method:* After resection of the proximal humerus the defect (8–14 cm) was reconstructed with a non-vascularized fibular graft and an iliac graft. The shaft of the fibular graft was inserted into the medullary canal of the humerus and the articular cartilage of the fibula and the scapula was removed. The fibular head was fixed to the scapula with a steel wire. The iliac graft was interlocked between the humeral shaft and the coracoid process. The functional results according to the MSTS-system were recorded.
*Results:* Patient 1. A 15-year-old boy with chondrosarcoma. Result av 20 years 28/30 – 93%. The patient has successfully participated three times in the Vasa cross-country skiing competition (90 km). Patient 2. A 21-year-old woman with giant-cell tumor. Result after 19 years 27/30 – 90%. The patient is almost unrestricted in her activities. Patient 3. A 25-year-old man with aneurysmal bone cyst. Result after 15 years 27/30 – 90%. The patient works full-time as an electrician. He has rebuilt his house all by himself.
*Conclusion:* Such good long-term functional results as those presented above are rarely achieved after prosthetic replacement. This conclusion is supported by the studies on shoulder resection and reconstruction that were presented at the EMSOS meeting in 2002.

**Stener's Reconstruction of the Spine after Total Spondylektomy. Results At 22–34 Years Follow-Up**

Björn Gunterberg
*(Dept. of Orthopaedics, Sahlgren University Hospital, Göteborg SE-413 45, Sweden)*

Bertil Stener pioneered 'total spondylectomy' – an expression he coined in 1971 – for complete removal of primary tumors of the spine. The purpose of this study is to assess the results of his ingenious methods of reconstruction of the spine in those four patients, with the longest follow-up.
*Methods:* The resections aimed at complete removal of the tumors including the whole vertebra(e). The reconstruction principle was based on firm interlocking and compression of autologous corticocancellous bone blocs between the adjoining vertebral bodies and posterior instrumentation and fusion. The metallic implants available were originally meant for less demanding purposes but were applied in a way to give as much stability and compression as possible. Body braces were worn for 8–12 months postoperatively. The MSTS-scores (lower limb) were assessed from files (patient 2) and interviews.
*Results:* Patient 1. A 26-year-old female with giant-cell tumor of T11–L1, causing paraparesis. Score at 34 years, 26/30 – 87%. Walks cross-country several kms, no supports, heavy work. Patient 2. A 33-year-old female with giant-cell tumor of T11. Score at 27 years, 25/30 – 83%. House-wife, not very active, bicycles occasionally. Patient 3. A 49-year-old man with chondrosarcoma of T7. Score at 23 years, 21/30 – 70%. Mild spasticity post-operatively. Non-tumor-related death. Patient 4. A 23-year-old female with giant-cell tumor of L4. Score at 22 years, 26/30 – 87%. Full-time work, occupational therapist, regular work-out exercises.
*Discussion:* Lasting, stable reconstruction of the spine after total spondylectomy requires solid bony fusion anteriorly and posteriorly. This was achieved with autologous bone grafts, anteriorly tricortical blocs, posteriorly varying shapes and kinds. The supporting metallic implants (AO-plates, wires, screws, Meurig-William plates, Harrington rods, Dwyer compression wires) were primitive compared to what is available today. Because of the questionable strength of the implants and their anchorage to the spine the patients were kept in bedrest for 2–6 months and wore plaster jackets for 8–12 months. These periods can be considerably shortened with the new generation of spinal instrumentation. Solid fusion occurred in all patients. The long-term results (22–34 years) are surprisingly good. Thus, total spondylectomy with skeletal reconstruction is compatible with a long, physically active life.

### Complications after Modular Endoprosthetic Replacement in Patients with a Large Bone Defect of the Upper and Lower Extremity

Jendrik Hardes, Christiane Hoffmann, Arne Streitbürger, Carsten Gebert, Winfried Winkelmann, Georg Gosheger
*(Department of Orthopaedics, University of Münster Hospital and Clinics, Albert-Schweitzer-Str. 33, 48149 Münster, Germany)*

*Objective:* The objective of this study was to determine the complications of endoprosthetic replacement of the upper and lower extremity at our institution.
*Methods:* Between 1992 and 2002, endoprosthetic replacement with a MutarsR endoprostheses was performed on 265 patients after resection of a large bone segment. The mean age was 37.7 years (range, 7–89). A total of 117 patients were female and 148 patients were male. The average follow-up was 27.0 months (range, 3–106 months). Diagnosis was osteosarcoma in 115, Ewing-sarcoma in 31, chondrosarcoma in 40, osseous leiomyosarcoma/malignant fibrous histiocytoma in 15, metastasis in 25, lypmphoma in three and hemangioendothelioma in one patient. Seven patients had a soft tissue sarcoma with bone involvement. Benignant tumours or tumour like lesions occurred in four patients. In 21 patients the operation was performed in the case of revision surgery after failed endoprosthetic replacement of the hip or knee joint with a large bone defect. Two patients had a comminuted fracture of the distal femur and one patient had an osteomyelitis of the distal femur. In 39 patients a proximal humeral replacement was performed, three patients received a distal humeral replacement, four patients a total humeral replacement and one patient a diaphyseal implant. Proximal femoral replacement was performed in 64 patients, distal femoral replacement in 96 and total femoral replacement in 14 patients. In three patients a diaphyseal femoral implant was used. In 36 patients a proximal tibia replacement was performed. In five patients a stump lengthening procedure was done.
*Results:* The most common complication was infection in 28 patients (10.6%), predominantly in proximal femoral replacement (20.3%), distal femoral replacement (8.3%) and proximal tibial replacement (8.3%). Aseptic loosening was noted in 25 patients (9.4%) with the most common location in the distal femur in 16.7% of cases. Shoulder dislocation happened in 2.3% of patients with a proximal or total humeral replacement, hip dislocation in 9.4% of patients with a proximal femoral or total femoral replacement. Wear of the load-bearing surfaces was noted in 11 patients with a distal femoral or proximal tibial replacement (8.3%). Fracture of a prosthetic femoral stem only happened in one patient. Soft tissue complications developed in 19 patients (7.2%). A total of 147 revision operations were necessary in 265 patients; 51 operations were done because of mechanical complications; 84 operations because of soft tissue problems and infection; and 12 operations because of local recurrence. At follow-up, 238 patients had an endoprosthesis (89.8%). During follow-up, 12 patients (4.5%) had limb amputations, four patients a rotationplasty, two patients an arthrodesis of the knee joint, two patients a stump lengthening procedure and seven patients received an antibiotic loaded cement spacer, which was not removed because of an advanced case. The cause for amputation was local recurrence or metastasis in seven patients (58.3%) and infection or wound healing problems in four patients (33.3%). Rotationplasty was indicated in all cases because of infection.
*Conclusion:* Limb salvage with endoprosthesis is in most cases successful. The most common reasons for amputation or rotationplasty are recurrence and infection. Mechanical failures do not lead to an amputation and are treated by revision surgery.

### Long-Term Survival in High Grade Axial Osteosarcoma with Bone and Lung Metastases Treated with Chemotherapy Only

A. Brach del Prever[1], F. Fagioli[1], M. Berta[1], A. Palmero[1], G. Di Rosa[2], F. Bertoni[3], S. Ferrari[3], M. Mercuri[3]
*([1]Department of Pediatric Oncology, University of Turin, [2]Service of Pediatric Radiology, OIRM-S, Anna, Turin, [3]Department of Musculoskeletal Tumors, Istituti Ortopedici Rizzoli, Bologna, Italy, c/o OIRM-S.Anna Piazza Polonia 94, Torino 10126, Italy)*

Over the past few years the outcome of patients with high grade osteosarcoma of the extremities has improved dramatically, with survival rates rising from 15 up to 70%. This, at present, could be attributed to the development of new surgical techniques and the introduction of the neo-adjuvant chemotherapy. By contrast, patients with axial localization, multiple metastases at diagnosis and non-viable surgical approach have a poor prognosis. In May 1997 we observed a 2 year 10 month old boy with back pain lasting 2–3 months and progressive weakness in the legs in the 2 weeks leading up to his arrival in our Centre. The spinal CT scan and MRI showed a lytic lesion of the fifth lumbar vertebra with important soft tissues involvement and secondary osteolytic lesion of the fifth thoracic vertebra. The lung CT scan evidenced three bilateral metastatic nodules. Open biopsy of primary lesion, performed by the Rizzoli Institute, demonstrated an undifferentiated high grade osteosarcoma. The patient was treated according to the Italian-Scandinavian ISG-SSG II protocol, proposed in the attempt to improve cure rate in pelvic and metastatic osteosarcoma. This includes a preoperative chemotherapy with methotrexate $12\,g/m^2$ on days 0 and 42, cisplatin $120\,mg/m^2$ i.v. continuous infusion for 48 h, followed by adriamycin $75\,mg/m^2$ i.v. continuous infusion for 24 h from days 7 and 49, ifosfamide $15\,g/m^2$ in 5-day i.v. continuous infusion from days 28 and 70. Peripheral blood stem cell (PBSC) collection was performed after the second cycle of Ifosfamide, obtaining $10.25 \times 10^6$/kg CD34+ cells in three aphereses. We observed a rapid clinical improvement with the disappearance of back pain and the mass. Disease reevaluation showed a significant reduction of secondary soft tissue involvement and the disappearance of lung nodules. The chemotherapy that followed the reevaluation, included two cycles of adriamycin 90 mg/m$^2$, intercalated by one cycle of cyclophosphamide 4000 mg/m$^2$ + etoposide 600 mg/m$^2$ and two consecutive cycles of high dose carboplatin 1500 mg/m$^2$ + etoposide 1800 mg/m$^2$ in 4 days with PBSC rescue. Considering the unfeasibility of adequate surgery and the good response to chemotherapy, we decided to continue treatment with three cycles of methotrexate 12 g/m$^2$, followed by etoposide 50 mg/m$^2$ per day orally for 21 days every month, for a total of 12 cycles. Chemotherapy was well tolerated with only mild toxicity. At present the patient is in very good general condition, the lung CT scan and bone scintigraphy are normal, the spinal MRI is unmodified with no evidence of relapse at 69 months from diagnosis and 50 months from the end of treatment.

**Treatment of High-Risk Osteosarcoma. Preliminary Results of The Italian-Scandinavian ISG-SSG II Protocol**

A. Brach del Prever[1], S. Smeland[2], A. Tienghi[1], T. Bohling[2], M. Aglietta[1], O. Brosjo[2], G. Bernini[1], T. Wiebe[2], F. Fagioli[1], KS.Hall[2], S. Ferrari[1], T. Alvegard[2]

*(For the [1]Italian Sarcoma Group and [2]Scandinavian Sarcoma Group, c/o OIRM-S, Anna Piazza Polonia 94, Torino 10126, Italy)*

In the attempt to improve cure rate in pelvic and primary metastatic osteosarcoma, an Italian-Scandinavian research protocol adding high dose chemotherapy (HDCT) with peripheral blood stem cell (PBSC) support to conventional poly-agent chemotherapy has been tried. Preoperative chemotherapy consisted of methotrexate 12 g/m$^2$ on days 0 and 42, cisplatin 120 mg/m$^2$ i.v. continuous infusion in 48 h followed by adriamycin (ADM) 75 mg/m$^2$ i.v. continuous infusion in 24 h from days 7 and 49, ifosfamide 15 g/m$^2$ in a 5-day continuous infusion from days 28 and 70. After surgery, patients received two cycles of ADM 90 mg/m$^2$ intercalated by one cycle of cyclophosphamide 4000 mg/m$^2$ + etoposide 600 mg/m$^2$, and two consecutive cycles of HDCT containing etoposide 1800 mg/m$^2$ + carboplatin 1500 mg/m$^2$ (HDEC) over 4 days with PBSC rescue and G-CSF. From May 1996 to June 2002, 47 patients, 26 males and 21 females with a median age of 17 years (range 2–38) entered the study. Thirty-seven patients presented with metastatic disease at diagnosis (primary site: femur in 18, humerus in nine, tibia in five, pelvis in three and vertebrae in two) and 10 with pelvic localization. For the patients with primary metastatic disease all had lung metastases (more than two nodules in 27 cases) and in addition three had concurrent lymph node and one bone metastases. Resection of primary tumor was performed in 35 cases, amputation in three; 27 patients underwent lung metastasectomy. A median of two aphereses were performed (range 1–7) with a median collection of $5.7 \times 10^6$ CD34+/kg; two patients needed additional bone marrow harvest. Twenty patients received two cycles of HDEC and eight patients one cycle for a total of 48 evaluable cycles. Conditioning regimen was well tolerated in all patients with a median time to neutrophils $>0.5 \times 10^9$/l and platelets $>25 \times 10^9$/l of 10 days. Except for expected haematological toxicity and mucositis, no grade III or IV was reported after HDEC. With a median follow-up of 31 months (range 3–70 months), 17 patients are alive in first complete remission (C.R.), five in second or more C.R., three are alive with disease, two are alive in treatment and 20 dead of disease. The projected overall and the event-free survival at 36 months from diagnosis are 41 and 28%, respectively. These preliminary data show that the treatment is feasible with most patients receiving intended therapy. At present, even if the number of cases and the follow-up are limited, this approach seems to be promising in the treatment of high risk osteosarcoma.

**Three-Level En-Bloc Spondylectomy in a Desmoplastic Fibroma of the Thoracic Spine – a Case Report**

Jendrik Hardes, Georg Gosheger, Henry Halm, Winfried Winkelmann, Ulf Liljenqvist

*(Department of Orthopaedics, University of Muenster, 48149 Muenster, Germany*

*Study design:* A case report describing a patient with a desmoplastic fibroma (DF) of the thoracic spine treated by a three-level en-bloc spondylectomy.

*Objective:* To present a rare case of DF of the spine and emphasize the importance of an at least marginal resection of this tumor entity.

*Summary of background data:* DF is a rare tumor with approximately 220 cases reported in the literature predominantely occurring in patients younger than 30 years of age. Predeliction sides are the mandible and the meta-diaphyses of long bones. A localization in the spine is reported in only a few cases. DF has a high tendency of local recurrence especially after intralesional resection.

*Methods:* The reported case is that of a 14-year-old girl with a DF of the ninth, tenth and eleventh vertebrae. After confirmation of the diagnosis by CT-guided biopsy, a three-level en-bloc spondylectomy with marginal resection of the DF was performed from the posterior approach. Stabilization was achieved with a multilevel pedicle screw instrumentation and for reconstruction an autologous fibula was used.

*Result:* Thirty-one months postoperatively the girl has no evidence of recurrence and is free from pain.

*Conclusions:* A wide resection of tumors located in the spine is actually impossible to achieve because of the anatomic relationships to the spinal cord, the major vessels and the lung. As in the current case a marginal resection is the maximal one to be reached. Three of seven cases (43%) of DF in the spine treated by intralesional resection showed a local recurrence. These data clarify the importance of a at least marginal resection of DF, if anatomically and technically possible. A local recurrence of DF in the spine can be impossible to treat surgically in a curative intention without a significant loss of function.

**Frequent Modulation of Microvessel Density in Bone Metastases of Various Cancer Types**

Tamás Lőrincz[1], J. Tóth[2], M. Szendrői[1], J. Tímár[3]

*([1]Department of Orthopaedics, Semmelweis University, [2]Department of Human and Experimental Tumor Pathology, National Institute of Oncology, [3]Department of Tumor Progression, National Institute of Oncology, Karolina út 27, Budapest 1113, Hungary)*

Experimental investigations indicate angiogenesis as a major regulator of bone metastasis development. However, there are no studies investigating angiogenesis in bone metastases of human cancers. We have evaluated microvessel density in bone metastases of various cancer types and compared them to their primary tumors of 39 patients. Immunhistochemical staining for vascular endothelial cells was performed using anti-human CD34 antibody. Microvessel density was determined by using the hot spot method. Two patterns of modulation of the angiogenic phenotype in the bone emerged in this study which seems to be cancer type specific: (a) decreased angiogenic potential characterizing renal cell cancer and breast cancer of high vascularity in the primary cancers; and (b) increased angiogenic potential charcterizing lung adenocarcinomas and breast cancers of low vascularity in the primary cancers. Our data demonstrate great variability of vascularisation in primary cancers of various histological origins and in their corresponding bone metastases. It seems that the new micronvironment in the bone tissue modulates the angiogenic phenotype in a significant proportion of cancers. Altered vascular density of cancer metastases in the bone may affect the therapeutic response and the course of the disease.

**Percutaneous Radiofrequency Ablation of Osteoid Osteoma. Clinical/CT MR Imaging Follow-Up**

Federico Portabella, J. Serra, J.A. Narvaez, R. Mast, M. Rovira
*(C/ FEIXA LLARGA S/N, Hospitalet de Llobregat, Barcelona 08907, Spain)*

*Introduction:* Osteoid osteoma is a relatively common benign bone tumour always small in size (less than 2 cm) that is usually found in children and young adults; a characteristic clinical feature is the presence of local pain increased at night with good response to non-steroidal analgesics. The basic radiological element is a distinctive small rounded area of osteolysis (the nidus). Our aim is to review and analize the clinical follow-up, CT/MR imaging characteristics of osteoid osteoma treated with percutaneous radiofrequency ablation (PRFA) in last two years.
*Material and methods:* Between August 2000 and September 2002, 11 patients (six men and five women) with a mean age of 28 have been treated by PRFA in our hospital. Five lesions were in the femur, two in the humerus, one in the homoplate, one in the iliac bone, one the distal radius and one in the acetabulum. Diagnosis was based on clinical symptomatology and imaging techniques (X-rays, scintigraphy and CT). For clinical follow-up patients are interviewed using the modified test of Barei, composed by a preoperative and postoperative components focusing on quantification of pain, limitation of function and activities and the relative importance of those items and the fact to have to take analgesic therapy. For imaging follow-up a CT and MRI is performed 6–12 and 18 months after.
*Results and conclusions:* Before treatment patients were with symtoms during 14 months (range 6–24) and high pain at night (8/10) decreased to 1/10 and day pain almost disappeared (from 6/10 to 0,5/10). Ten patients could continue practising sports normally and re-start their work as they did before; in only one case a reintervention was done and another one because of the location of the lesion (humeral head next to the sinovial) had to change his job. On CT/MR imaging the nidus size decreased in the seven of eight cases that the control have been done as well as decreasing of bone marrow edema and soft tissue edema. The results of the study demonstrate an important and quick pain relief with this tecnique. Most patients reported complete or near complete relief of daytime and nighttime pain, having a fast return to their premorbid functional state. Percutaneous radiofrequency ablation is a simple minimally invasive, safe and effective procedure and it could be the treatment of choice in most cases.

**Angiogenesis Inhibition by Thiol-Modifying Agents – Implications for Therapy**

Katharina Leihtner, Yuri Koshelnick, Andreas Leithner, Bernd R. Binder
*(Schwarzspanierstraße 17, Vienna A-1090, Austria)*

*Background and objectives:* Angiogenesis inhibitors are increasingly used in tumor therapy. The redox-active dithiocarbamates have been shown to protect against side-effects of chemotherapeutic drugs. Increasingly their ability to inhibit angiogenesis is recognized. The mechanism, however, is not completely clear. The aim of this study, therefore, was to analyze the mechanism of angiogenesis inhibition by pyrrolidine dithiocarbamate (PDTC) in an in vitro model.
*Methods:* Capillary-like tube formation was induced in human umbilical vein endothelial cells by plating the cells on Matrigel. PDTC, synergists, or antagonists were added simultaneously with plating and tube formation was quantified. Induction of apoptosis was analyzed by in situ nick translation.
*Results:* PDTC inhibited tube formation in a concentration-dependent manner. The effect was reversed by the thiol reducing agents dithiothreitol and *N*-acetylcysteine, and mimicked by the thiol-modifying agents diamide and *N*-ethylmaleimide. Thiol-modifying agents and PDTC did not induce apoptosis or necrotic cell death, which points to the direction of interference with other intracellular events, e.g., signal transduction, as being causative.
*Conclusions:* Together with data from the literature, the results indicate that dithiocarbamates may act as angiogenesis inhibitors, thus supporting the therapeutic effect of anti-cancer drugs. As a mechanism we propose induction of thiol-modification and thereby interference with the angiogenic response of endothelial cells.

**Anatomical Influences on Functional Outcome in Lower Extremity Soft Tissue Sarcoma**

Craig Gerrand, J.S. Wunder, R.A. Kandel, B. O'Sullivan, C.N. Catton, R.S. Bell, A.M. Griffin, A.M. Davis
*(North of England Bone and Soft Tissue Tumour Service, Freeman Hospital, Newcastle Upon Tyne NE7 7DN, UK, Musculoskeletal Oncology Unit, Mount Sinai Hospital and Princess Margaret Hospital, Toronto, Canada)*

The purpose of this study was to explore the relationship between the anatomical location of lower extremity soft tissue sarcoma and functional outcome.
*Methods:* Function was evaluated with the Musculoskeletal Tumour Society (MSTS 93) rating and Toronto Extremity Salvage Score (TESS). A total of 207 patients of median age 54 years (15–89) were eligible. The median maximum tumour diameter was 8.0 cm (0.3–36.0). A total of 58 tumours were superficial and 149 deep. Nine locations based on anatomical compartments were defined. Six tumours were in the groin/femoral triangle, eight in the buttock, 52 in the anterior thigh, 22 in the medial thigh, 20 in the posterior thigh, 10 in the popliteal fossa, 13 in the posterior calf, 11 in the anterolateral leg and seven in the foot or ankle.
*Results:* Treatment of superficial tumours did not lead to significant changes in MSTS (mean 90.6 vs. 93.0%, $P = 0.566$) or TESS (mean 86.4 vs. 90.9%, $P = 0.059$). However, treatment of deep tumours led to significant reductions in MSTS and TESS (mean 86.9 vs. 83.0%, $P = 0.001$; mean 83.0 vs. 79.4%, $P = 0.015$). There were no significant differences in MSTS and TESS when overall scores were compared by anatomical location. Exploratory analysis of MSTS subscales showed groin tumours were more painful than others, and posterior calf tumours had the lowest scores for gait. Analysis of TESS subscales suggested groin and buttock tumours were associated with difficulty sitting, and groin tumours were associated with difficulty dressing. Further exploratory analysis suggested 'conservative' surgical excision of low-grade liposarcomas in all locations was associated with a significant decrease in functional scores.
*Conclusion:* Anatomical factors known to influence functional outcomes include tumour size, bone and nerve resection. There is significant variation in MSTS and TESS subscale scores when anatomical locations are compared. The 'conservative' surgery used in the treatment of low-grade fatty tumours in all locations has a significant impact on functional scores.

**Activity of Ecteinascidin 743 (ET-743) in Patients with Metastatic Sarcomas: a SENDO (Southern Europe New Drug Organisation) and ISG (Italian Sarcoma Group) Phase II Study**

Stefano Ferrari, G. Bacci, P.G. Casali, M. Casanova, M. Aglietta, C. Sessa, P. Picci, N. Ielmini, S. Marsoni
*(Sezione di Chemioterapia, Istitituti Ortopedici Rizzoli, Bologna 40136, Italy, Dipartimento di Oncologia Muscoloscheletrica Istituti*

*Ortopedici Rizzoli, Bologna, Istituto Nazionale Tumori, Milano, Istituto Ricovero Cura Cancro, Candiolo, Torino, Ospedale S. Giovanni, Bellinzona, Switzerland, SENDO, Milano, Italy)*

Data showing activity of ET-743 administered as a 24-h continuous infusion in advanced pretreated sarcomas were reported in patients treated in phase II-studies and in compassionate use programs. A phase II study was planned to evaluate the activity of ET-743 as a 3-h infusion in advanced soft tissue sarcomas (STS), osteosarcoma (OS), and Ewing's family tumors (EFT). From October 2000 to January 2003, 65 patients (25 STS, 19 OS, 21 EFT) entered the study. Median age was 42 years (18–71) for STS, and 26 years (13–56) for other sarcomas. ECOG PS was 0–1. All patients were heavily pretreated with chemotherapy ($N$ regimens: median 2, range 1–4; $N = 17$ with HD-MTX; $N = 13$ with HD-chemotherapy w/PBCS support). The drug was given as a 3-h IV infusion every 3 weeks. The initial dose was 1300 or 1500 $\mu g/m^2$ depending on extent of previous treatment, and adjusted according to toxicity. Two patients with STS (one Leiomyosarcoma and one liposarcoma) had a partial response (PR), for a PR rate of 8.7% (95% CI = 1–28%). Two further patients with synovial sarcoma had a minor response lasting respectively five and seven cycles for an OR rate of 16% (95% CI = 5–36%). Two PR were observed in EFT for a PR rate of 10% (95% CI = 1.23–31.7%). According to the resist criteria no responses were seen in OS, but in one patient a metastatic pleural effusion disappeared after two cycles of ET-743. One toxicity death (associated with myelosuppression and acute respiratory failure, respectively) were recorded in STS. The main toxicity seen was liver related; ASAT and/ALAT (G3: 56%, G4: 18%), alkaline phosphatase (G3 2%, G4 1%); bilirubin (G2 9%). Other drug-related toxicities (G3/4) included: asthenia (G3 2%), thrombocytopenia (G3 7%, G4 1%), neutropenia (G3 24%, G4 22%). Nausea and vomiting (G3 2%; $n = 2$) is mild and easy controllable by the use of antiemetics. This study confirms that ET-743 has activity in heavily pre-treated STS patients, apparently in the same range as the 24-h infusion, including possibly EFT cases. The drug is usually well tolerated; however, occasionally associated to a risk of serious toxicity. The drug is worth further assessment in sarcomas possibly focusing on the activity/toxicity associated with new schedules.

### Resections–Reconstructions with Megaprostheses in Bone Tumors. Long-Term Results of 147 Cases

Sergio Mapelli, P.A. Daolio[1], F. Lazzaro[1], G. Perrucchini[1], P. Zacconi[1], R. Zorzi[1], G. Oldani[1], P. Gozzini[1], F. Ugazio[1], E. Usellini[1], N. Parafioriti[2]
*([1]Orthopaedic Oncology Surgery, [2]Pathology, Gaetano Pini Orthopaedic Institute, Via G. Pini 9, Milano 20122, Italy)*

From the whole series of 340 megaprostheses implanted in the Center of Orthopaedic Oncology Surgery after oncologicalical resections, the authors selected 147 cases with long-term follow-up ranging from 25 to 10 years. The oncological results concern primary and metastatic lesions; the orthopaedic ones are reported looking at the differentent anatomical sites. Age, sex and sites of 109 primary and 38 metastatic tumors are examined. The oncological data consider the histology, the stage, the extent of the resection and the surgical margins, the necrosis when available and the follow-up. The orthopaedic data analyze the type of reconstruction and the models of the prosthesis in the different sites, the functional results, the early and late mechanical and surgical complications, the final outcome. The general results show 45% of patients died of the disease (this rate decreases up to 27% if related only to the primary tumors); 50% of the patients are alive and NED while 5% are lost to a follow-up. The more frequent complications were mechanical and a surgical revision was required in 30% of the cases (half of this revisions were performed for the change of the polietylene ne busches in the old model of the KMFTR system, now modified). No secondary amputations were done in this group. The main complication are the infections, most of them related to a revision surgery for oncological or orthopaedic reasons; they rated 13% and caused three amputations. Functional results were evaluated in the different sites with Enneking method. Better results were obtained in the hip recontruction; in the knee, femoral prostheses gave higher functional scores than tibial ones; shoulder replacements were functionally lower, as expected. In summary, the long-term results of the prosthetic replacement confirm the effectiveness of the mechanical reconstructions. The greatest care has to be recommended in the surgical technique and in the choice of experienced prosthetic models in order to minimize the possible complications. The younger patients require a longer follow-up and only in the long run it will be possible to know the final outcome of their prostheses.

### Long-Term Results of Treatment of Giant Cell Tumors

Sander Dijkstra, A.P. Suijdendorp, A.H.M. Taminiau
*(Leiden University Medical Centre, Albinusdreef 2, Postbus 9600 2300 RC, Leiden, The Netherlands)*

*Introduction:* Treatment of giant cell tumors (GCT) has changed over the years, and still different techniques are used, such as resection, curettage, with or without adjuvant therapy. The long-term results of these techniques are evaluated.
*Material and method:* Clinical and functional data of 68 patients with giant cell tumors treated at the Leiden University Medical Centre, whose treatment started between 1969 and 1993, were included in this study. There were 35 males and 33 females. Age at start of therapy was average 30 years (13–59). Almost all giant cell tumors (64) were Enneking stage 3. Primary locations of giant cell tumor: femur, 22; tibia, 12; radius, 11; humerus, 7; fibula, 3; pelvis, 3; foot, 2; hand, 5; spine, 2; ulna, 1. Treatment consisted in curettage (47) and resection (21). When curettage was performed, adjuvant therapy could be cryotherapy, application of phenol or cementation or a combinations of these. Mean follow-up was 15 years.
*Results:* At >10 years of follow-up of these 68 patients, 63 are disease free, four died related to comorbidity, one patient has recently another (fourth) recurrence. The recurrence rate over all was 24 (35%). Eleven patients had a second recurrence, five patients three times, two patients four times and one recurred six times. The first recurrence was found at an average of 1.5 years (0.2–6.5), second and third recurrences after 2 and 1.5 years, respectively. Two patients sustained metastases and were treated successfully with chemotherapy. Recurrence rate was non-depended of tumor grade. In 6/40 total recurrences in 21 patients the tumor grade increased, three at first recurrence, three thereafter. The recurrence rate was higher in females (68%). Only in female patients more than two recurrences were found. Recurrence occurred in all locations without preference. A total of 48 of 68 cases were treated by curettage. In 10 patients no adjuvant therapy and in 38 some kind of adjuvant therapy was given. Adjuvant consisted in cementation alone 21 (recurrence 8), cementation with phenol 9 (rec. 2) and cryosurgery and cementation one (rec. 0). Nine patients with curettage alone had cancellous bone grafting, one had no grafting at all. Nine of 10 had recurrence. Subchondral cancellous bone graft in combination with cementation was given in 16 in the adjuvant group. All recurrences were treated by curettage, adjuvant (phenol) and cementation. In 20/68 patients the primary tumor was resected. For reconstruction none (6), arthrodesis (2) endoprostheses (3), bonegrafts (6) and bonegraft-composites (3) were used. One patient had cryotherapy as adjuvant and three patients had a cement reconstruction. Recurrence rate in the resection group was 5/20 (25%).

Since most of recurrences were in soft tissue, resection was used as a therapy for recurrence.

*Complications:* Serious complications were: exarticulation of the knee due to vascular, neurological, soft tissue and bone damage after several large recurrences in polyostotic giant cell tumor (1), prosthesis due to allograft failure (2), graft resorption needing new graft (3), Thiersch graft due to skin necrosis (1), arthrodesis after graft failure (1), wound infection needing surgery (3), paresis after spine surgery (1), pathological fracture treated by osteosynthesis (3) ischiadicus/peroneus palsy (each 1). The functional MSTS score showed: 48 patients between 27 and 30, 16 between 20 and 27 and four scored less than 20.

*Summary:* A total of 21 of 68 patients with over 10 years of follow-up recurred (35%). The highest rate of local recurrences (89%) was seen in treatment with curettage alone and no adjuvant. Recurrence after curettage and adjuvant is significant; however, the groups are small: cement alone 38%, cement and phenol 22%, resection 28%. Females have a higher recurrence rate than males and have more recurrences. Final results were satisfactory in 94% of cases.

**Is it Worthwhile to Preserve the Patient's Knee? More than 10 Years Follw-Up of 74 Malignant Bone Tumours around the Knee**

Rodrigo Dölz, San-Julian, Mikel

*(Department of Orthopaedic Surgery. University of Navarra, Pamplona 31080, Spain)*

*Aim:* To compare several reconstruction methods regarding duration, complications and function in malignant tumours around the knee.

*Methods:* 74 patients with malignant bone tumours either in distal femur or proximal tibia were followed during at least 10 years (mean 14, range 10–23 years). Mean age at diagnosis was 20 years (range 6–61). Intercalary non-vascularized autograft (7) (IAu) or allografts (22) (IAl) were used when the patient's knee could be preserved. Osteoarticular allografts (4) (OAl), arthrodesis (Ar) (two auto and one allograft) or total knee arthroplasty with plastic (14) (pTKA) or allograft (24) (aTKA) spacer were used when the epiphysis was involved by the tumour. The complications, the final outcome and functional results were studied in every reconstruction method.

*Results:* After 10 or more years of follow-up, 17/21 radiated and in 52/53 non-radiated patients still preserve their affected limb. Re-operation in intercalary reconstructions was necessary in 60% of the cases. However, most of them were bone autograft supplementation in order to achieve auto or allograft consolidation to the host bone. A total of 19 IAl remain with excellent function at the last follow-up (12 years). Six of seven IAu remain intact and with excellent function after a mean follow-up of 17 years. Arthrodesis required beteween 1 and 3 autograft supplementation. No OAl achieved long-term success. Half of pTKA were converted to aTKA due to aseptic loosening. aTKA did not have this problem. Eight aTKA required exchange, two were converted to arthodesis and two were amputated.

*Conclusion:* Preservation of the patient's knee avoids prosthesis complications. A total of 26 of 29 intercalary reconstructions remains as such and 70% had excellent or good functional score, while 11/38 prosthesis required exchange to another reconstruction method, and six more prostheses were exchanged due to infection. At the last follow-up, 62% of articular reconstructions had excellent or good functional results. OAl in the knee did not achieve good results.

**Follow-up of 10 and more Years after Skeletal Reconstructions with Kotz Special Endoprosthesis in 31 Patients**

Miroslav Smerdelj[1], D. Orlic[1], B. Baebler[2], R. Kolundzic[1], M. Ponikvar[2]

*([1]Department of Orthopaedic Surgery, School of Medicine, University of Zagreb, Croatia, [2]Department of Orthopaedic Surgery, University Clinical Center, Ljubljana, Slovenia, Salata 6, Zagreb 10000, Croatia)*

From 1987 to 2001, special modular uncemented endoprostheses were implanted in 82 patients. After more than 10 years, 31 patients were controlled, and the results of their skeletal reconstruction after resection were presented. In four patients indication for skeletal resection was giant cell bone tumor. Malignant bone tumor were registered in 22 patients, aneurismatic bone cyst in one patient, and different conditions (like instability of endoprosthesis, two massive hip destruction after war trauma and Paget disease) were indications in four patients. The results of treatment in 31 patients were analyzed after follow-up of 10 and more years; six patients are dead of disease and one patient because of other disease related to the age. Because of the situations during the war five of our patients disappeared. Eighteen of our patients are with no evidence of diseases, but 10 of them were treated because of some complications. Infection developed in five of our patients, extraction of endoprothesis was done in three of them, but only the girl with osteosarcoma and hepatic lesion went to rearthroplasty. Several reoperations were done because of different technical problems in patient with GCBT. Local recurrence was registered in five patients. We believe that endoprostheses remain the treatment of choice for reconstructions after resections when the tumor is localized near the joint. The high price of that uncemented endoprosthesis is important and significant factor in our country.

**Abdominal Wall Desmoid – Case Report**

Balázs Villangó[1], L. Juhász[1], T. Labancz[1], M. Gondos[1], P. Széphalmi[1], Sz. Takács[2], L. Vass[3], K. Kammerer[4], P. Rahóty[1]

*(Central Hospital of MI, Budapest, Hungary, [1]Surgical Dept. [2]Pathology Dept., Flór Ferenc County Hospital, Kistarcsa, Hungary, [3]Pathology Dept., [4]Oncology Dept., Városligeti fasor 9-11, Budapest H-1071, Hungary)*

*Introduction:* Desmoid tumors, also known as aggressive fibromatosis, comprise 0.03% of all solid tumors. Desmoids tend to occur in young women. The etiology is unknown, although associated local or surgical trauma, hormonal effects, especially estrogenic hormones, pregnancy, and heredity. Increased frequency has been seen in association with familial adenomatous polyposis. Desmoids are characterized by infiltrative growth, complications by their constricting effect on the neighboring organs, high incidence of recurrence (ranging between 25 and 40%), but their metastases is unknown.

*Case report:* We report a 31-year-old female who presented in our institution with a painful mass of the left side of her abdominal wall. She complained of previous accidental trauma 6 months before. Clinical examination demonstrated a firm, tender mass in the middle part of left rectus muscle, made prominent on abdominal contraction. Ultrasonography, MRI showed a $15 \times 15 \times 30$ mm solitary, hyperdense mass, laboratory findings were normal. Fine needle aspiration biopsy suggested a desmoid tumor. During the surgical procedure the whole left rectus muscle was removed with the anterior part of the rectus fascia, with the ligation of the upper and lower epigastric vessels. We closed the wound primarily. The patient was discharged to home on sixth postoperative day. Pathological examination revealed a desmoid tumor with a 4 and 6 cm wide tumor-free margins. Now, we are making a close follow-up including clinical, hormonal status examinations, and imaging controls, for 3 year examinations at 3-month intervals.

*Comments:* Surgical margins should be wide. Even with tumor negative margins the local recurrence rate is high. Radiotherapy can be effective with surgical excision for extra-abdominal desmoids. As an adjunct to primary surgery 55–60 Gy can be given over 6–8 weeks. Pregnancy and oral contraceptives seem to stimulate the growth of desmoids, and these tumors are most common in fertile women. The hormonal influence on development and growth gives alternative pharmacological approaches including anti-estrogenic therapy. Non-steroidal anti-inflammatory agents are also used because of their immunological effects. Chemotherapy does not seem to be effective, probably due to low mitotic index of these tumors. At present, en bloc, complete surgical excision with wide margins and postoperative high dose radiotherapy avoiding recurrence seems to be the most successful method to cure desmoid tumors.

**Use of Vein Graft as a Tendon Sheath Substitute following Tendon Repair: an Innovative Technique in Tendon Surgery**

Reza S. Moosavi[1], A.R. Kalantar Motamedi[2]

*([1]Associate Professor of Vascular and Trauma, Department of General and Vascular Surgery and Traumatology, Shohada Medical Center, Shahid Beheshti University of Medical Sciences, Tehran, Iran, [2]Attending Vascular Surgeon and Assistant Professor of Surgery, Hazrat Rasoul Medical Complex, Iran University of Medical Sciences, Tehran, Iran)*

*Objectives:* This is a new technique for managing tendon repair which can improve the results of existing methods.

*Methods:* A total of 105 patients with new or old tendon injuries or complications of previous repair underwent tendon repair by modified Kessler method and a portion of the saphenous veins was used to cover the repaired tendon: 90 patients had flexor tendon injuries which involved zones 1–5, and 15 patients had extansor tendon injuries (zones 5–7). A modified Kessler technique with 3-0 prolene was used for the core suture. Afterwards, a running 6-0 nylon or prolene epitendinous suture was used to even the repair site. After the tendon repair, a segment of vein which the tendon has been passed through prior to the repair was used as a tendon sheath substitue. A 6-0 prolene was used for anastomosis of the proximal and distal ends of the sheath defect to an interposed segment of autogenous or frozen vein.

*Results:* Our preliminary results appear encouraging when compared with outcomes achieved by conventional tendon repair techniques.

*Conclusions:* Because this technique reduce the adhesion formation, and also improve tendon nurishment, and also decrease the need of intensive physiotherapy, it can be a standard choice in the future.

**Bone Metastases of Conventional (Clear Cell) Renal Carcinoma**

Zdenek Matejovsky Jr., Matejovsky Zdenek

*(Orthopedic Clinic Bulovka, 1st Medical School, Charles University Prague, Prague CZ 180 81, Czech Republic)*

Purpose of the study is to evaluate our experience with 218 patients with skeletal metastases of conventional renal carcinoma and to look for long-term survival after nephrectomy and treatment of skeletal metastases. Conventional (clear cell) renal carcinoma is the most frequent malignant renal tumor and represents about 75% of all malignant renal tumors. The most frequent site of metastases for this tumor are the lungs and the skeleton. In the years 1966–1998 (33 years) we have treated 218 patients (145 men and 73 women) for skeletal metastases of this tumor. The average age of the patients was 56.9 years. There were four patients under the age of 40 (31, 36, 38, 39) and two patients over 80 (81, 86). A total of 161 patients had clinically solitary metastases: 45 femur, 40 humerus, 19 pelvic, 15 scapula, 14 tibia, 17 spine, 11 other localisations. A total of 57 patients had multiple bone metastases or involvement of other organs (lungs). Most patients had already had nephrectomy, but in some cases the bone metastases was the first sign of the tumor. The diagnosis was established from X-ray pictures in two planes. Scintigraphy was perfomed to exclude further occult skeletal involvement. In a majority of patients proposed for surgery arteriography was performed. First direct injection of contrast by Seldinger catheter, later as DSA. In the later years CT and MRI improved the diagnosis especially as to exact position and extent of tumor. From all malignant tumors and metastases conventional renal carcinoma forms the most vascularised bone metastases, which may lead to complications during surgery. Due to this we performed, especially in spinal and pelvic localisation, peroperative embolisation. In the treatment we have almost abandoned intramedullary fixation by Kuenthscher and other nails and we prefer plate ostheosynthesis and cement filling of the cavity. After this procedure external adjuvant radiotherapy usually follows. The ends of long bones – proximal and distal femur, upper tibia and proximal humerus – were replaced by endoprostheses. In examining the survival time, we found that most of the patients died within 5 years. We have 13 patients who survived disease-free longer than 5 years. From our experience we conclude that, exceptionally, skeletal metastases from conventional renal carcinoma may be solitary and nephrectomy together with a radical treatment of the metastases may result in long-term survival.

**Functional Results of Different Shoulder Resections in The Treatment of Musculosceletal Tumors**

János Kiss

*(Gergely Sztrinkai, Imre Antal, Jenő Kiss, Miklós Szendrői, Karolina Str. 27, Budapest 1113, Hungary)*

*Aim of the study:* Radical resections of musculosceletal malignant tumors may extend the life of patients but they can lead to significant functional or cosmetic loss. Our aim was investigate the results of radical tumor resections of the shoulder region.

*Material and methods:* A total of 91 shoulder resections on 90 patients were performed at our institution between 1981 and 2001. There were 53 males and 37 females with an average age of 42 years (11–76). The average age of the patients with primary tumor was 34 years, while it was 61 years in case of metastasis.

*Results:* A total of 37 patients were clinically reviewed by using the recommendations of the Musculosceletal Tumour Society like pain, function, position of hand, lifting ability, manual dexterity and satisfaction. All were recorded from 0 to 5 marking the best scenario as 5. An additional nine patients were reviewed by using a questionnaire and telephone interview. The overall follow-up was 4.7 years (1–20) but it was 5.6 in case of primary bone tumour and 2.9 in case of metastasis. We had records on the death of 26 patients. A total of 18 patients were lost to follow-up.

*Discussion/conclusion:* The overall satisfaction is generally good following different types of shoulder resections because of the pain relief, the preserved hand function, and the improvement of psychological status. The patients can compensate extremely well by using the preserved articulations and the contra lateral upper limb therefore the patient satisfaction does not rely on the shoulder function alone.

**Neoadjuvant Chemotherapy (CT) +/· Immunotherapy (IT) in Newly Diagnosed Metastatic Osteosarcoma of Childhood**

Graziella Silva Cefalo[1], R. Luksch[1], M. Casanova[1], A. Ferrari[1], M. Massimino[1], D. Polastri[1], M. Terenziani[1], F. Spreafico[1], S. Cereda[1], L. Gandola[1], P. Daolio[2], S. Mapelli[2], A. Parafioriti[2], F. Ravagnani[1], P. Collini[1], G. Perrucchini[2], F. Fossati-Bellani[1]
*([1]Istituto Nazionale Tumori, Via Venezian 1, and [2]Istituto Ortopedico G. Pini, Milano 20133, Italy)*

Despite the last two decades have shown a dramatic improvement in the long-term disease-free survival of localized osteosarcoma, a poor prognosis is still observed in patients presenting with synchronous metastatic disease. Modification of the chemotherapeutic regimens as far as timing and selection of drugs were only of little benefit in improving the prognosis. Between 1987 and 2001, 32 consecutive patients (M 20, F 12; age 5–18 years) with metastatic high grade osteosarcoma at onset were treated at our institution with multimodal treatments, including neoadjuvant CT, surgery of the primary tumor, and surgery of metastatic sites, when feasible. Radiotherapy was recommended when surgery was not feasible. Up to 1990, patients received pre-operative CT with MTX ($8\,g/m^2$) + VCR and CDDP ($40\,mg/m^2$/day × 3 days) + IFO ($1.5\,g/m^2$/day × 3 days). Postoperatively, patients received the same drugs used preoperatively. From 1991, to improve the outcome, the treatment program was modified by adding IT. A first course of rIL2 (9 milion IU/$m^2$ × 4 days) followed by leukapheresis, in vitro LAK-cell activation with rIL2 and storage, was administered before the first cycle of CT. Three additional rIL2 courses with LAK-cell reinfusion were repeated before surgery, and at the end of the treatment program. No chemo- or IT-related deaths were recorded, and toxicity was always manageable. Twenty-three of 32 had metastatic sites only in the lung, nine had metastatic sites in other bones with or without lung involvement. Twenty-three of 32 patients received surgical treatment for the primary tumor (limb-sparing surgery in 14, amputation in nine), five patients received radiotherapy and in the remaining four patients no local treatment was performed because of tumor progression. After a median follow-up of 5 years, the 3-year EFS and survival (S) were 35.5 and 48.6%, respectively. Considering the two consecutive study protocols, the 3-year EFS and S of the program with IT (patients = 20) were 46.3 and 57.6%, respectively, as compared to 16.7 and 33% of the program without IT (patients = 12). Clinical results show that the group of patients treated with IT fared better than the group without IT ($P = 0.03$). In conclusion, the results indicate an advantage for the use of immunotherapy suggesting a role of IT in the management of childhood osteosarcoma. In addition, an aggressive surgical approach to pulmonary metastases as well as to primary tumor has also proved of measurable benefit. These results in metastatic osteosarcoma at diagnosis are encouraging and should stimulate oncologists, surgeons and radiotherapists to pursue the use of aggressive management of these patients.

**Long-Term Results with Mega Prostheses in Adults with Malignant Tumors of the Lower Extremity + Pelvis**

R. Kotz

Between January 1983 and December 1992, 94 megaprostheses in the lower extremity were operated on in the presence of malignant primary tumors. Histologically the entities were as follows: 43 osteosarcomas; 17 chondrosarcomas; 13 Ewing's sarcomas (PNET); 10 malignant fibrous histiocytomas; and five fibrosarcomas. All other diagnoses were encountered only once each. The locations were as follows: the distal femur in 37 cases, the proximal tibia in 23 cases, the proximal femur in 16 cases, the ilium in 13 cases, other locations in five cases. Wide resection margins were achieved in the majority of cases (69) of the Caver extremty tumors. Radical surgery was performed in a few cases (3); one resection was marginal 1 and one resection inter-lesional, while two amputations had to be performed because of local relapse. The remaining operations had been performed because of complications: exchange operations were carried out in 31 cases, axis revision in 40 cases and soft tissue revision in 32 cases. The following operations were performed 4 times each: osteosynthesis for fractures, repositioning of luxations, plastic reconstruction. The following were performed two times each: augmentation of the fascia, redressement or synovectomy. Other procedures were performed in six cases. Thirty-four of 94 patients died. Only 29.9% of these were patients with tumors of the extremities, whereas 64.7% had undergone pelvic operations. The latter constitute a much greater difficulty in the treatment of pelvic tumors to achieve wide or even radical margins compared to other tumors in the lower extremity.

**High Dose Chemotherapy with Peripheral Blood Stem Cells (PBSC) Support in Relapsed Ewing's Sarcoma/PNET (ES), in Young Adults**

A.Tienghi[1], P. Giovanis[1], M. Del Duca[1], S. Palazzi[1], L. Zornetta[2], G. Papiani[1], D. Donati[3] , M. Manfrini[3], S. Ferrari[3], M. Mercuri[3]
*([1]Dept. Of Oncology & Radiotherapy, City Hospital, Ravenna, [2]Blood Bank & Biochemistry Lab, City Hospital, Ravenna, [3]Muskulo-skeletal Oncology Dept., Rizzoli Institute, Bologna, Italy)*

From October 1998 to February 2002, we treated 20 young adults with relapsed Ewing's sarcoma. There were 14 males, six females; median age 28 years (range 16–43). Sites of primary tumour were pelvis in seven patients (patients), extremities in nine, scapula in two, vertebra in one and kidney in one. Six patients were in second or third relapse, two had lung metastases at diagnosis, one had progressive disease after CHOP for initial wrong diagnosis of bone and soft tissue lymphoma, and one was in PD after chemo- and radiotherapy. Ten patients were treated at first relapse: median free period from the end of primary treatment was 13 months (range 7–72). Sites of relapse were lung in 11 cases, local in four, local plus lung in two, bone in two, and lung plus liver in one. Regarding primary treatment for Ewing's sarcoma, they received eight to 13 cycles of chemotherapy with EDX, Act-D, ADM, VRC, IFO, VP 16, in addition to surgery and/or radiotherapy. All patients received CTX $4\,g/m^2$ and VP 16 600 $mg/m^2$ + G-CSF (10 patients after re-induction chemotherapy, plus surgery in three of them), to harvest PBSC, achieving a median of $4.5 \times 10^6$/kg CD34+ (range 2–20), followed by high dose Busulfan 4 mg/kg × 4 days orally and Alkeran 140 $mg/m^2$, with PBSC support and G-CSF, until granulocyte recovery. No toxic death was observed. Two patients experienced reversible VOD. All but two patients achieved remission after chemotherapy +/· surgery or total lung RT. So far, seven patients are NED at 24 months (range 12–35), six are alive with disease at 21 months (range 14–38) since relapse, while seven died of disease. High dose Busulfan and Alkeran + PBSC and G-CSF seems to be very effective in this heterogeneous group of patients with relapsed Ewing's sarcoma. A new protocol by Italian Sarcoma Group, has just begun for patients at first relapse.

**Surgical Treatment of Monosegmental Lumbar Metastasis of Breast Malignancies**

Peter Paul Varga
*(Nagy Jenő u. 8., Budapest 1126, Hungary)*

By the development of the oncological knowledge, the number of breast cancer patients with skeletal metastasis increases. However these patients and these metastases require principally complex oncotherapy, but even in case of successful radiotherapy or chemotherapy, the vertebral manifestation of the tumour could lead to pathological fracture and neurological symptoms. The consequences of pathological fracture do not only significantly influence the quality of life of the patient, but also the life expectancy. The data of the breast cancer patients with monosegmental lumbar metastasis operated with total segment resection between 1992 and 2003 will be discussed in details. The patients were operated at the National Center for Spinal Disorders and previously at Semmelweis University, Department of Orthopaedics.
*Method:* The patients were referred to us by the Hungarian oncological network, mostly by the National Institute of Oncology. They were examined and prepared before the referral, and the indication for surgery was proposed for the patients on mutual base. The prevalent symptoms were: local pain with or without lumbosciatica and incontinence. Following preoperative preparation standard vertebrectomy, replacement and stabilization were done according to Beriani or Tomita. The vertebral replacement had components: for ventral mechanical support any metal construct (Fidler Jack or our own implant) and bone cement as filler. The affected and adjacent vertebral segments were stabilized by modular transpedicular implants. Following the operation the patients were rehabilitated according to our institutional protocol, and then sent back to the oncological network. For the following 2 years we continued regular follow-up examinations including X-rays.
*Results:* Of the 231 vertebrectomies performed, 91 were breast cancer metastases. The patients ranged between 36 and 87 years (mean 55.4 years). Intraoperatively major complication (injury of large vessels and intestines or bleeding) did not occur, accidental dural sac injury occurred in four cases. In six cases unilateral nerve root (at l–2 and 3 levels) were dissected for posterior mobilisation of the vertebral body. Postoperatively, we had two deep wound inflammation, which were treated by evacuation and perfusion of the wound (both patients had local irradiation previously). We had no reoperation in this group because of local tumour recurrence. Six cases developed vertebral metastases in the later postoperative phases. We will analyze in detail patients neurological symptoms and every day activity as well as their survival data.
*Conclusion:* The total segment resection is a safe and successful method for the surgical treatment of soliter lumbar metastasis of breast cancer not only because of the good clinical results obtained in the treatment of pathological fractures, but also because of long-term affect on the eventual course of the basic oncological disease. In addition to improving the patient's quality of life, removal of the tumour provides a positive psychological experience that may lengthen a patient's life span.

### The Management and Prognosis of Patients with Pathological Fracture through Localised Ewing's Sarcoma

H. Ahrens, A. Abudu, S.R. Carter, R.M. Tillman, R.J. Grimer
*(The Royal Orthopaedic Hospital Oncology Service, Birmingham, England)*

Twenty-seven patients with pathological fractures through primary localised Ewing's sarcoma were retrospectively studied to determine the influence of the fractures on limb preserving surgery, rate of amputation, local tumour control, overall survival and metastasis-free survival. The mean age at diagnosis was 20 years (5–37). There were 14 males and 13 females. Eighteen patients had fractures at the time of diagnosis of the tumour and nine developed fracture during treatment. Median possible follow-up was 118 months (24–278). Tumour location was femur 12, humerus seven, pelvis three, tibia four and radius one. All the patients had chemotherapy following which the fracture united in the majority. Local treatment was surgery alone 15, surgery and radiotherapy five and radiotherapy alone in seven patients. Nineteen of the 20 patients who had surgery had limb-preserving surgery including simple excision in one and excision with endoprosthetic reconstruction in 18. One patient had hindquarter amputation. Surgical margins were wide in 17, marginal two and intralesional in one patient. Local recurrence occurred in two patients (7%) and metastases in 11 (41%). The cumulative overall survival and metastasis-free survival at 5 years was 60 and 56%, respectively. The survival of patients who presented with fracture was similar to those who developed fracture during treatment. The two patients who developed local recurrence had been treated with radiotherapy only and none of the patients who had surgery with or without radiotherapy developed local recurrence. We conclude that the majority of pathological fractures through Ewing's sarcoma will unite with chemotherapy and that limb salvage with adequate margins can be achieved in these patients. The rate of local control and survival is similar to those with Ewing's sarcoma without fractures.

### Pyridinoline Cross-Links as Markers for Primary and Secondary Bone Tumors

Juergen Bruns, Y. Acil, K.P. Ullrich, P. Behrens
*(Dept. Orthop. Surgery, University of Hamburg, Hamburg D-20246, Germany)*

Determination of hydroxylysylpyridinolines (HP) and lysylpyridinolines (LP) in urine is a promising method to determine bone resorption. It has been used for several studies in several oncological questions. This method is independent from gender, diet, and kidney function (creatinine clearance >25 ml/min). In the present study we evaluated the value of HP and LP in urine from adult tumor patients suffering from primary malignant bone tumors ($n = 24$), bone metastases ($n = 38$) and soft tissue sarcoma with additional osseous involvement ($n = 13$). The values were compared with those obtained from 698 healthy controls (0.5–65 years old). The analyses were performed by means of a HPLC evaluating the whole content of HP & LP.
*Results:* Results clearly exhibited a significant increase in HP values (57.75 nmol/mmol creatinine) in adult tumor patients (aged 15–65 years) of all three subgroups in comparison to the control group (22.23 nmol/mmol creatinine). Although the LP fraction is more specific for bone than HP the values of LP from all subgroups of the adult tumor patients were less distinctly but significantly increased. Regarding the HP:LP ratio, tumor patients exhibited a distinctly increased average molar HP:LP ratio (12.0:1) in comparison to controls (6.6:1). Correlation analysis for linear correlation between HP and LP resulted in $r = 0.98$ for controls and $r = 0.8$ for tumor patients. In conclusion, determination of HP and LP in urine obtained from adult tumor patients suffering from malignant primary bone tumors, metastases to bone and osseous involvement in cases of soft tissue tumors is a useful marker for bone resorption and can be indicative for an osteolytic bone tumor. Further studies have to demonstrate whether determination of HP, LP and its ratio can be used to follow-up the course of the tumor patients regarding detection of a possible relapse of the tumor.

### Role of Extracellular Matrix in Proliferation of Human Osteosarcoma

Revekka Harisi

*(Üllői út 26., Budapest 1085, Hungary)*

*Introduction:* The proliferation and metastatic potential of human osteosarcoma may also be determined by the signal transduction pathways related to extracellular matrix components. Since the extracellular matrix construction is principally different in tumours from normal tissue, this point of view may earn significance in understanding of growth properties of this malignancy.

*Aims:* In this study involvement of extracellular matrix and its components in osteosarcoma cell proliferation and tumour progression was investigated.
*Materials and methods:* A stabile osteosarcoma cell line (OSCORT) was established from the biopsy fragments of a 17-year-old boy. Osteosarcoma cells were cultured conventionally, or on extracellular matrix gel (ECM-gel), as well as on the matrix produced by them (OSCORT-ECM). Cell proliferation, cell cycle analysis, expression of proliferation-related proteins and collagenase IV activity was compared within these culture conditions.
*Results:* ECM-gel and osteosarcoma matrix increased cell proliferation (cell numbers and thymidine incorporation), which was confirmed by increased cyclin D1 and PCNA protein expression. Among ECM components heparan sulfate proteoglycan (HSPG) and fibronectin was the most responsible for these effects. On the other hand, laminin did not change, and collagen IV definitively decreased cell proliferation. Representation of S-phase of cell cycle has double increased among cells kept on ECM-gel or treated with HSPG or fibronectin. Collagen IV instead, significantly decreased G1- and S-phase and increased G2-phase counts. Expression of cyclin B1 decreased, while, PCNA and Ki-67 increased in cells cultured on ECM-gel. The decrease of cyclin B1 and cdc2 was also observed in presence of all studied ECM components. Cyclin D1 expression was increased by ECM-gel, heparan sulfate proteoglycan and fibronectin, was not changed by laminin, and was decreased by collagen IV. Collagenase IV activity was induced by ECM-gel, which was most enhanced for HSPG.
*Conclusion:* We conclude that ECM has significant role in maintenance of malignant phenotype of human osteosarcoma, and a unique heparan sulfate proteoglycan produced by osteosarcoma cells may partly be responsible for this effect, which can be addressed as potential therapeutic target in the future.

**NU-159548: a Promising New Anticancer Agent for High-grade Osteosarcoma Treatment**

Massimo Serra, Reverter Branchat Gemma, Pasello Michela, Hattinger Claudia Maria, Manara Maria Cristina, Benini Stefania, Astolfi Alessia, Scotlandi Katia, Picci Piero
*(Laboratorio di Ricerca Oncologica, Istituti Ortopedici Rizzoli, Via di Barbiano 1/10, Bologna 40136, Italy)*

PNU-159548 (3′-aziridinyl-4′-methylsulphonyl-daunorubicin) is the lead compound of a new class of antitumour agents that retain their activity also in cells resistant to various alkylating agents (including cisplatin) and in MDR-1 gene-overexpressing, multidrug resistant cells. Since the increased of P-glycoprotein levels, as a consequence of MDR-1 gene overexpression, has been reported to be the most important adverse prognostic factor in high-grade osteosarcoma (OS), the in vitro effectiveness of PNU-159548 was evaluated on a wide panel of human OS cell lines. In particular, the in vitro activity of PNU-159548 was assessed on 10 cell lines established from surgical specimens obtained from patients with high-grade OS, as well as on 20 human OS cell lines resistant to doxorubicin, methotrexate or cisplatin. The results indicated that OS cells are, in general, very sensitive to PNU-159548. Moreover, PNU-159548 showed a strong activity also in OS drug-resistant cell lines, being able to overcome the classical mechanisms of resistance to doxorubicin and methotrexate as well as, with a slightly lower efficiency, to cisplatin. Simultaneous exposure to PNU-159548 and methotrexate or cisplatin produced mainly antagonist interactions, both in drug-sensitive and in drug-resistant cell lines. Sequential drug exposure analyses showed that the most efficient sequence of administration is the treatment with PNU-159548 followed by methotrexate or cisplatin, without any interval between the administration of these two drugs. Taken together, these data indicate that PNU-159548 may be considered a promising candidate for new chemotherapeutic regimens to be addressed to high-grade OS patients who are poorly responsive to conventional drugs.

**Massive Allograft Reconstruction of Long Bones; 10 Years Follow-Up**

Antonie H.M. Taminiau, R Deijkers
*(Orthopedic Oncology LUMC, Leiden, The Netherlands*

*Introduction:* The use of massive allografts to reconstruct large bone defects in limb salvage for bone tumors has been practiced for many years. Complications, however, are responsible for the failures of massive allografts. Infections, fractures of the graft and delayed union of the graft–host interface and detoriation of cartilage are the most important risks for failure. The long follow-up (>10 years) of allograft procedures in 65 patients treatment and results are presented.
*Material and Method:* Clinical and functional data from all patients treated with allografts for bone tumors in the Leiden Orthopedic oncology service are included in this study. Since 1987 until 1993 in 65 patients with sixty primary malignant (osteosarcoma 22, chondrosarcoma 19, MFH seven, adamantinoma five, Ewing four, other two) and six benign bone tumors (GCT five, ABC one) massive allografts were used. The age of the patients (male 37, female 38) extended from five to 69 years, 2/3 within their fourth decades of life. The allograft procedures consisted in: inlay four, intercalary 25, osteoarticular 23 and allograft-prosthesis composites 13. The sites were femur 27, tibia 18, humerus 14, and radius six. The mean follow-up was 140 months were extracted from the files.
*Results:* At >10 years follow-up of these 65 patients, 48 are disease free, 16 died related to the disease (osteosarcoma eight, chondrosarcoma four (two Olliers), MFH three, Ewing one) and one died of non-tumor-related cause. Only two recurrences occurred (GCT, chondrosarcoma in Olliers disease). In 65 patients allograft procedures no complications were seen in 30 patients (13 died). Complications included skin necrosis one, infection four, delayed-union 17, fracture four, joint detoriation 12 and bone resorption one. Delayed unions (17) were grafted by cancellous bone: successfully in eight, nine required further surgery (for infection two, cartilage detoriation three, fracture three, pseudarthrosis one). Primary infection was seen in four, secondary three; all treated by antibiotics and gentamycin beads. All except one graft were removed, the defect replaced by gentamycin beads and intravenous antibiotics instituted for 6 weeks. After the infection had healed the defect was reconstructed by allografts in two patients, one vascular fibula graft, two defects were replaced by tumor endoprostheses and two underwent disarticulation of their leg. All these patients are now more than 10 years clinical and radiological without evidence of infection. Of 23 osteoarticular allografts, 13 had serious cartilage destruction in time (2–5 years post-operative). Treatment for cartilage destruction consisted in none (three), resurfacing prosthesis (eight), tumor endoprosthesis (one) and arthodesis (one). There were six fractures of the allograft 1–12 years after surgery. Two healed conservatively by plaster, two required revision of the graft, one stabilization by plating and the patient with a fracture and infection underwent disarticulation. Functional outcome (MSTS) is good in 41/48, fair in four (arthrodesis three, intercalary one) and poor in two (disarticulation). Osteoarticular allografts and allograft composites have all some limited range of motion but stable painless joints.
*Summary:* Of the 48 patients alive after reconstruction with allografts for primary bone tumors of the long bones 44 still have their grafts, two received a tumor endoprosthesis and two lost their leg (disarticulation). Final results are satisfactory in almost all.

**Genomic Imbalances Associated with Methotrexate Resistance in Human Osteosarcoma Cells**

Claudia Maria Hattinger, Serra Massimo, Reverter-Branchat Gemma, Pasello Michela, Manara Maria Cristina, Benini Stefania, Astolfi Alessia, Scotlandi Katia, Picci Piero
*(Laboratorio di Ricerca Oncologica, Istituti Ortopedici Rizzoli, Via Barbiano 1/10, Bologna 40136, Italy)*

The major cause of failure of current neoadjuvant chemotherapy protocols for high-grade osteosarcoma (OS), which are mostly based on doxorubicin, methotrexate (MTX), and cisplatin, is the development of drug resistance in about 40% of patients. To identify the genetic basis underlying the MTX resistance phenotype, we have analysed adequate model systems by the use of complementary and innovative techniques. The direct comparison of MTX-resistant variants with their respective parental cell lines by comparative genomic hybridization (CGH) on chromosomes revealed that development of MTX resistance was associated with gain of the chromosomal regions 5q12–q15 and 11q14-qter in U-2OS variants and with gain of 8q22-qter in Saos-2 variants, suggesting the possible involvement of genes located in these regions. For a higher-resolution DNA profiling of MTX-resistant variants compared to their parental MTX-sensitive cell lines, CGH was performed on DNA microarrays (Vysis Inc.) that contained 287 genes known to be frequently amplified or deleted in human cancers. In U-2OS MTX-resistant variants, genes in the chromosomal regions 5q (DHFR, MSH3) and 11q (MLL, RDX) were increasingly amplified in association with higher degrees of resistance to MTX. In Saos-2 MTX-resistant variants, genes in the chromosomal region 8q (EXT1, MYC) were amplified, whereas all genes of the region 10p were deleted. Among the genes found to be gained by CGH on microarrrays, MLL and MYC were studied in more detail by fluorescence in situ hybridization on nuclei and chromosome preparations. Increased copy numbers and the formation of small homogeneously staining regions in association with higher resistance levels to MTX could be visualized in the MTX-resistant variants. In addition, amplification of MLL was found in one cell line inherently 4-fold resistant to MTX compared to U-2OS. These data indicate, that genes underlying the MTX-reistant phenotype of human OS cells, cluster in certain chromosomal regions. Therefore, analyses of these portions of the human genome and the genes so far identified, DHFR, MLL and MYC, might reveal a set of candidate genes to identify patients who develop MTX unresponsiveness and should therefore be considered for innovative, risk-adapted chemotherapy regimens.

## References

[PDF_01618] Hoffman Gwendolyn L., Bock Brooks F., Gallagher E. John, Korte Robert C., Radke Michael W., Reinhart Mary Ann, American Board of Emergency Medicine Task Force on Residency Training Information (2002). Report of the task force on residency training information (2001-2002), American Board of Emergency Medicine.. Ann Emerg Med.

